# A hypothetical intervention on the use of hearing aids for the risk of dementia in people with hearing loss in UK Biobank

**DOI:** 10.1093/aje/kwae452

**Published:** 2024-12-16

**Authors:** Jure Mur, Matthias Klee, Helen R Wright, Alina Solomon, Christine Johnson, Thomas J Littlejohns, Graciela Muniz-Terrera, Anja K Leist

**Affiliations:** Centre for Clinical Brain Sciences, University of Edinburgh, Edinburgh EH1 1HT, United Kingdom; Institute of Genetics and Cancer, University of Edinburgh, Edinburgh EH4 2XU, United Kingdom; Alzheimer Scotland Dementia Research Centre, University of Edinburgh, Edinburgh EH8 9JZ, United Kingdom; Department of Social Sciences, Institute for Research on Socio-Economic Inequality (IRSEI), University of Luxembourg, L-4366 Esch-sur-Alzette, Luxembourg; Department of General Internal Medicine and Psychosomatics, University Medical Centre Heidelberg, 69120 Heidelberg, Germany; Department of Psychology, University of Edinburgh, Edinburgh EH8 9JZ, United Kingdom; Population Health Sciences Institute, Medical Sciences Faculty, Newcastle University, Newcastle upon Tyne NE1 7RU, United Kingdom; Institute of Clinical Medicine/Neurology, University of Eastern Finland, FI-70029 Kuopio, Finland; Division of Clinical Geriatrics, Center for Alzheimer Research, Karolinska Institute, 171 77 Stockholm, Sweden; Ageing Epidemiology Research Unit, School of Public Health, Imperial College London, London W12 0BZ, United Kingdom; Speech and Hearing Sciences, Queen Margaret University, Edinburgh EH21 6UU, United Kingdom; Nuffield Department of Population Health, University of Oxford, Oxford OX3 7LF, United Kingdom; Centre for Dementia Prevention, University of Edinburgh, Edinburgh EH4 2XU, United Kingdom; Ohio University, Athens, OH 45701, United States; Department of Social Sciences, Institute for Research on Socio-Economic Inequality (IRSEI), University of Luxembourg, L-4366 Esch-sur-Alzette, Luxembourg

**Keywords:** dementia, hearing loss, hearing aids, bias, UK Biobank, target trial

## Abstract

Observational studies have reported that hearing aid (HA) use is associated with a reduced risk of dementia diagnosis, suggesting a possible protective effect. However, extant observational studies do not explicitly model causal effects, while randomized controlled trials on the effect of HA on dementia exhibit short follow-up. Here we used self-report, hearing tests, and healthcare records in UK Biobank to design a hypothetical intervention for the effect of HA use on the risk of dementia diagnosis in people with incident hearing loss. HA users exhibited a higher risk of dementia diagnosis than nonusers (risk ratio: 1.43; 95% CI, 1.08-1.88). Associations between HA use and dementia diagnosis were robust across sensitivity analyses (risk ratio: 1.34-1.59), but adjustment for primary healthcare use (risk ratio: 0.77; 95% CI, 0.44-1.33) or primary and secondary care use (risk ratio: 0.68; 95% CI, 0.39-1.18) substantially decreased the observed effect. The decrease in effect estimates upon adjustment for primary (risk ratio: 1.30; 95% CI, 0.95-1.78), and primary and secondary healthcare use (1.30, 0.94-1.78) was smaller when participants with relatively early diagnoses of hearing loss were included in the sample compared to when they were not. While the findings are not conclusive, they suggest residual confounding by healthcare use and dating of hearing loss diagnosis in participants without primary care data in UK Biobank.

## Introduction

Acquired hearing loss (HL) has been associated with dementia and cognitive decline in multiple studies.^1-3^ Due to its high prevalence, moderate effect size in its association with dementia, and the availability of cost-efficient treatment, HL is considered a major modifiable risk factor for dementia. The population attributable fraction from HL—the fraction of dementia cases that are attributable to HL—has been estimated to be the largest (≥7%) among potentially modifiable risk factors in high-income countries.^4-6^ While the pathway between HL and cognitive decline is unknown, several possibilities have been proposed. One involves a common cause, where the same underlying pathology contributes both to HL and to dementia. Another involves a causal link between HL and dementia, mediated through either a decrease in cognitive reserve in an impoverished sensory environment, or an increased cognitive load due to the re-allocation of resources to hearing and away from other cognitive tasks.^7^

Hearing aids (HAs) or cochlear implants can improve hearing in people with acquired age-related HL. If the latter caused dementia, the use of HA to improve hearing in individuals with HL would, on average, be expected to prevent or delay the onset of dementia. In line with a direct causal path, negative associations between HA use and both cognitive decline^8^ and dementia^9^ have been reported. The few randomized controlled trials (RCTs) that have investigated the effects of HL use on cognitive decline have exhibited short follow-up times and have shown no benefits of HA use on dementia risk.^10-12^ As a complementary approach to RCTs, studies increasingly use hypothetical interventions to estimate causal effects in observational data, using the counterfactual framework of emulating a target trial.^13^

In our study, we used observational data from the UK Biobank cohort to estimate the effect of HA use in people with HL on the risk of dementia diagnosis using the counterfactual framework of emulating a target trial.^13^ We tested the effect of HA use in participants with incident HL on the risk of a diagnosis of dementia over a mean follow-up of 12.4 years.

## Methods

### Sample

The UK Biobank (UKB) is a population-based cohort study of approximately 500 000 participants recruited from England, Scotland, and Wales. All participants attended a baseline assessment between 2006 and 2010. At the assessment, participants provided information on demographic, lifestyle, and health-related factors and underwent cognitive tests. All participants consented for UKB to perform ongoing linkage to their electronic healthcare records (EHR). This includes death records, hospital inpatient records and—for 230 082 (45.8%) participants—primary care records. Among the participants, 20 342 (4.0%) participated in a second assessment between 2012 and 2013, and 76 009 (15.1%) in the third assessment from the year 2014 onward. Among the last group, 6993 (1.4%) participants completed the fourth assessment from the year 2019 onward. The sample has been described in detail elsewhere.^14^^,^^15^

### Exposure, outcome, and covariates

**Figure 1:**
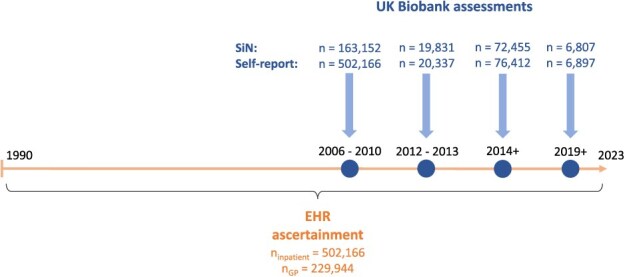
Diagram of sources used to ascertain HL, including the numbers of participants available from each source. The blue circles indicate the assessments when SiN measurements and self-report questionnaires were administered. The exact number of completed assessments differed between participants. SiN was administered to only a subset of participants during the first assessment. The horizontal arrow represents the timeline of EHR availability. Most (>99%) events in primary care occurred after the year 1990 and are available until the years 2016/2017, depending on the nation. Inpatient hospital dates are available from 1997 (England), 1998 (Wales), and 1981 (Scotland) and until 2022. Further information is presented as Appendix S2.

The target population were people with HL, as people without HL should not benefit from HA use. HL was ascertained from at least one of the following sources: speech-in-noise hearing test (SiN), EHR, and self-report (Figure 1). The SiN required participants to correctly identify sounds in the presence of background noise and estimated the signal-to-noise ratio at which half of the presented speech could be correctly perceived. The cut-off of $-$5.5 was used to determine HL, as previously defined^16^^,^^17^ (Appendix S1). For the EHR, we used the first occurrence category in UKB, which combines primary care, hospital inpatient data, death registers, and self-reported medical conditions.^18^ We used first occurrence of “H90 (conductive and sensorineural hearing loss)” or “H91 (other hearing loss).” We also performed a custom search of relevant diagnostic codes (Table S1) in the primary care and inpatient EHR. If all the dates of EHR codes for HL for an individual were missing, HL from the EHR was set to missing. Self-reported hearing difficulties were based on questions on “hearing difficulty/problems” and “hearing difficulty/problems with background noise.” Individuals were categorized as having HL if both questions were answered in the affirmative, as was done previously.^19^ If one of the questions was answered in the affirmative and the other was unanswered, HL from self-report was set to missing. The earliest date of HL across sources was used. The exposure of interest was the initiation of sustained HA use. Thus, the reference group consisted of people with HL without HA use. HA use was ascertained by using self-reported data and the EHR. For self-reported data, the questions on HA use and on the use of cochlear implants were considered. For EHR, a custom list of codes associated with the use of HA or cochlear implants was used (Table S1) to search the primary care and inpatient EHR. The outcome of interest was the diagnosis of all-cause dementia, which was derived by the UKB outcome adjudication team.^20^ The latter classified health outcomes were computed from baseline data, hospital, and death records to provide adjudicated variables to external researchers. All variables used in this study, including their UKB field IDs, are presented in Table S2.

The choice of baseline confounders was based on prior knowledge about common causes of HA use and dementia (Figure S1). In the primary analysis, we adjusted for age, sex, education, socioeconomic deprivation, cognitive ability, hearing ability in the better ear, the source of EHR data, and history of tinnitus.^21^^,^^22^ Age at time zero was calculated using the month and year of birth and the earliest recorded date of HL. Education was based on the completed qualifications and was categorized into no completed degree, a secondary/vocational degree, or a higher degree. Ethnicity was dichotomized as White and non-White. Area-based socioeconomic deprivation was calculated using the Townsend deprivation index (higher values indicate greater relative deprivation)^23^ and was based on data from the national census preceding the participants joining UK Biobank. Cognitive ability (referred to also as *G*—the factor of general intelligence) was calculated as before.^24^^,^^25^ In brief, participants completed tests measuring processing speed and declarative memory during the baseline assessment. A subsample of participants also completed tests of prospective memory, verbal and numerical reasoning, and working memory. These tests were repeated during the first repeat assessment. For the imaging assessments, tests of verbal declarative memory, executive function, nonverbal reasoning, and processing speed were additionally included. *G* was computed separately for each assessment by fitting a confirmatory factor analysis in a structural equation modeling (SEM) framework to the individual cognitive tests that were completed at that assessment. Hearing ability was measured using the SiN as described previously. The source of EHR (England, Scotland, or Wales) was based on the location of the latest diagnosis from the inpatient or—when unavailable—primary care records. The history of tinnitus was determined based on self-report and hospital inpatient records. Covariates used in sensitivity analyses (discussed later) included a history of head injury, mood disorders, social isolation, and healthcare use. The histories of head injury and of mood disorders were determined using the hospital inpatient record. Social isolation was operationalized as previously explained^26^ and involved a dichotomization based on the number of people living in the household, frequency of friend/family visits, and leisure activities. As a proxy for contact with secondary healthcare, we used the mean annual count of hospital spells for each participant in the 5 years preceding time zero and categorized the resulting variable (4 categories: 0, 0-0.2, 0.2-0.5, >0.5). As a proxy for contact with primary care, we used the mean annual number of unique primary care events (prescriptions or diagnoses) in the 5 years preceding time zero and categorized the resulting variable (3 categories: 0-12, 12-24, >24). Postrandomization healthcare contact as an outcome was computed and categorized analogously (Appendix S2). For the recording of confounders that were measured during the assessments, those assessments were chosen that occurred closest in time to start of follow-up but no more than 5 years before or after start of follow-up.

### Data preparation

For all self-reported variables, including covariates, the values for participants who answered “do not know” or “prefer not to answer” were treated as missing. We excluded participants without a date of HL, with a diagnosis of the outcome before HL, with a record of HA use before HL, and—to prevent effective immunity to dementia from the time of HL to UKB entry—those with HL ascertainment before the baseline assessment. We also excluded participants who used cochlear implants at the time of confounder measurement, as they could not be excluded for the application of the SiN. Finally, we handled missing data by performing a complete case analysis, excluding participants who were missing any modeling variables within 5 years of time zero. There were 64 121 observations with missing data (50% of the sample at that stage of data cleaning), 62 412 (97.3%) of which were not administered the SiN during the baseline assessment. The data-cleaning process is presented in Figure S2.

Multiple matching methods and methods for inverse probability weight calculation or distance metrics were tested (Appendix S2). For each analysis, we chose the method that achieved the best balance of covariate distributions between the 2 treatment groups. The methods were assessed by comparing between the exposure groups the standardized mean differences (SMD) in the means, empirical cumulative density functions, and the variance ratios of each covariate. In this process, we prioritized matching methods that did not discard observations. Matching and weighting was performed using the packages *MatchIt*^27^ and *WeightIt,*^28^ respectively. All data preparation and analyses described in the present study were performed in R version 4.3.3^29^ and are available at https://github.com/JuM24/HA-and-dementia-in-UKBB.

### Statistical approach and target trial emulation

The design of the study was based on the counterfactual theory of causality, using the framework of emulating an ideal randomized controlled trial.^13^ An overview of the target trial and the trial emulation is presented in Table 1. Appendix S2 contains additional information on the emulation procedure.

**Table 1:** Specification of the target trial of HA use on the risk of dementia.

**Protocol component**	**Target trial**	**Target trial emulation**
Eligibility criteria	Newly diagnosed acquired HL and no history of dementia	Any indication of HL ascertained through either self-report, SiN, or EHR; the earliest date of ascertainment is considered the date of diagnosis
Treatment strategy	Initiation of HA use and continuation throughout follow-up	Same as for target trial
Treatment assignment	Random assignment to either treatment arm without blinding to treatment	Assignment based on the compatibility of the data to the treatment within a one-year grace period since time zero; we emulated randomization by adjusting for baseline confounders
Specification of time zero (i.e., baseline)	When eligibility criteria were met	Earliest record of HL
Follow-up	Starts at baseline and ends at diagnosis of dementia, death, loss to follow-up, or – for per-protocol effect—cessation of assigned exposure	Same as for target trial
Outcome	Diagnosis of all-cause dementia	Same as for target trial
Causal contrasts	Per-protocol effect; intention to treat effect	Observational analogues of per-protocol and intention-to-treat effects
Analysis	Per-protocol analysis: censor if the participants deviate from their assigned treatment strategy; intention-to-treat analysis	Same per-protocol and intention-to-treat analyses; adjustment for baseline confounders

We used logistic regression to estimate risk ratios (RRs) and computed robust standard errors to calculate 95% confidence intervals. We used doubly robust estimation, where the model for the exposure through matching is combined with outcome regression.^30^ This was done by supplying the weights to a logistic regression model and additionally adjusting for the same covariates that were used to generate the weights. Time-to-event analyses were run using Cox proportional hazards models. The Kaplan–Meier estimator was used to yield survival curves. We weighted the computation of survival curves with the inverse probability weights to adjust for measured confounding. Estimations of RRs and calculations of robust standard errors were performed using the packages *marginaleffects*^31^ and *survey,*^32^ respectively. To approximate the combined effect of covariates on the risk of dementia, we estimated the effect of the normalized propensity scores on dementia diagnosis, stratified by HA use. E-values were calculated as previously described^33^ and were calculated for true values of RR of 1.00 and RR of 0.86, the latter of which corresponds to the estimated effect of HA use on dementia from a recent meta-analysis.^9^

### Sensitivity analyses

To identify the potential source of bias, we performed sensitivity analyses where we altered the analytical approach or added additional covariates. Thus, in addition to the basic intention-to-treat and per-protocol models, our analyses included the exclusion of observations with censoring during the grace period, exclusion of observations for which covariates were measured after time zero, imputation of missing data (Appendix S1), additional adjustment for social isolation and the history of mood disorders, history of head injury, source of HL ascertainment, frequency of inpatient hospitalizations, frequency of primary care contact, and frequency of both inpatient hospitalizations and primary care contact.

To detect potential bias in our estimates, we also applied negative outcome controls (Appendix S2) by performing the analysis of the effect of HA on the following negative outcomes: (1) influenza, (2) diseases of the liver, (3) diseases of the lower respiratory tract, (4) asthma, (5) skin and subcutaneous tissue disorders, (6) infectious and parasitic diseases, (7) appendicitis, (8) hip fractures, and (9) transport accidents.

We also estimated the effects of prerandomization healthcare use on dementia risk and of HA use on postrandomization healthcare use. The latter was estimated by performing pairwise comparisons of each healthcare category to the reference category.^9^^,^^33^ Finally, we repeated the basic intention-to-treat analysis while dropping the requirement of no HL before the UKB baseline assessment. In the latter, HL/HA cases before baseline were not excluded; their dates of HL/HA were set to the date of study start. This was done to explore the causes of substantial differences in the risk estimates between the entire sample and the subsample with primary-care data (see Results section). All sensitivity analyses for dementia and negative outcome controls were performed as intention-to-treat.

## Results

The analytical sample consisted of 59 768 participants with HL, among which 4049 (6.8%) were HA users. HL for 32 988 (55%) participants was determined through self-report, for 24 940 (41.7%) through the SiN, and for 1840 (3.1%) through the EHR (Table S3). Compared with nonusers, HA users were on average older, less deprived, had lower general cognitive ability, poorer hearing ability, lower education, were more likely to be male, White, and have a history of tinnitus. Over the follow-up period (median 12.4 years) using the intention-to-treat approach, 971 individuals were diagnosed with dementia, among which 118 were HA users and 853 were nonusers. In the per-protocol approach, these numbers were 116 and 819, respectively. Descriptive statistics for participants when analyzed according to the intention-to-treat approach are presented in Table 2. The numbers of participants that were censored are depicted in Figure S3.

**Table 2:** Descriptive statistics for variables used in the study when using the intention-to-treat approach.

		**HA** **N (%)**		**Dementia Diagnosis N (%)**	
	**Total** **N (%)**	**Yes**	**No**	**Yes**	**No**
Age, median (IQR)	62.9 (10.5)	65.5 (7.3)	62.7 (10.7)	66.3 (5.3)	62.8 (10.6)
Female sex	29 474 (49.3)	1872 (46.2)	27 602 (49.5)	380 (40.6)	29 094 (49.5)
Education					
No degree	9274 (15.5)	892 (22.0)	8382 (15.0)	323 (34.5)	8951 (15.2)
Secondary/vocational degree	19 613 (32.8)	1196 (29.5)	18 417 (33.1)	276 (29.5)	19 337 (32.9)
Higher degree	30 881 (48.4)	1961 (48.4)	28 920 (51.9)	336 (35.9)	30 545 (51.9)
Deprivation, median (IQR)	−2.13 (4.0)	−2.21 (3.6)	−2.11 (4.1)	−1.63 (4.7)	−2.14 (4.0)
White ethnicity	56 080 (93.8)	3929 (97.0)	52 151 (93.6)	882 (94.3)	55 198 (93.8)
G, median (IQR)	−0.030 (1.48)	−0.16 (1.47)	−0.020 (1.48)	−0.66 (1.72)	−0.021 (1.47)
SiN^b^ median (IQR) mean (SD)	−6 (3)−5.9 (2.3)	−5.5 (3)−5.2 (2.7)	−6 (3)−5.9 (2.3)	−5.5 (2.5)−5.5 (2.7)	−6 (3)−5.9 (2.3)
Data source					
England	54 025 (90.4)	3759 (92.8)	50 266 (90.2)	915 (97.9)	53 110 (90.3)
Scotland	1302 (2.2)	82 (2.0)	1220 (2.2)	2 (0.21)	1300 (2.2)
Wales	1263 (2.1)	53 (1.3)	1210 (2.2)	18 (1.9)	1245 (2.1)
Unknown	3178 (5.3)	155 (3.8)	3023 (5.4)	0	3178 (5.4)
Follow-up, median (IQR)	12.4 (7.9)	12.4 (8.1)	12.3 (7.9)	9.1 (4.3)	12.4 (7.9)
History of tinnitus	23 945 (40.1)	2107 (52.0)	21 868 (39.2)	423 (45.2)	23 552 (40.0)
Social isolation	27 979 (46.8)	1856 (45.9)	26 123 (46.9)	492 (52.7)	27 487 (46.7)
History of mood disorders	6447 (10.8)	445 (11.0)	6002 (10.8)	125 (13.4)	6322 (10.7)
History of head injury	471 (0.79)	41 (1.0)	430 (0.77)	45 (4.8)	426 (0.72)
Annual hospitalizations					
0	31 711 (53.1)	1856 (45.8)	29 855 (53.6)	374 (40.0)	31 337 (53.3)
>0 and ≤0.2	14 117 (23.6)	978 (24.2)	13 139 (23.6)	216 (23.1)	13 901 (23.6)
>0.2 and ≤0.5	6721 (11.2)	527 (13.0)	6194 (11.1)	137 (14.7)	6584 (11.2)
>0.5	7219 (12.1)	688 (17.0)	6531 (11.7)	208 (22.2)	7011 (11.9)
Annual GP appointments^a^					
≥0 and ≤12	11 576 (53.8)	350 (36.1)	11 226 (54.4)	91 (28.6)	11 485 (54.1)
>12 and ≤24	7219 (33.5)	376 (42.0)	6843 (33.2)	128 (40.3)	7091 (33.4)
>24	2734 (12.7)	170 (19.0)	2564 (12.4)	99 (31.1)	2635 (12.4)

a Depicted are the proportions of the total number of participants with primary-care data. For numerical variables, the median and standard deviation are shown; for categorical variables, the counts and percentages are given. The data are shown for the entire sample and separately for different groups of the exposure and the outcome.

b Both the mean and the median are given; for other numerical variables, only the median is shown.

The highest standardized mean difference (SMD)—the difference in the means of each covariate—between treatment groups after matching was exhibited by *G* (SMD = 0.023). The SMDs before and after matching for each covariate in each model are presented in Table S4 and Figure S4. Additionally, Table S5 displays for each model the covariates with the greatest values of the SMD, the variance ratio, and the Kolmogorov–Smirnov statistic.

**Figure 2:**
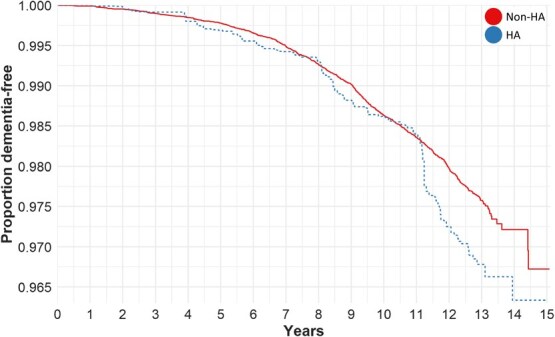
Adjusted Kaplan–Meier curves for the main analyses on the effect of HA on the risk of a diagnosis of dementia using the intention-to-treat approach. The x-axis shows the follow-up time in years, the y-axis shows the proportion of participants who were not diagnosed with dementia. The dotted line and the full line are the survival curves for HA users and nonusers, respectively.

**Figure 3:**
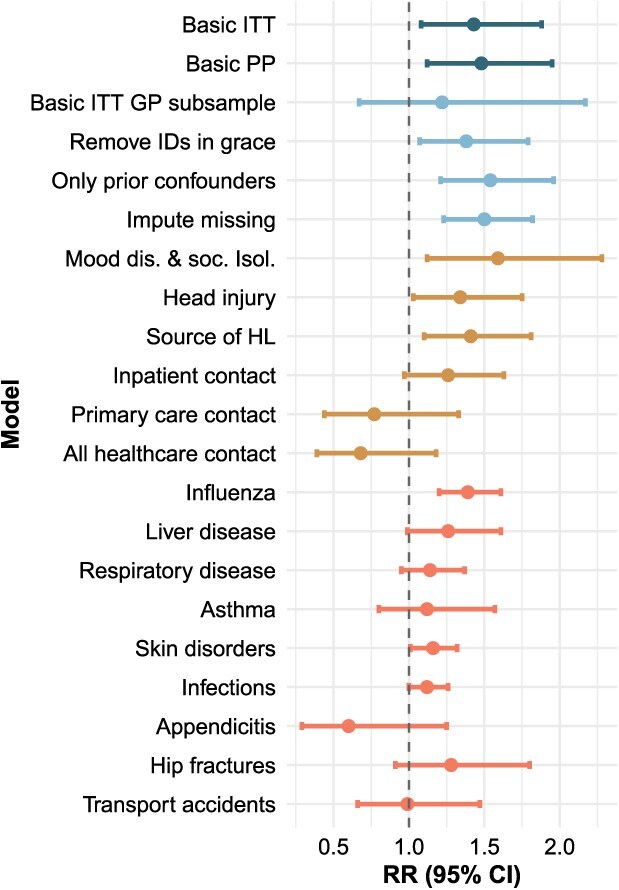
Results from all analyses on the effect of HA use on the diagnosis of a disorder, with colors indicating the type of model. *Basic* refers to the main intention-to-treat and per-protocol models for dementia, *sample change* refers to models where the analytical sample was altered, *extra adjust.* To those where additional covariates were used for matching and adjustment; *neg. control* to analyses of negative control outcomes.

HA users exhibited a higher risk of dementia diagnosis than nonusers in both intention-to-treat (RR = 1.43; 95% CI, 1.08-1.88) and per-protocol (RR = 1.48; 95% CI, 1.12-1.95) analyses (Table S6). Similar results were obtained in the time-to-event analyses (intention-to-treat: HR = 1.31; 95% CI, 0.99-1.74; per-protocol: HR = 1.35; 95% CI, 1.02-1.80) (Figure 2; Figure S5). Among negative outcome controls, 2 of 9 models—for the risk of influenza (RR = 1.39; 95% CI, 1.20-1.61) and cutaneous disorders (RR = 1.16; 95% CI, 1.01-1.32)—were significantly positively associated with HA use, although 7 of 9 models exhibited the same trend as the dementia model. The result of the main dementia model remained significant with similar effect estimates in all sensitivity analyses (RR range: 1.34-1.59) except when the models were additionally adjusted for primary (RR = 0.77; 95% CI, 0.44-1.33) or secondary (RR = 1.26; 95% CI, 0.97-1.63) healthcare use, or both (RR = 0.68; 95% CI, 0.39-1.18) (Figure 3; Table S6; Figure S5). Additionally, greater prerandomization healthcare use was associated with a higher risk of dementia, and HA use was associated with greater postrandomization healthcare use (Figure S6). The combined effect of all covariates used in the basic model on the risk of dementia diagnosis was RR = 1.38 (95% CI, 1.33-1.43). The E-values for the observed effect of RR = 1.43 were RR = 2.21 and RR = 2.71 for a true effect of 1.00 and 0.86, respectively.

When the basic intention-to-treat analysis without adjusting for healthcare use was performed in the primary-care subsample, the point estimate was also reduced (RR = 1.22; 95% CI, 0.67-2.17). The primary-care subsample also exhibited a lower prevalence rate of HA use (4.2%) compared to the rest of the sample (8.2%) (Table S7). When the requirement of no HL before baseline was dropped during data cleaning, the prevalence rate of HA increased (primary-care subsample: 8.2%; rest of the sample: 8.7%; entire sample: 8.5%), and HA was positively associated with dementia risk in both the entire sample (*n* = 66 051; RR = 1.41; 95% CI, 1.15-1.72) and the primary-care subsample (*n* = 27 313; RR = 1.49; 95% CI, 1.06-2.09). The addition of primary healthcare use (RR = 1.30; 95% CI, 0.95-1.78) or the addition of primary and secondary healthcare use (RR = 1.30; 95% CI, 0.94-1.78) diminished the effect estimates, but less so than in the models in which prebaseline cases of HL were excluded prior to analysis (Figure S7).

## Discussion

The present study aimed to quantify the effect of HA use on the risk of dementia in people with incident HL. HA users were at greater risk of a diagnosis of dementia than nonusers. The effect persisted in sensitivity analyses that slightly varied the methodological approach and included additional covariates, except when restricting to those with data from primary care and/or adjusting for primary healthcare use.

Our results are contrary to most previous research. Most authors have reported protective associations for HA use on the risk of dementia or null findings.^2^^,^^34-44^ Few previous studies found HA use to be associated with an increased risk of adverse health outcomes. One found that men—but not women—who used HA experienced higher rates of diabetes and osteoarthritis,^45^ while another one conducted in a subsample of participants from UKB with SiN-based HL (*n* = 18 730) found a positive association between HA use and the self-reported presence of chronic illness.^46^ Studies have also reported associations between HA use and tinnitus.^46^^,^^47^ Furthermore, a previous unpublished analysis in the entire UKB sample found that the rate of dementia in HA users with HL was greater than the rate of dementia in nonusers with HL when each group was compared to people without HL.^48^

One reason for the different results in UKB compared to previous studies could be residual confounding. We controlled for several covariates that could plausibly act as common causes of the exposure and outcome. HA use exhibited significantly positive associations with only 2 of 9 negative controls, although most exhibited the same positive trend. The effect of a continuous unmeasured confounder on the exposure and the outcome would have to be at least RR = 2.2 to explain a biasing from the null. Thus, the total unmeasured confounding would have to be substantially stronger than the effect of measured confounders (RR = 1.38) to bias the true estimate to the extent we observed. The addition of either only contact with primary care or with both primary and secondary care substantially diminished the point estimates. While the CIs still overlapped with the CIs from the basic model, the effect estimates were flipped and now more closely corresponded to other estimates from the literature.^9^ Moreover, prerandomization primary and inpatient healthcare contacts were associated with the risk of dementia, while HA use was also associated with both forms of postrandomization healthcare contact.

However, the effect estimate was smaller in the primary-care subset than in the entire sample, even when healthcare use was not adjusted for. Moreover, the rate of HA use was half as high in the primary-care subset when compared to the rest of the analytical sample. When we removed the requirement of no HL before the baseline UKB assessment, the previously mentioned difference in prevalence rates between the 2 subsamples, as well as the difference in effect estimates for the effect of HA on dementia between the primary-care subsample and the whole sample, were almost nullified. When this alternatively prepared sample was adjusted for healthcare use, the effect estimates were again reduced, but less so than after healthcare use adjustment in the original analytical sample.

Thus, while our analyses were at times underpowered to allow for definitive conclusions, they suggest that the observed results could be due to a combination of 2 factors. First, the original results may be confounded by healthcare use. Most other observational studies on this topic that did not correct for healthcare use found negative associations between HA use and dementia risk, In UKB, healthcare use has been found to likely confound the relationship between another condition (cancer) and dementia.^49^ Moreover, the sample of HA users in UKB is younger than most other studied cohorts of HA users and might represent a particularly vulnerable subgroup of the population of all HA users. These individuals might additionally seek out healthcare more frequently than the rest of the population, contributing to detection bias for medical conditions.

Second, the results could be due to lack of detection or a wrong dating of the beginning of HL. In the UK, most cases of HL are diagnosed by primary care physicians.^50^ Such cases can only be ascertained in the primary-care subsample, while the rest of the sample will include more participants for whom HL was ascertained only through self-report during the UKB assessments. The cleaning step of excluding prebaseline HL cases as per our basic analysis excluded predominantly HL cases diagnosed in primary care, which affected the primary-care subsample differently than the rest of the sample for whom primary care data were unavailable. Thus, some of the effect of HA use on dementia observed in the entire sample could be driven by those participants without primary care whose HL was dated later than was actually the case (see summary of results in Appendix S3). While this would explain the results observed in our study, we are not aware of any studies that tested the accuracy of UK EHR data for HL or HA, thus precluding knowledge on the validity of EHR in this regard. Moreover, it is unclear why the group of participants diagnosed with HL before baseline is driving the positive trend for the association between HA use and the risk of dementia diagnosis.

In addition to the previously mentioned 2 factors, other potential sources of bias exist. One possibility is the mismeasurement of the exposure, the outcome, or both. For this to bias the results, a backdoor path would have to exist between the mismeasured exposure and the mismeasured outcome.^51^ While the algorithm to ascertain dementia used in the present study exhibits a relatively high positive predictive value,^52^ its sensitivity is unknown. If the latter is low due to underreporting or/and underassessment of dementia, and if the misclassification of dementia is associated with exposure status (through the common cause of contact with healthcare, for example), this could contribute to the bias.

Yet another possibility are the potentially different effects of the same treatment in different populations. A recent RCT showed that HA use in those with HL was effective in reducing cognitive decline only in the subsample with more risk factors for dementia.^12^ The UKB sample is wealthier and healthier than the rest of the UK population.^53^ Nevertheless, if HA were protective against dementia only in the more at-risk subsample of UKB, this could explain a diminished effect of HA, not a result at odds with most of the literature.

Our study has several strengths. First, we modeled a causal effect within the counterfactual framework to avoid many biases common in observational research.^54^ The framework of target trials or hypothetical interventions has been increasingly recognized as best practice to analyze observational data.^13^^,^^55^ We explicitly described the proposed causal pathway based on theoretical knowledge and included confounders that were assumed to act as common causes of both exposure and outcome. Second, to identify residual confounding, we applied multiple sensitivity analyses, including the addition of further covariates, application of negative outcome controls, and effect estimation of potential unmeasured confounders. Third, our sample was large, and the follow-up times were much longer than in extant RCTs.

However, we acknowledge several limitations. First, only half of the sample has primary-care linkage, which—as discussed previously—may be contributing to the inaccuracy in HL ascertainment, and the consequent erroneous inclusion of participants with prebaseline HL among participants without primary care data. Second, we could not externally validate the accuracy of HL and HA use as registered in the EHR. EHR likely misses a portion of both HL and HA, as demonstrated by the high proportion of individuals for which HL or HA were ascertained during the assessments but unavailable through the EHR. The lack of data from the EHR also manifested in the absence of information on HA use cessation. Third, the operationalization of the exposure assumed equality between the three methods of ascertainment—self-report, SiN, and EHR. While we did include in our analyses a sensitivity analysis in which the source of HL ascertainment was adjusted for, we would have preferred to have had objective assessment through SiN available for each participant. Fourth, among participants with HA, most in the per-protocol and all in the intention-to-treat analyses were assumed to use HA consistently, but self-reports on compliance or daily duration of HA use were not available. In other data, this assumption can be tested by using proxy variables such as battery purchases.^56^ However, UKB only inconsistently records battery purchases. Moreover, this variable would fail to capture the use of rechargeable HA, often provided from private vendors, which don’t necessitate regular battery purchases. Fifth, while we attempted to emulate an RCT, several characteristics of the sample decreased the quality of the emulation. This included lack of information on the durations of HL and HA use in those with ascertainment during an assessment. We assumed HL to start at the earliest source of ascertainment, but HL may have occurred earlier, reducing the exposure time of HA use relative to length of HL.

Whether improvements in current hearing rehabilitation protocols (eg, using auditory assessments with high real-life relevance such as SiN) can improve HA compliance and provide cognitive benefits should be tested in RCTs. Once these factors are clarified, and as RCTs with long-term follow-up and dementia as the endpoint are rarely achievable, observational data represent a promising resource to test hypotheses about the relationship between HA use and dementia by emulating target trials. Under the assumption of a protective effect of HA against dementia, the results of the present study suggest a relatively strong bias in the HA data in UKB, as we find a detrimental effect of HA use for the risk of dementia diagnosis in the presence of HL. Alternatively, HA use may be only beneficial in more at-risk individuals in terms of age and other risk factors for dementia, which were not well represented in the present data, or HL at earlier ages may be suggestive of central neurodegenerative processes linked to higher dementia risk, which could be—compared to HL at later ages—an overrepresented exposure in the present data. Further research needs to systematically test these competing explanations to evaluate the role of HA use for risk of dementia in people with HL and its associated individual, societal, and economic implications.

## Acknowledgments

We thank all participants of the UK Biobank for providing data for the study and Michelle Luciano (Department of Psychology, University of Edinburgh) for managing UK Biobank data application 10279. The work on this manuscript started while J.M. was also affiliated with the University of Luxembourg, and some of the results have been presented at the GSA 2023 Annual Scientific Meeting and the 27th Nordic Gerontology Congress.

## Supplementary Material

Web_Material_kwae452

## Data Availability

UK Biobank data is available to all approved researchers. The code to reproduce the results is available at: https://github.com/JuM24/HA-and-dementia-in-UKBB.
