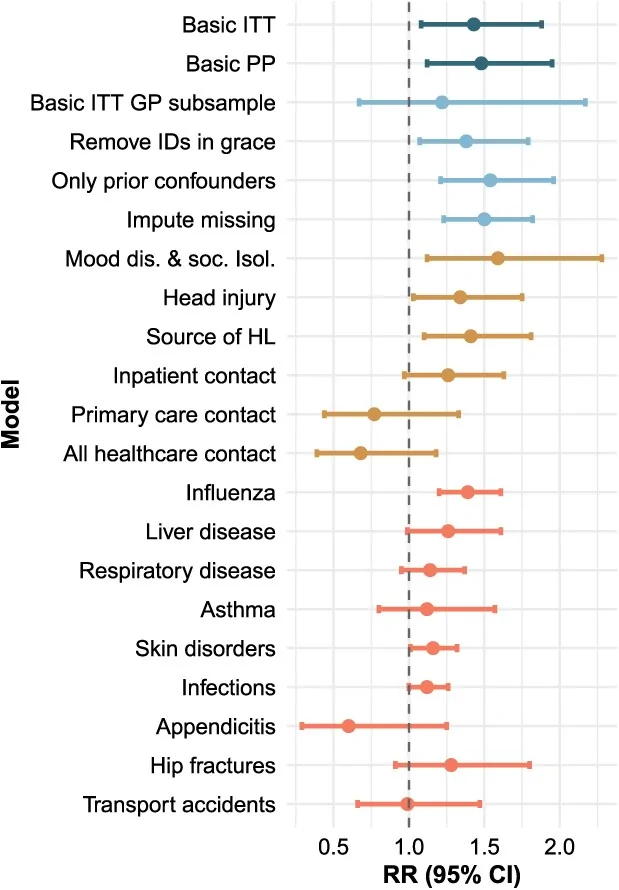# Correction to: A hypothetical intervention on the use of hearing aids for the risk of dementia in people with hearing loss in UK Biobank

**DOI:** 10.1093/aje/kwag190

**Published:** 2026-08-14

This is a correction to: Jure Mur, Matthias Klee, Helen R Wright, Alina Solomon, Christine Johnson, Thomas J Littlejohns, Graciela Muniz-Terrera, Anja K Leist, A hypothetical intervention on the use of hearing aids for the risk of dementia in people with hearing loss in UK Biobank, *American Journal of Epidemiology*, Volume 194, Issue 10, October 2025, Pages 2844–2852, https://doi.org/10.1093/aje/kwae452.

In the originally published version of this manuscript, the legend of Figure 3 was inadvertently omitted.

This error has been corrected.

Figure 3 now reads:

**Figure 3:**
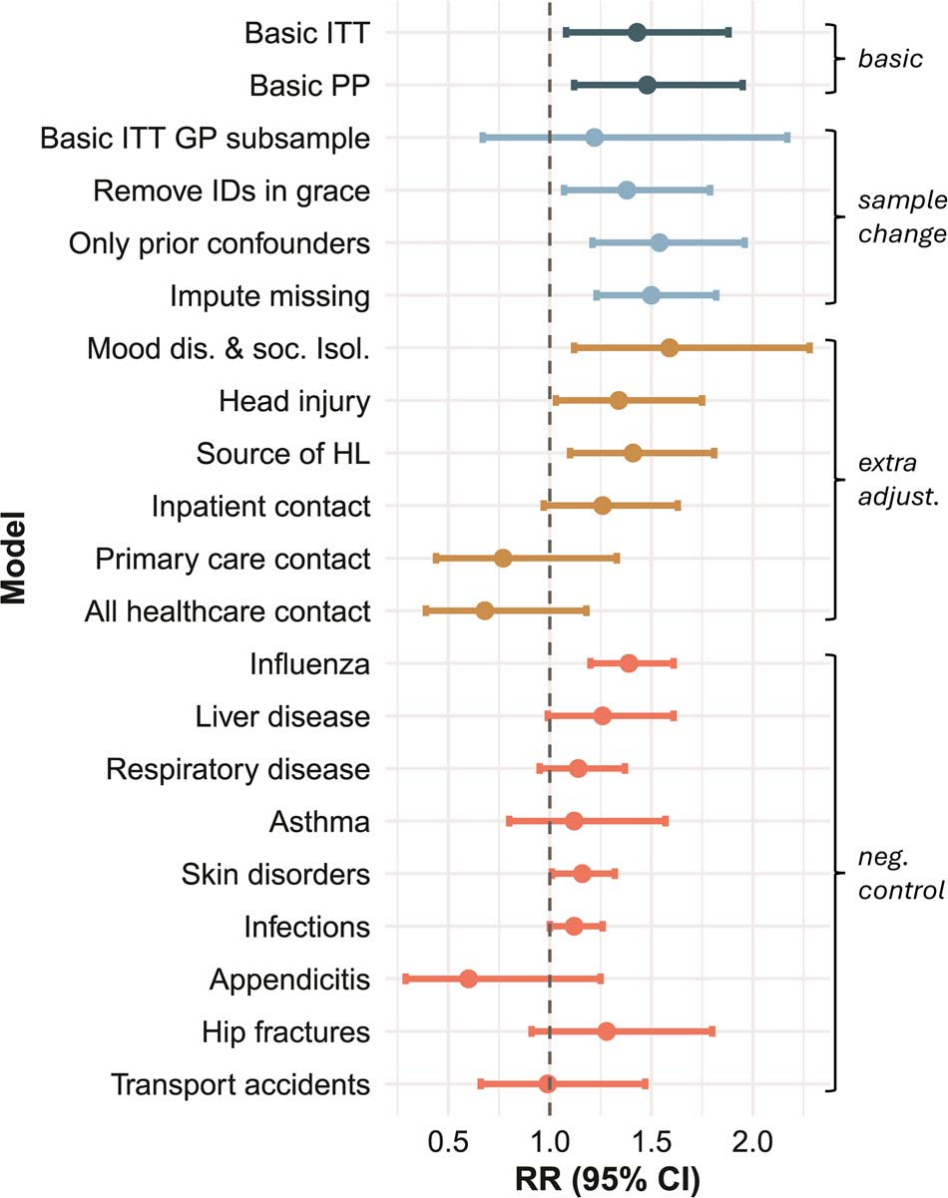


Instead of:

**Figure 3:**