# Supplementary material for: A hypothetical intervention on the use of hearing aids for the risk of dementia in people with hearing loss in UK Biobank
Source: Am J Epidemiol. 2024 Dec 16;194(10):2844–52. doi: 10.1093/aje/kwae452 (PMC12527245; doi:10.1093/aje/kwae452)
Supplement: Web_Material_kwae452 [file web_material_kwae452.zip › supplemental_material.docx]

**Supplementary Material**

**A hypothetical intervention on the use of hearing aids for the risk of dementia in people with hearing loss in UK Biobank**

**Mur, J.^[[1]](#footnote-1),^^[[2]](#footnote-2),^^[[3]](#footnote-3)^ , Klee, M.****^[[4]](#footnote-4),^^[[5]](#footnote-5)^, Wright, H. R.^[[6]](#footnote-6),^^[[7]](#footnote-7),^ Solomon, A.^[[8]](#footnote-8),^^[[9]](#footnote-9),^^[[10]](#footnote-10),^ Johnson, C.^[[11]](#footnote-11)^, Littlejohns, T. J.^[[12]](#footnote-12)^, Muniz-Terrera, G.^[[13]](#footnote-13),^^[[14]](#footnote-14)^, & Leist, A. K.^4^**

**Appendix S1 2**

**Table S2 3**

**Figure S1 4**

**Appendix S2 6**

**Figure S2 7**

**Table S3 9**

**Figure S3 10**

**Figure S4 11**

**Table S6 16**

**Figure S5 17**

**Figure S6 21**

**Table S7 22**

**Figure S7 23**

**Appendix S3 24**

**Appendix S1: additional information on some variables and the dates of EHR data**

*Ascertainment of HL - the speech-in-noise (SiN) test*

The SiN was administered as a computerised test across fifteen rounds, in each of which a spoken digit triplet was presented in the presence of background noise. The level of background noise changed in each round depending on the performance of the participant, and each round yielded a signal-to-noise ratio. The value of this ratio in the last round of the test was termed the speech-reception-threshold - an estimation of the signal-to-noise ratio at which half of the presented speech could be correctly perceived. The test was applied separately to each ear, and users of HA performed the test without their HA. We classified participants as having a hearing impairment if their result of the SiN was above -5.5 (higher scores indicate worse performance) in the better ear, as previously defined^1,2^. Most participants that attended the second, third, or fourth assessments (>95% at each visit) performed the SiN at those assessments. Only 171,774 (34.2%) participants performed the test during the first assessment, because it was introduced as an enhancement after baseline assessments for some of the participants had already taken place. The late introduction of the SiN to the UK Biobank assessment procedure was the main contributor to missing data in our analysis.

*Missing data and imputation details*

The imputation of missing data was performed using multiple imputation by chained equations (MICE) ^3^. We imputed only those observations where the participants chose not to respond or failed to respond to the questionnaires/tests. Thus, observations were not imputed if they were missing due to the variables not having been assessed (as was the case for the SiN for some participants during the baseline assessment) or due to participants not having participated in subsequent assessments. In addition to the variables used in the basic model, the following baseline variables were used for imputation: alcohol consumption frequency (field ID 1558), smoking status (field ID 20116), types of physical activity (field ID 6164) classified as before^4^, and waist circumference (field ID 48). MICE was performed using the *mice* package^5^, utilising the random forest algorithm with 10 imputations, where each imputation used 20 iterations. Among the 64,693 participants with HL that satisfied all inclusion criteria (see **Figure S2**) except non-missingness, 5,293 (8.2%) had some missing data; 599 (0.93%) for education, 89 (0.14%) for socioeconomic deprivation, 1,247 (1.9%) for G, 2,137 (3.3%) for the SiN, 1,219 (1.9%) for tinnitus, and 277 (0.43%) for ethnicity.

*Dates of availability of EHR data*

The latest dates of data availability depended on when the data used in the study were extracted from the EHR and differ between English (HES), Scottish (SMR) and Welsh (PEDW) data providers. For the latest data release, these dates are provided by UK Biobank at [https://biobank.ndph.ox.ac.uk/‌ukb/exinfo.cgi?src=‌‌ Data_providers_and_dates](https://biobank.ndph.ox.ac.uk/ukb/exinfo.cgi?src=Data_providers_and_dates) The dates used in our study were 31.10.2022 (HES), 31.08.2022 (SMR), and 31.05.2022 (PEDW) for all disorders. For participants for which the data provider could not be established (level = “unknown”), the earliest of these dates (PEDW) was used. The primary care data are available for only a subset of 230,082 participants and have not been updated since their release in the year 2018. The dates of right-censoring for the primary care data are April 2017 (Vision) and May 2016 (TPP) for England, March 2017 for Scotland (EMIS/Vision), and August 2017 for Wales (EMIS/Vision). The names in the parentheses refer to the providers of the data for primary care that UK Biobank liaised with to access patient diagnoses from general practitioners^10^.

**Table S2:** variables used in the present study, including their corresponding UK Biobank field IDs.

| **Variable** | **UK Biobank field ID** | **Comments** |
| --- | --- | --- |
| Age | 34, 52 | Age at baseline was calculated using month and year of birth and the first date of HL |
| Sex | 31 |  |
| Education | 6138 | Used highest qualification and transformed into categorical variable with three levels. |
| Socioeconomic deprivation | 22189 | Area-based socioeconomic deprivation is based on the last census before the first assessment; higher values indicate greater socioeconomic deprivation^6^ |
| Cognition/G | 20016, 20018, 20023, 399, 6351, 6373, 23324, 4282, 21004 | We calculated a latent factor of general intelligence from several individual cognitive tests as previously^7,8^ |
| Hearing ability (SiN) | 20019, 20021 |  |
| Source of EHR data | 40022, 41234, 42038, 42040 | When no inpatient hospital source was available, primary care registrations and clinical events were used to determine the source |
| Date of death | 40000 |  |
| Mood disorder | 130890, 130892, 130894, 130896, 130898, 130900, 130902 |  |
| Social isolation | 709, 1031, 6160 | Categorised as previously^9^ |
| Tinnitus | 4803, 41270, 41271, 41280, 41281 | Using self-report and the hospital inpatient record (ICD10: H93.1; ICD9: 3883) |
| Head injury | 41270, 41271, 41280, 41281 | Using the hospital inpatient record (ICD10: S020, S021, S06; ICD9: 800, 801, 850-854) |
| Hearing loss | 2247, 2257, 20019, 20021, 131258-131261, 41270, 41271, 41280, 41281, 42040, 132460 | 132460 was used (in addition to codes in the EHR associated with congenital HL; see **Table S1**) to exclude individuals with congenital HL |
| Hearing aid use | 3393, 4792, 41270, 41271, 41280, 41281, 42040 |  |
| Dementia | 42018 |  |
| Influenza | 131438, 131440, 131442, 131444, 131446, 131448, 131450, 131452, 131454, 131456 | Earliest diagnosis of any disorder with ICD code J09-18 |
| Liver disease | 131658, 131660, 131662, 131664, 131666, 131668, 131670 | Earliest diagnosis of any disorder with ICD code K70-76 |
| Lower respiratory tract disease | 131484, 131486, 131488, 131490, 131492, 131494, 131496, 131498 | Earliest diagnosis of any disorder with ICD code J40-47 |
| Asthma | 42014 |  |
| Skin and cutaneous disease | 131696-131838; only even numbers | Earliest diagnosis of any disorder with ICD code L00-99 |
| Infection | 130000-130344; only even numbers | Earliest diagnosis of any disorder with ICD code A00-99 and B00-B99 |
| Appendicitis | 131604, 131606, 131608 | Earliest diagnosis of disorder with any of the ICD10 codes K35, K36, K37 |
| Hip fracture | 42070, 42080 | Earliest diagnosis of disorder with any of the ICD10 codes S72 |
| Transport accident | 42070, 42080 | Earliest diagnosis of disorder with any of the ICD10 codes V01-V99 |
| Inpatient healthcare contact | 41270, 41271, 41280, 41281 |  |
| Inpatient and GP healthcare contact | 41270, 41271, 41280, 41281, 42040, 42039, 42038 |  |
| Waist circumference, frequency of alcohol use, smoking status, types of physical activity | 48, 1558, 20116, 6164 | Auxiliary variables used in MICE; ascertained during the first UK Biobank assessment. |

**Figure S1** (see also next page): directed acyclic graphs (DAGs) for the analyses. The DAGs are based on theoretical knowledge about the common causes of hearing aid (HA) use and dementia. For simplicity, pathways between adjusted variables and matching between treatment arms are not depicted. **A**: the basic model where all confounders are adjusted for, and which was the basis for the primary analysis of the present paper. Negative outcome controls were assumed to have the same causal structure as dementia and are not depicted. **B**: the primary model for dementia shown in **A**, with the addition of social isolation and the history of mood disorders as mediators between the exposure and dementia. **A** and **B** represent the same causal framework - they differ only in whether social isolation and the history of mood disorders are depicted in the DAG (**B**) or not (**A**). **C**: social isolation and the history of mood disorders as common causes of the exposure and the outcome; here, social isolation and the history of mood disorders are viewed as common causes as opposed to lying on the causal path between HA and dementia (as included explicitly in **B** and implicitly in **A**); this was the basis for the sensitivity analysis in which the history of mood disorders and social isolation at baseline were included as covariates in the analysis for the risk of the diagnosis of dementia. **D:** head injury as a common cause of exposure and outcome; this was the basis for the sensitivity analysis in which head injury before HL was included as a covariate in the analysis for the risk of dementia. **E**: the primary model for dementia as shown in **A**, with the addition of contact with healthcare as a presumed unmeasured common cause of exposure and outcome. There is a backdoor path from HA to dementia through contact with healthcare. **F**: same as **E**, but with the addition of the number of inpatient hospital spells and unique dates of primary care events (either prescriptions or diagnoses) as effects of the contact with healthcare. If the latter was the primary source of bias in our study, additionally adjusting for these variables would reduce the strength of the effect of HA on dementia. The DAGs were constructed using *Dagitty^11^*. HA – hearing aid.


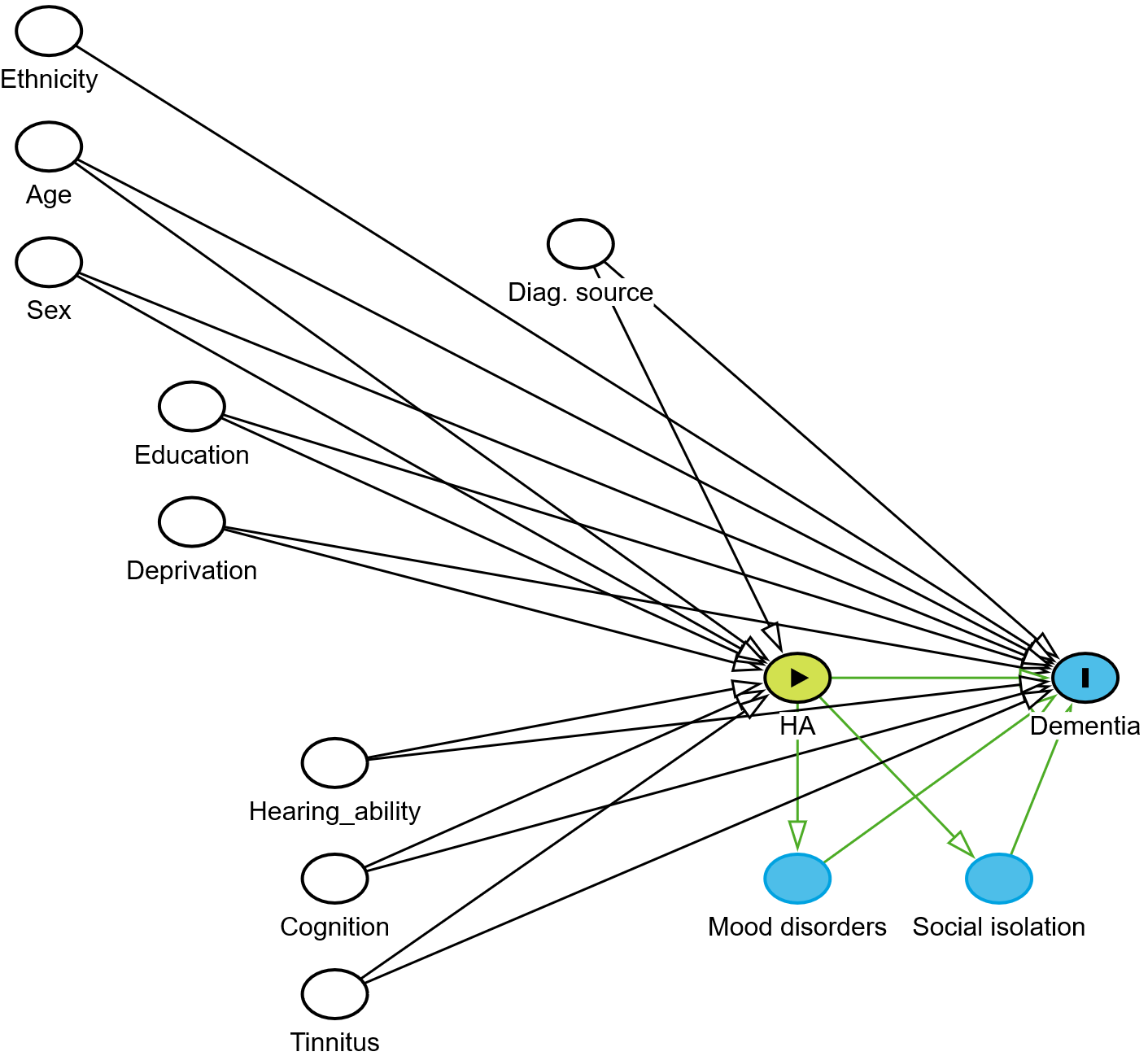

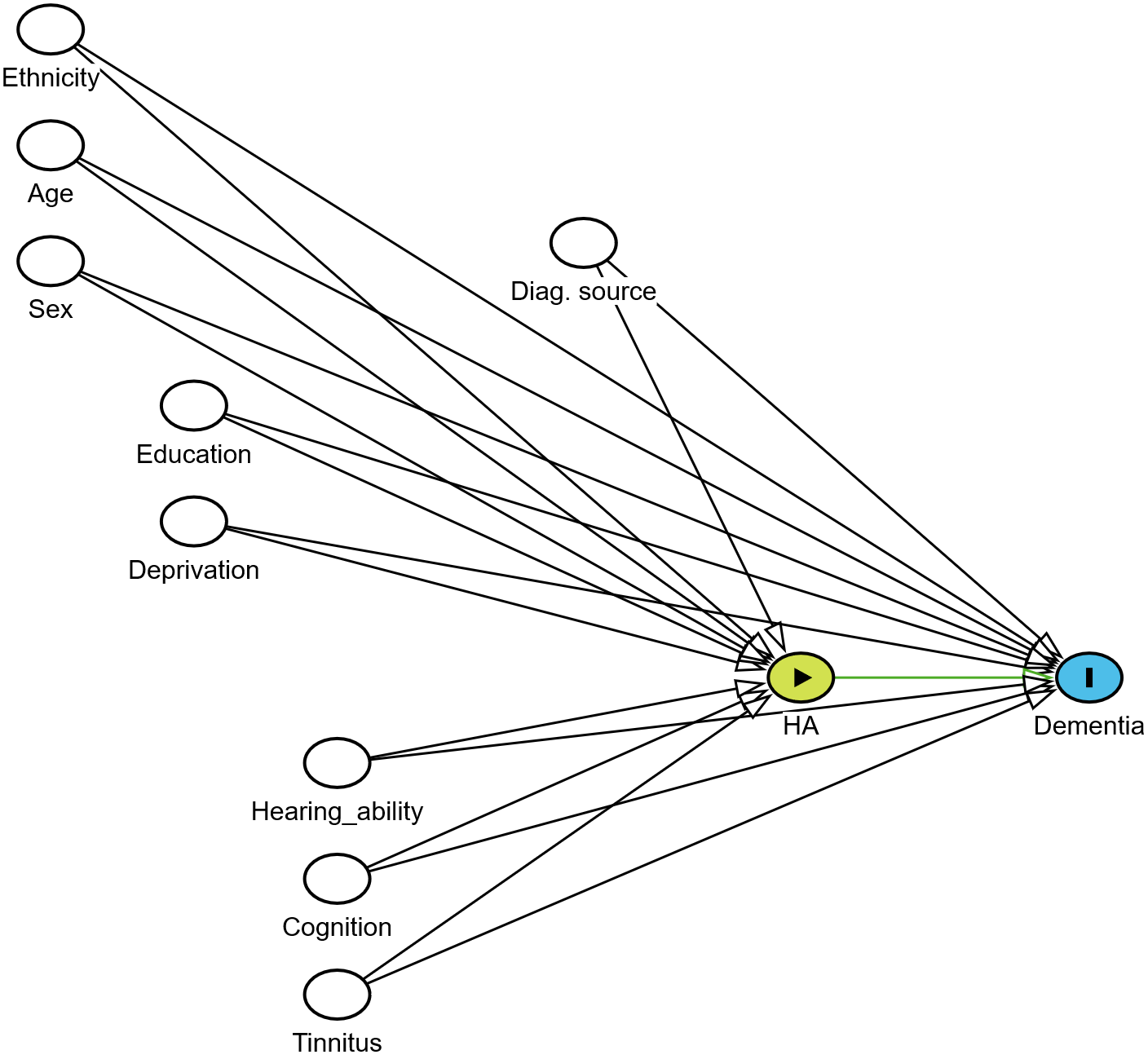


Legend:


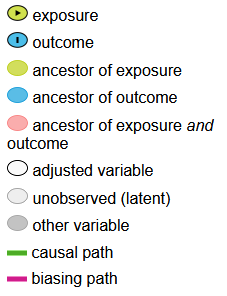

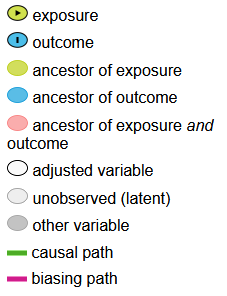


**A**

**B**


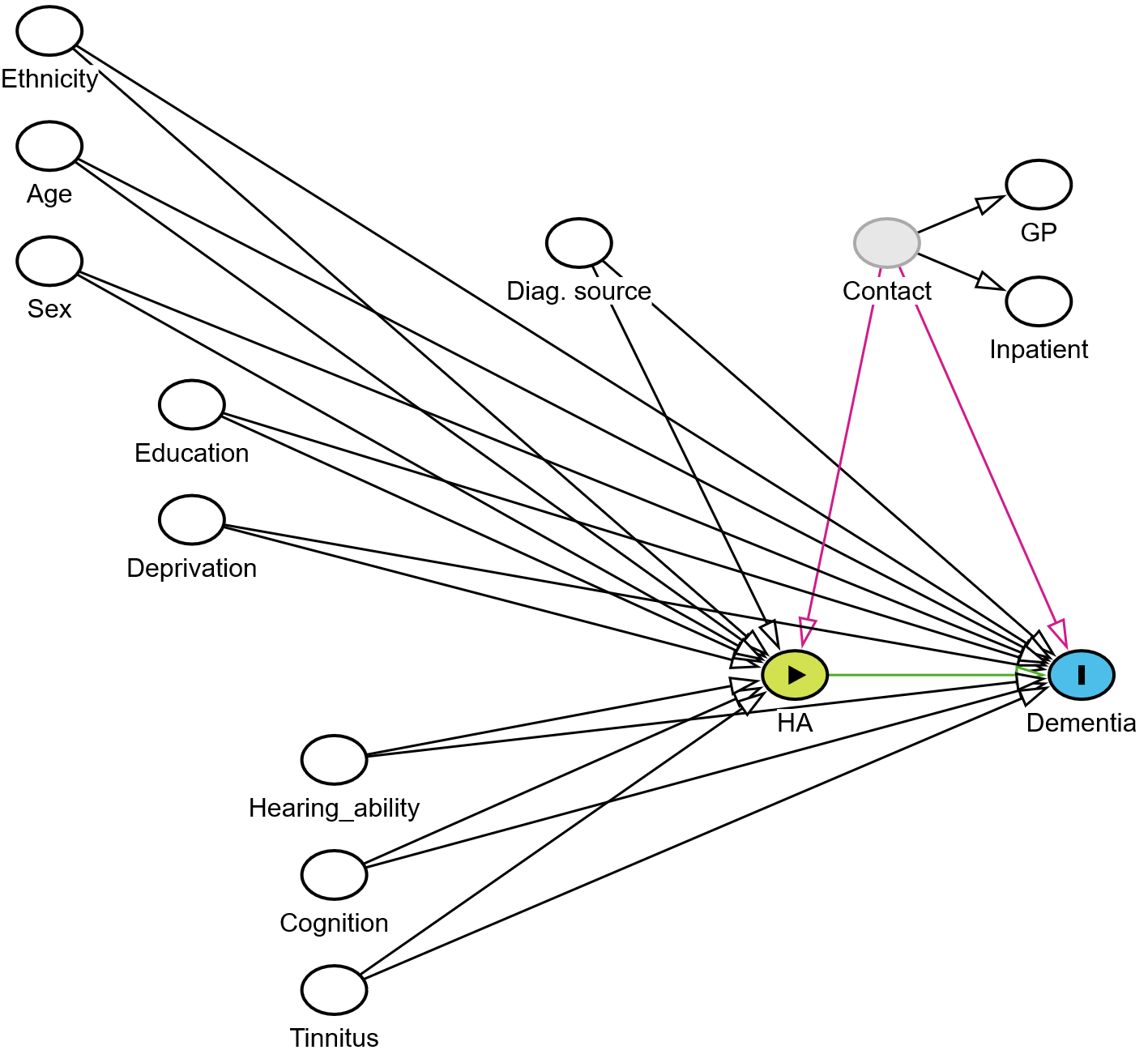

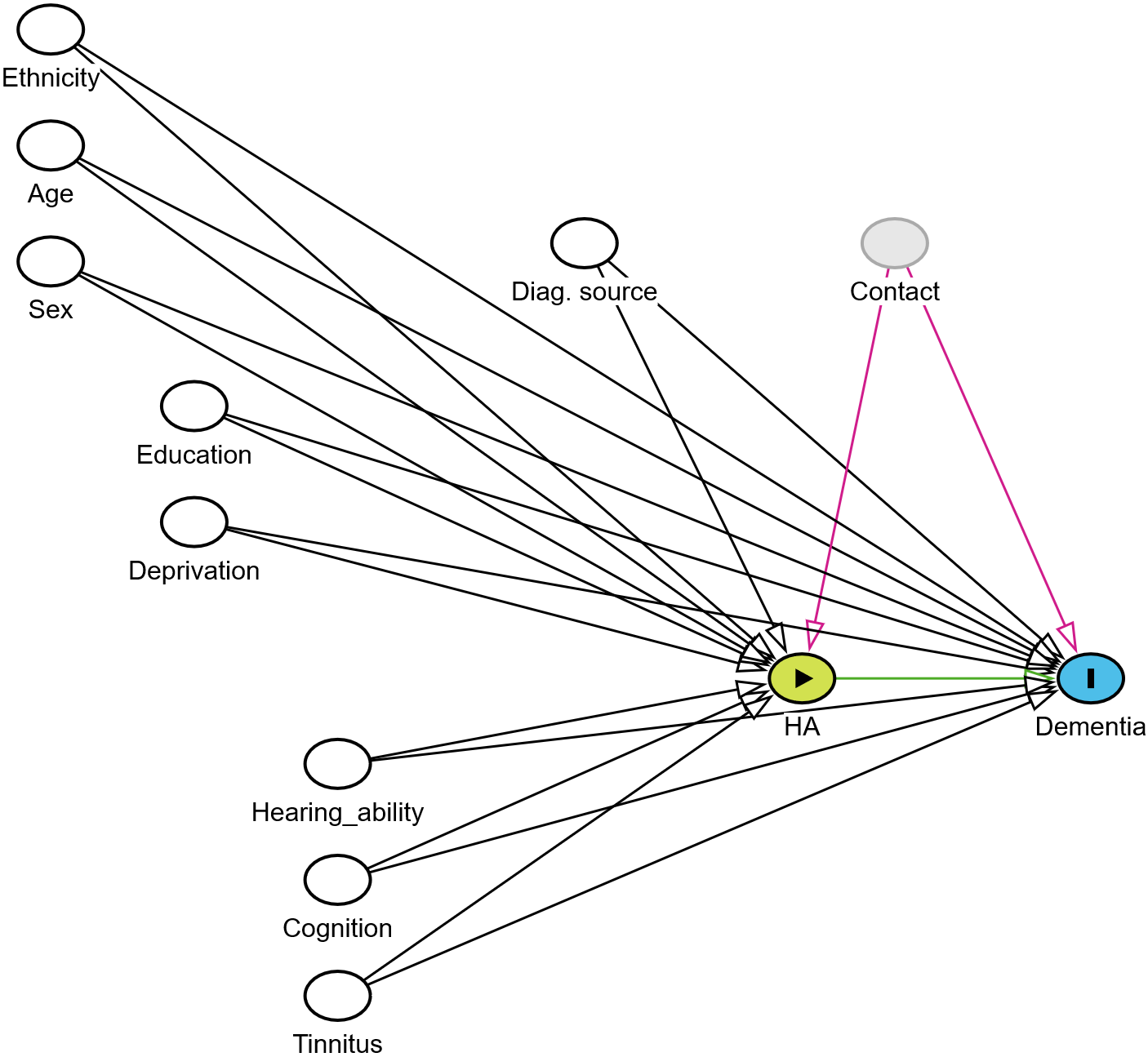


**E**

**F**


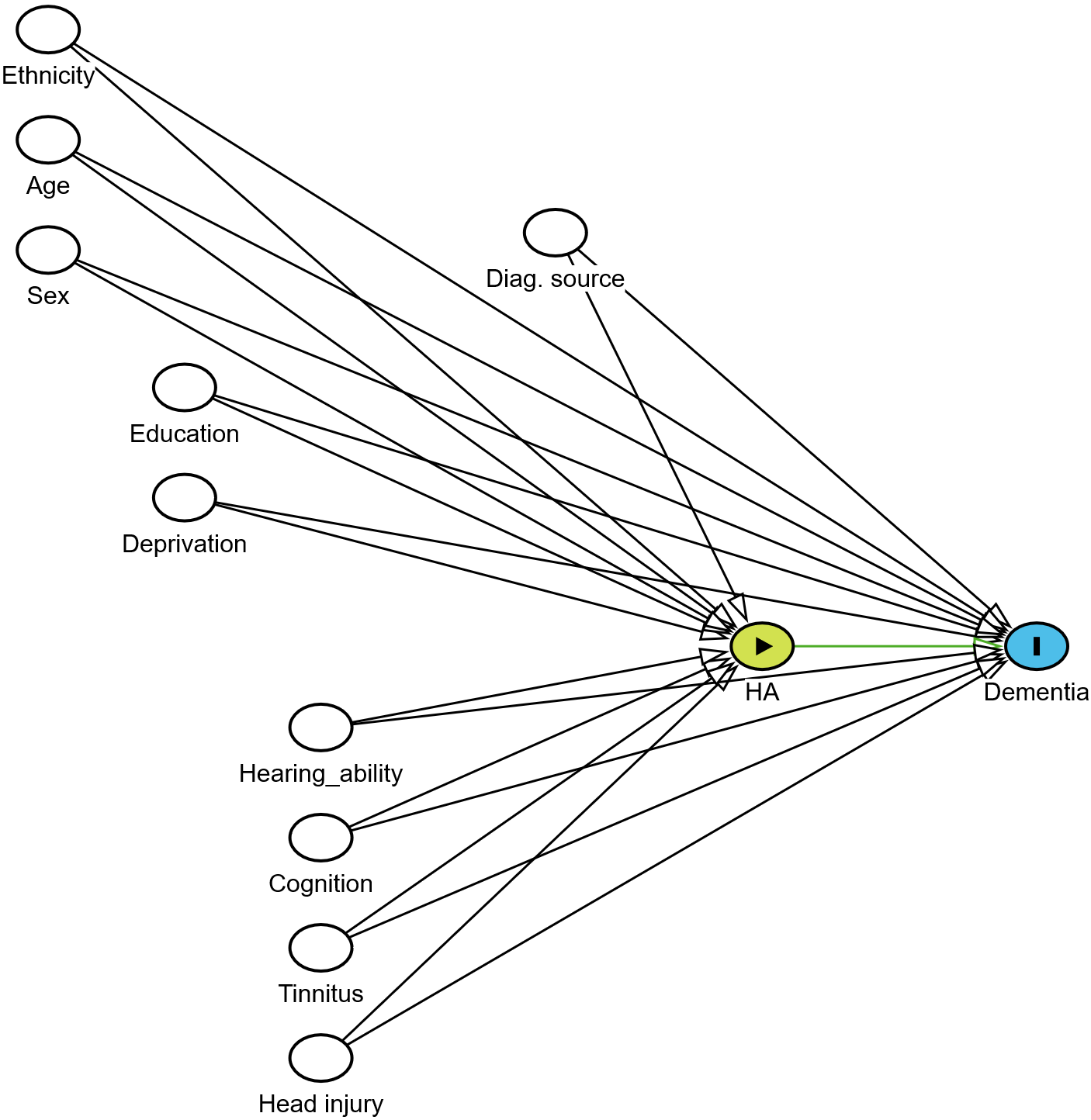

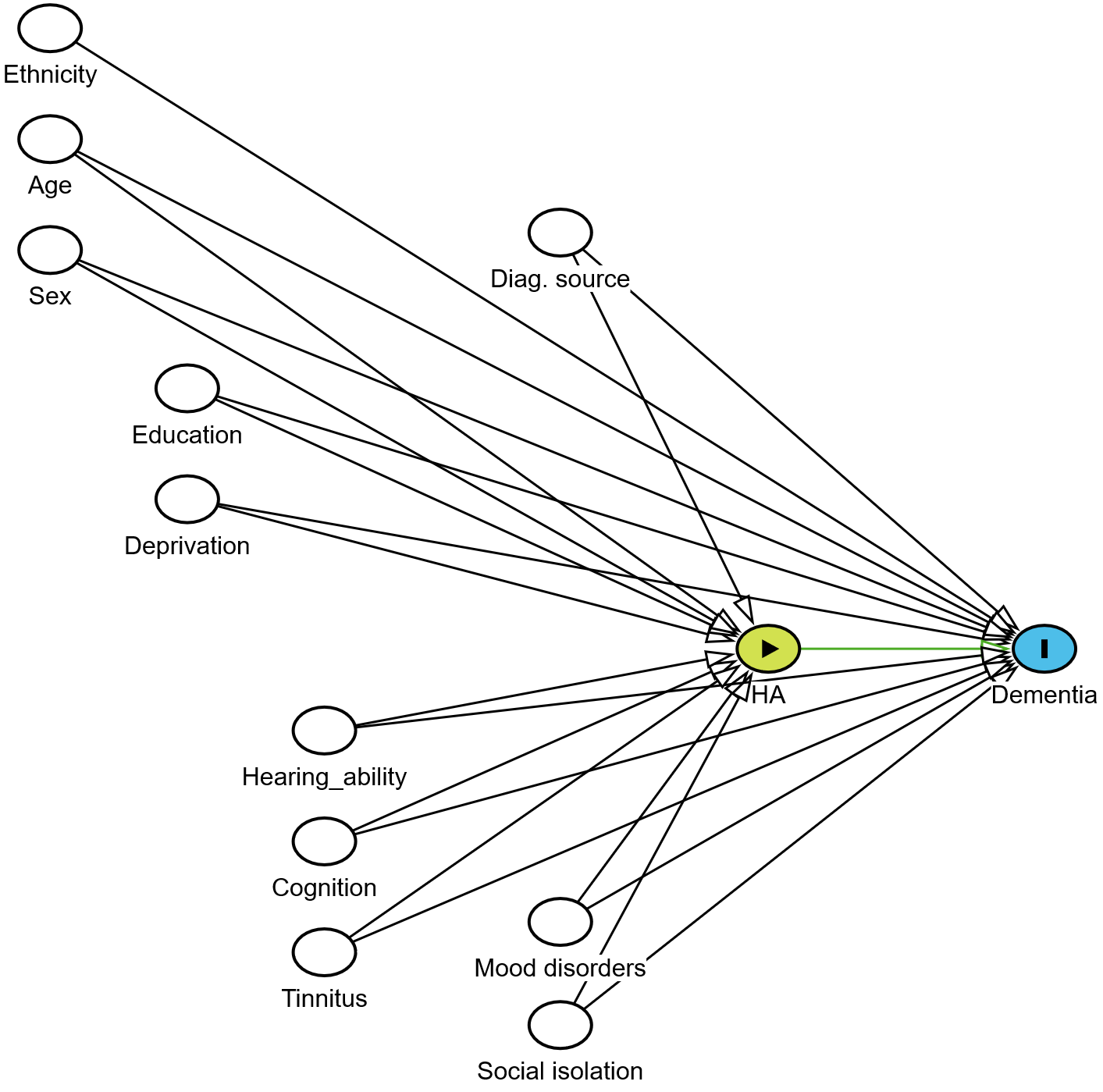


**C**

**D**

**Appendix S2: additional information on the statistical approach**

*Healthcare contact*

For the calculation of pre-randomisation healthcare contact, we considered only those participants with at least one year of primary-care ascertainment in the five years before time zero. This was done for two reasons. First, we assumed that contact with healthcare will be most relevant (for its potential effect on the exposure) closer to when the exposure started. Using all available dates before time zero would inappropriately assign equal weight to *all* periods of healthcare contact before time zero. Second, in 2004, the “Payment by Results” initiative was introduced in England, which funds healthcare based on coding data. After that, the accuracy of diagnostic coding increased in both primary and secondary care^17,18^. By introducing the five-year window, we effectively excluded all events that occurred before the Payment by Results initiative.

For the calculation of post-randomisation healthcare contact, the computation was analogous to the calculation of pre-randomisation healthcare contact: separately for primary-care and hospital visits, the number of unique encounter dates was divided by the number of years of primary-care ascertainment. We again considered only participants with at least one full year of ascertainment. However, we did not limit the period of interest to five years. The period of primary care ascertainment (that was used as the denominator in the calculation of the annual number of primary-care contacts) for both pre- and post-randomisation healthcare contact was computed using a previously defined algorithm^19^; hospital inpatient ascertainment was assumed complete for all participants.

We categorised separately hospital and primary-care utilisation (both pre-randomisation – when used as an exposure – and post-randomisation – when used as an outcome) into four and three categories, respectively, to simplify interpretation, avoid issues with potential non-linear effects between exposure and outcome, and for robustness against outlier values.

*Matching methods tested*

Matching methods: nearest neighbour matching, generalised full matching^12,13^, and propensity score subclassification. Methods for inverse probability weight calculation or distance metrics: generalised linear model, generalised additive model, generalised boosted model, lasso regression, ridge regression, elastic net regression, classification tree, random forest, single-hidden-layer neural network, covariate balancing propensity score, Bayesian additive regression trees (BART) ^14,15^, Euclidean distance, scaled Euclidean distance, Mahalanobis distance, and robust rank-based Mahalanobis distance. We considered covariates with an SMD≤0.1 as well matched.

*Target trial emulation*

Time zero was defined based on the earliest recorded date of HL. For computation of the intention-to-treat effect, follow-up ended on the date of death, dementia diagnosis, or on the latest date of data availability, whichever occurred first. For the per-protocol effect, participants were additionally censored if they switched between treatment groups (**Figure S3**). A one-year grace period starting at time zero was defined. Participants who started HA use within the grace period were assigned to the exposure group; other participants were assigned to the non-exposure group. The grace period was defined for several reasons, some of which have been outlined before^16^. On the one hand, we had to limit the “valid” period of assignment to treatment group because we were interested in *incident* use of HA. On the other hand, it was not appropriate to define incident use as starting at the same time as HL. First, the grace-period approach was supposed to mirror real-world constraints of a true RCT: after the diagnosis of HL, participants assigned to the treatment group would not get HA immediately, but only after a period in which additional hearing tests and HA fittings occurred. Second, the grace period increases the sample size of participants in the treatment group whose data can be used to emulate the target trial. Participants who were censored (due to death or dementia diagnosis) within the grace period were assigned to either the exposure group or the non-exposure group by a random process where the probability of assignment to either group was 0.07 and 0.93, respectively. This probability was based on the prevalence of HA use in the rest of the sample. For the per-protocol effect, participants that started HA use after the grace period were assigned to the non-exposure group and censored at the date of HA ascertainment. Due to a lack of data on HA cessation from the EHR, HA cessation was sourced entirely from self-report. For the intention-to-treat effect, participants were not censored when switching between treatment groups.

*Negative controls*

For a variable to constitute an ideal negative outcome control, the common causes of the exposure and the primary outcome of interest should be identical to the observed and unobserved common causes of the exposure and the proposed negative outcome control^20^. However, there should be no direct causal path from the exposure to the negative outcome control. An association between the exposure and the negative outcome control is an indication that unadjusted confounding might be driving the observed relationship between the exposure (HA) and the primary outcome of interest (diagnosis of all-cause dementia).

**
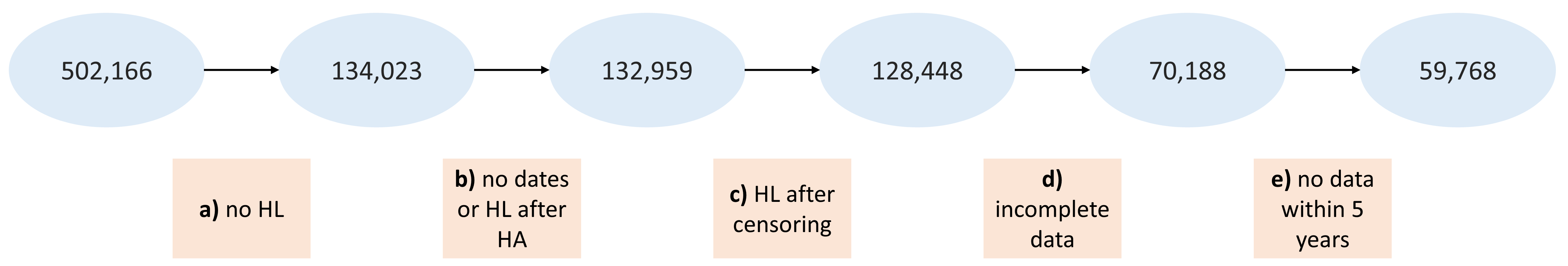
Figure S2**: the data cleaning procedure. The blue ovals contain the numbers of participants at each step of data cleaning. The red rectangles contain short descriptions of criteria for removal from the dataset. The criteria for retention in the dataset at each step were (**a**) evidence of acquired hearing loss (HL) according to at least one data source (self-report, speech-reception threshold, or EHR) without a history of all-cause dementia and without a history of HL before entering the UK Biobank study; (**b**) availability of dates for HL and HA (if applicable) and no HA start after HL; (**c**) no first occurrence of HL after censoring; (**d**) no missing data for any of the variables used in modelling; (**e**) for confounders that were measured during the assessment visits: the presence of a confounder measurement within 5 years of baseline.

**Table S3**: sources of hearing loss (HL) and hearing aid (HA) ascertainment. The percentages are calculated based on proportions of all individuals identified as having HL (for the HL part of the table) and of all individuals identified as using a HA (for the HA use part of the table). The green shaded regions display the n and % from each category that were the *first* occurrence of HL (top) or HA (bottom). The former were effectively used to calculate time zero and the start of HA use. The unshaded regions indicate *any* occurrence of HL (top) or HA (bottom) for the various sources.

|  | **N (%)** |
| --- | --- |
| **HL** |  |
| Self-report | 32,988 (55.2) |
| SiN | 24,940 (41.7) |
| EHR | 1,840 (3.1) |
| Self-report | 40,617 (68.0) |
| SiN | 30,859 (51.6) |
| EHR | 6,081 (10.2) |
| Only self-report | 24,981 (41.8) |
| Only SiN | 17,372 (29.0) |
| Only EHR | 1,279 (2.1) |
| Self-report and SiN | 12,987 (21.7) |
| Self-report and EHR | 4,302 (7.2) |
| SiN and EHR | 2,153 (3.6) |
| Self-report and SiN and EHR | 1,653 (2.8) |
| **HA use** |  |
| Self-report | 3,788 (93.6) |
| EHR | 44 (1.1) |
| Random assignment* | 217 (5.4) |
| Self-report | 3,800 (93.9) |
| EHR | 261 (6.4) |
| Only self-report | 3,571 (88.2) |
| Only EHR | 32 (0.79) |
| Self-report and EHR | 229 (5.7) |

**Note: for those participants that were censored during the grace period.*

**Figure S3**: the numbers of participants in each group that were censored due to the various endpoints. *Dem*, *Dead, HA/non-HA, Loss,* and *End* refer to censoring due to diagnosis of dementia, death, switching between treatment groups, loss to follow-up, and end of data availability, respectively. The **top** figure displays the numbers for the intention-to-treat analysis, while the **bottom** figure displays the numbers for the per-protocol analysis.


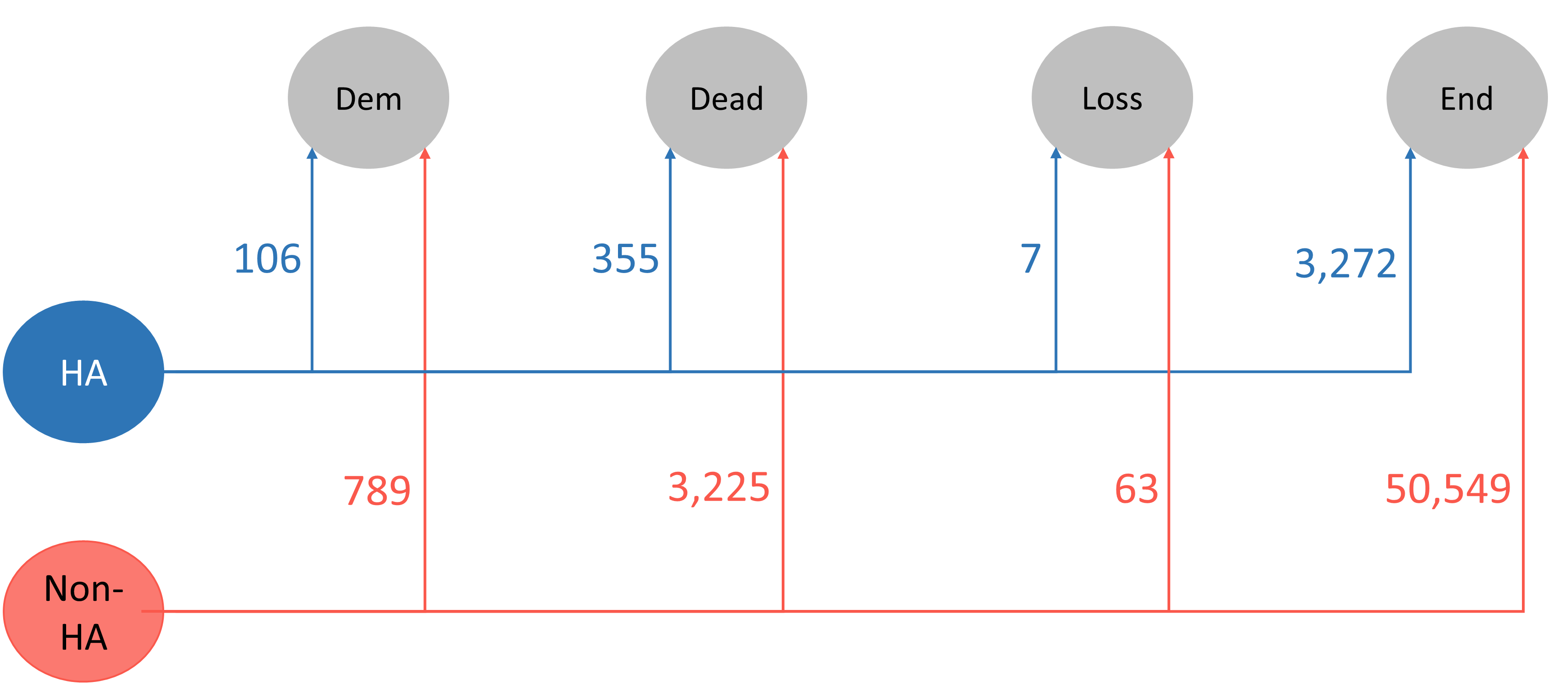

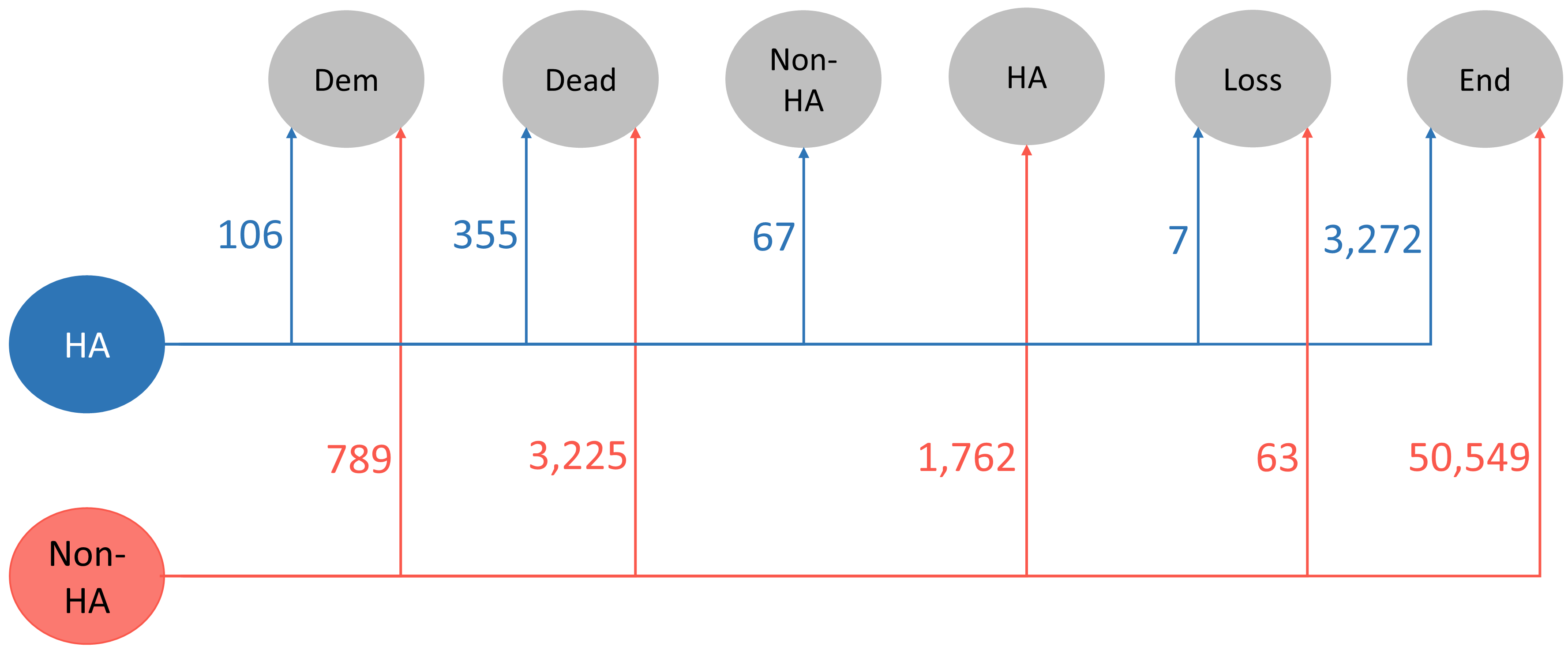


**Figure S4** (see also next three pages): standard mean differences (SMDs; depicted with Love plots^22^) for each model in the total and matched samples. The x-axis indicates the magnitude of the SMD, while the y-axis indicates the difference in propensity scores and covariates. The red dots are absolute SMDs before matching and the blue dots are absolute SMDs after matching. The model numbers are the same as in **Table S4**. Asterisks indicate categorical variables; the dotted vertical lines depict SMD=-0.1 and SMD=0.1. In (5), the horizontal lines depict the ranges of SMDs across imputations.


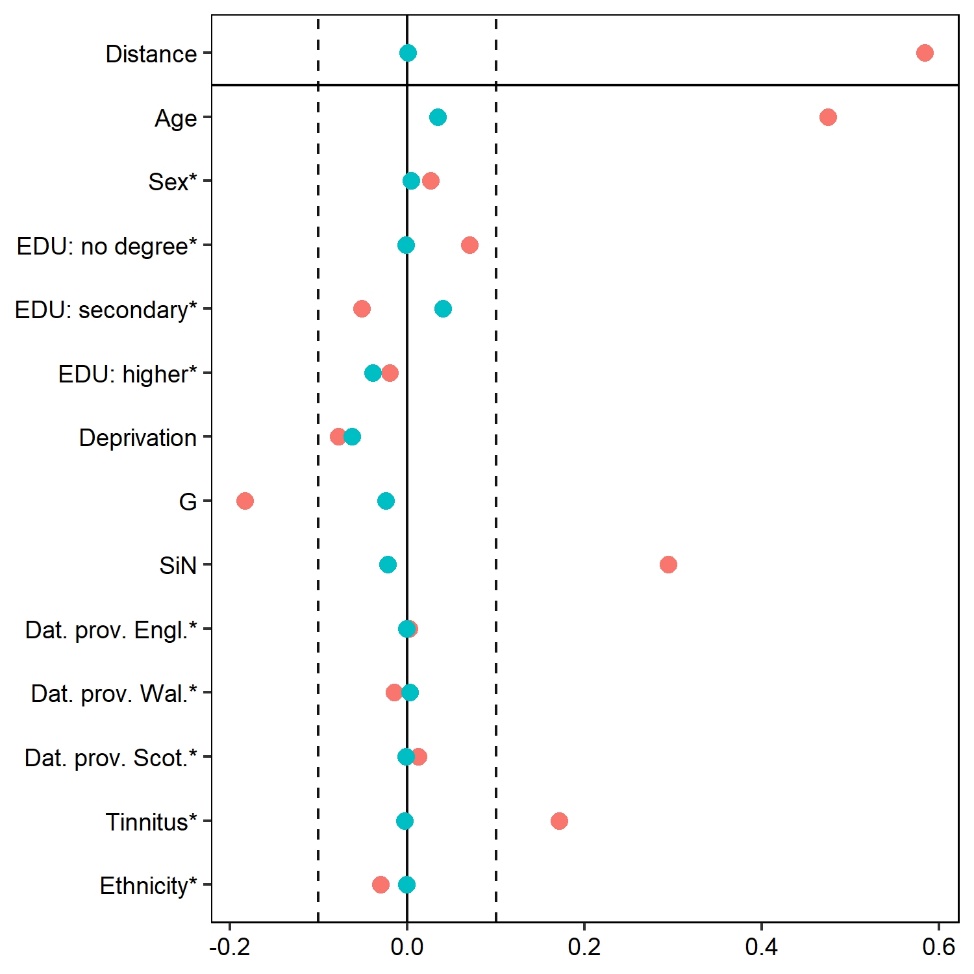

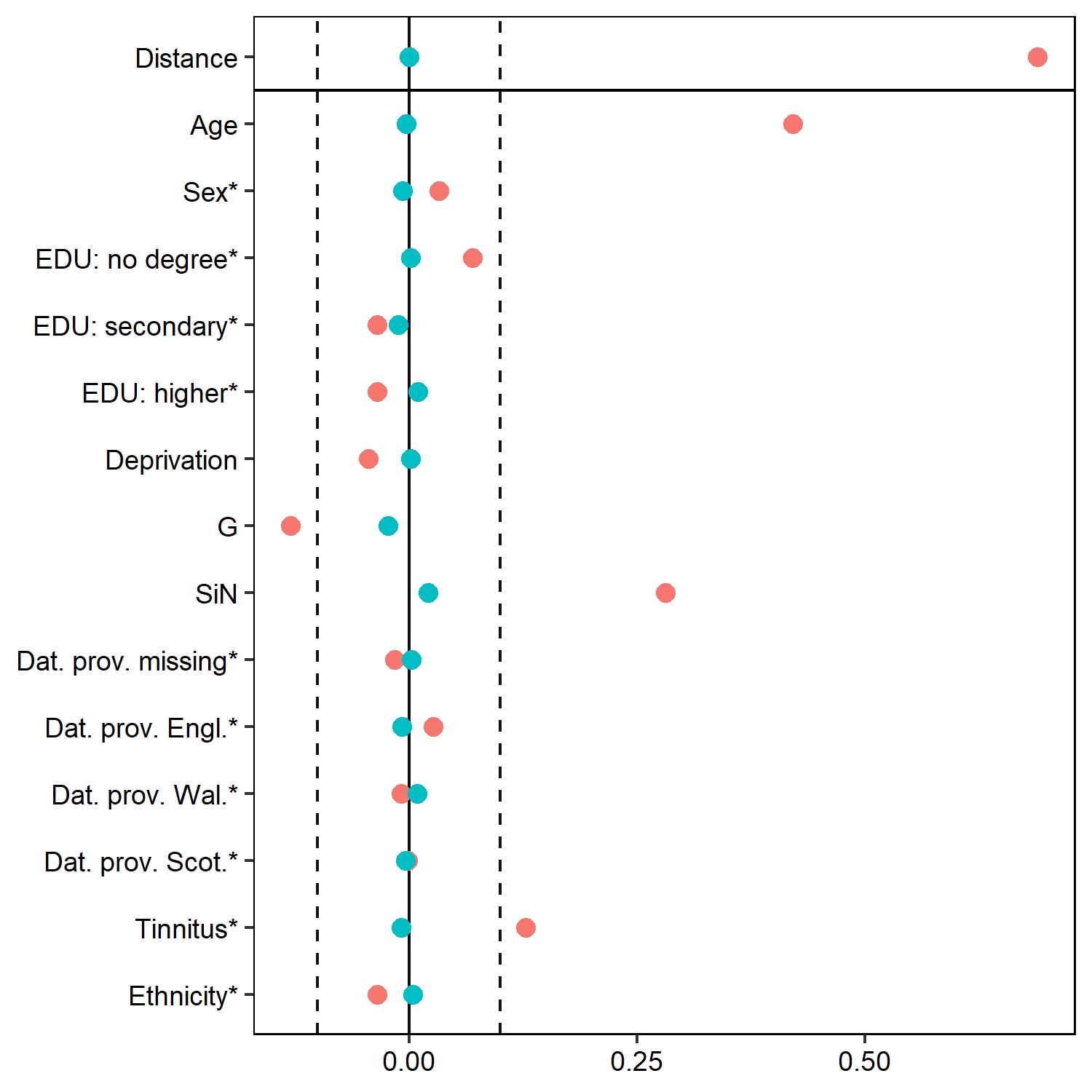


**1**

**1.1**


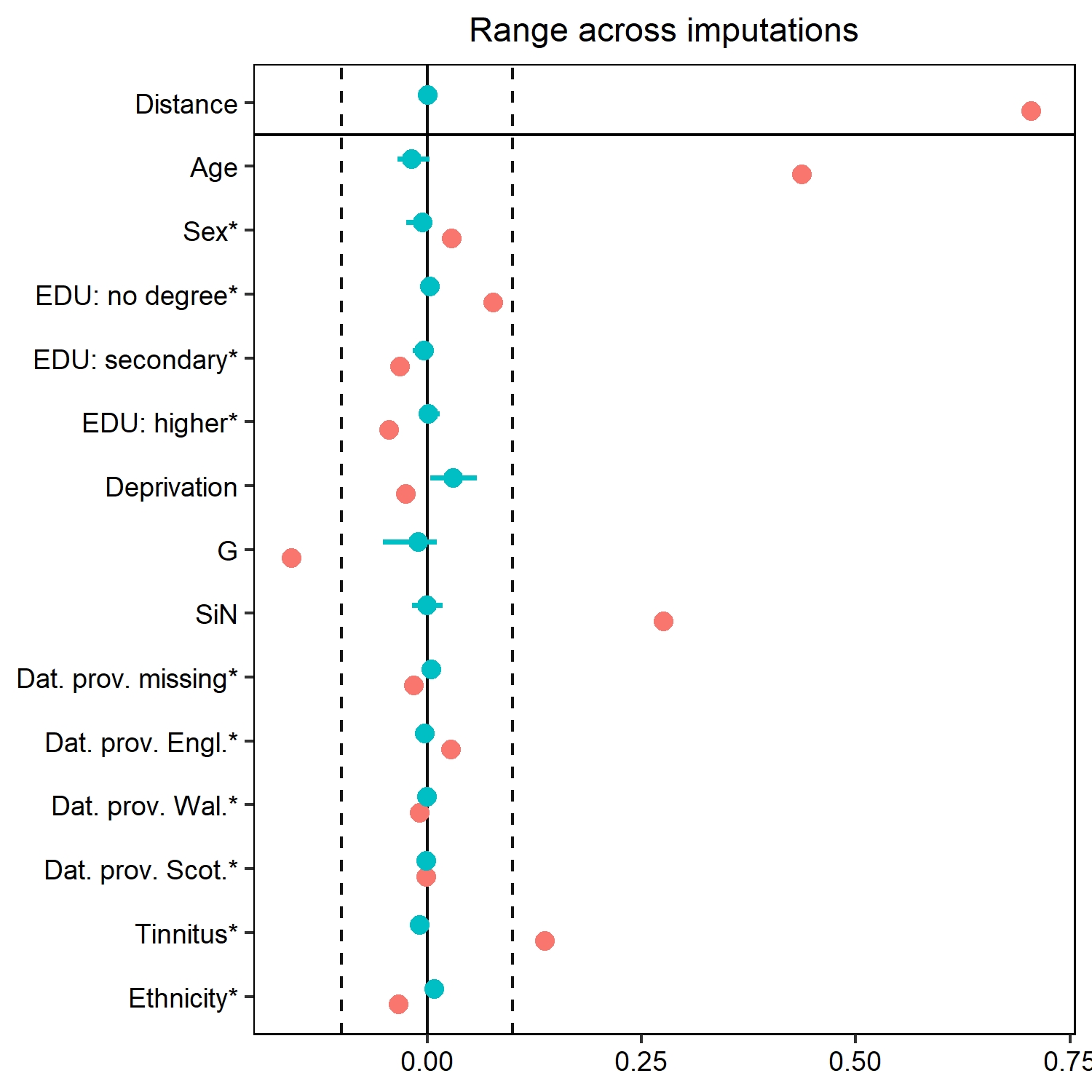

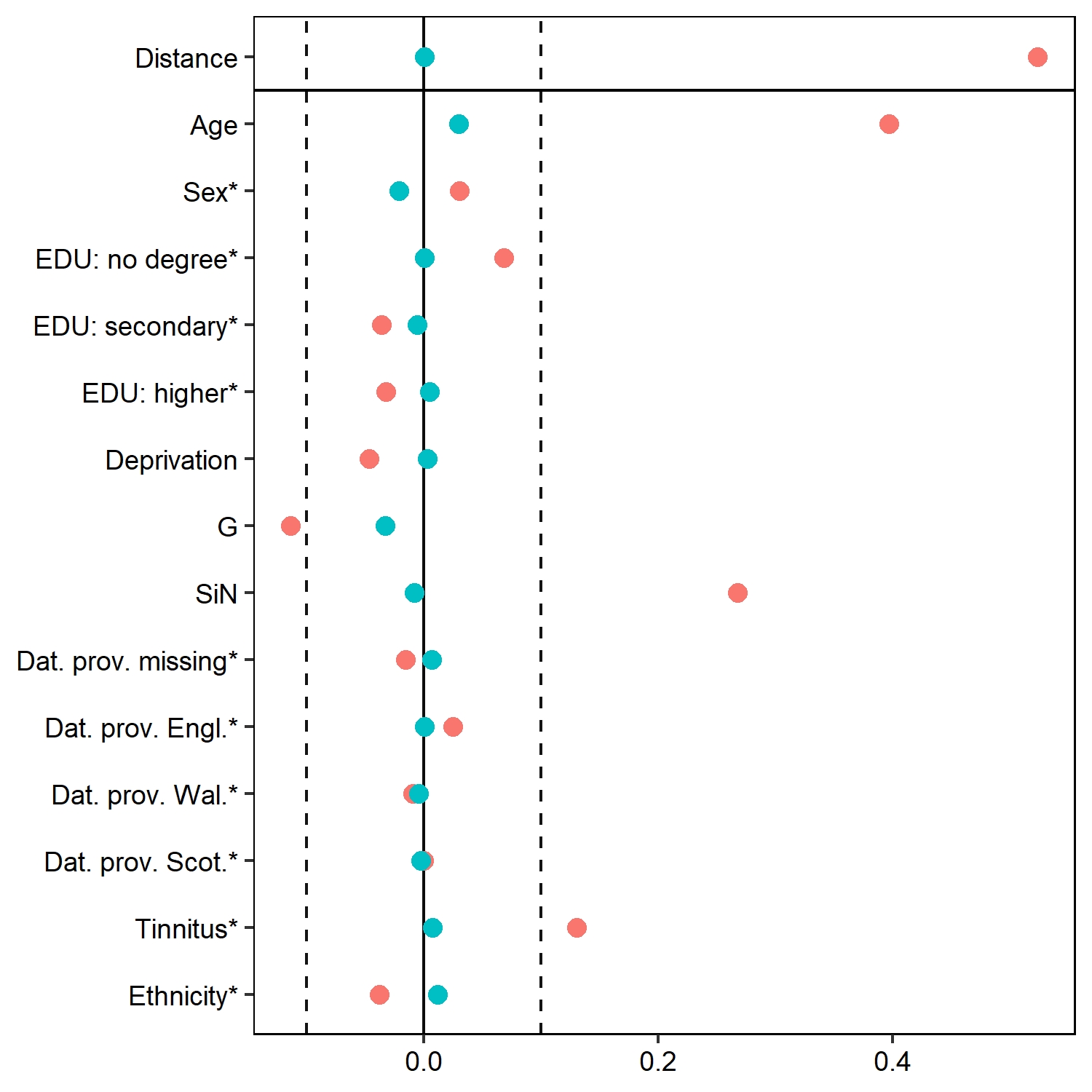


**4**

**5**


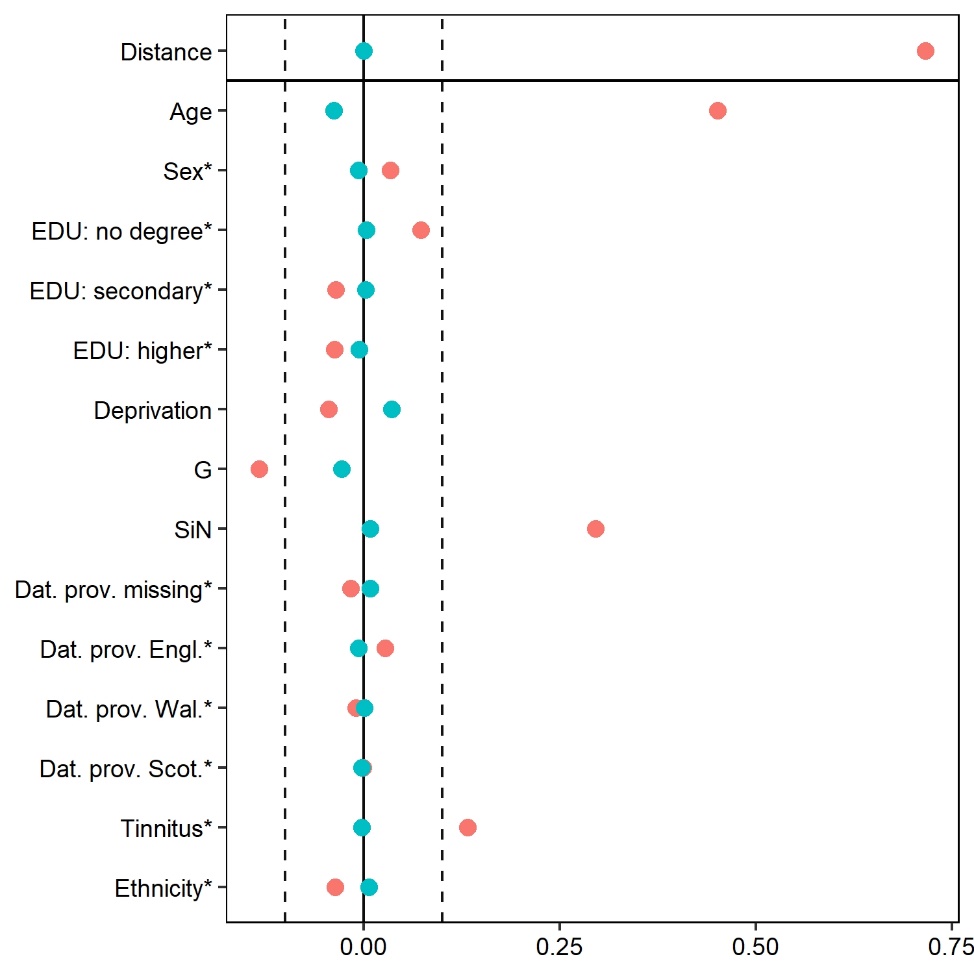

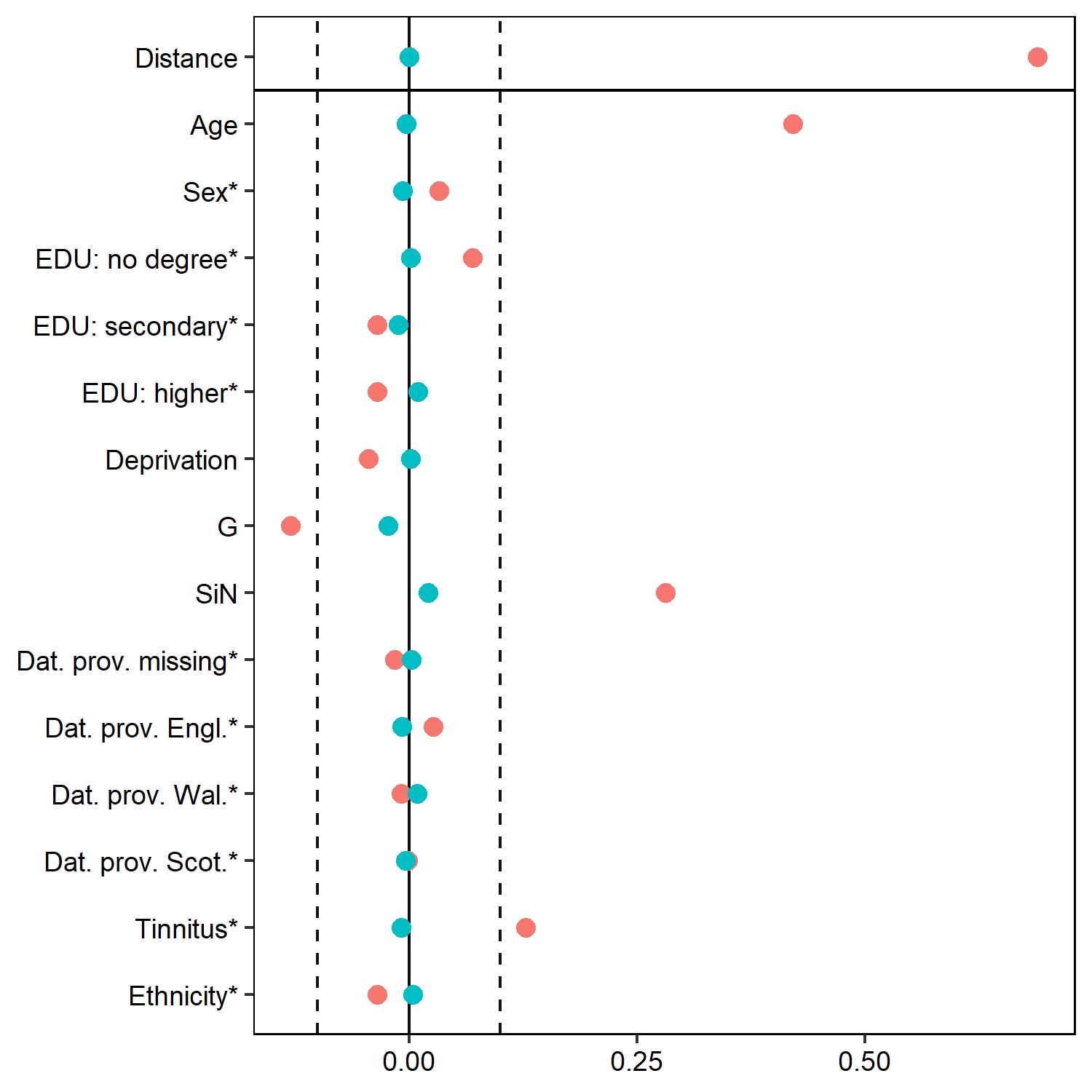


**2**

**3**


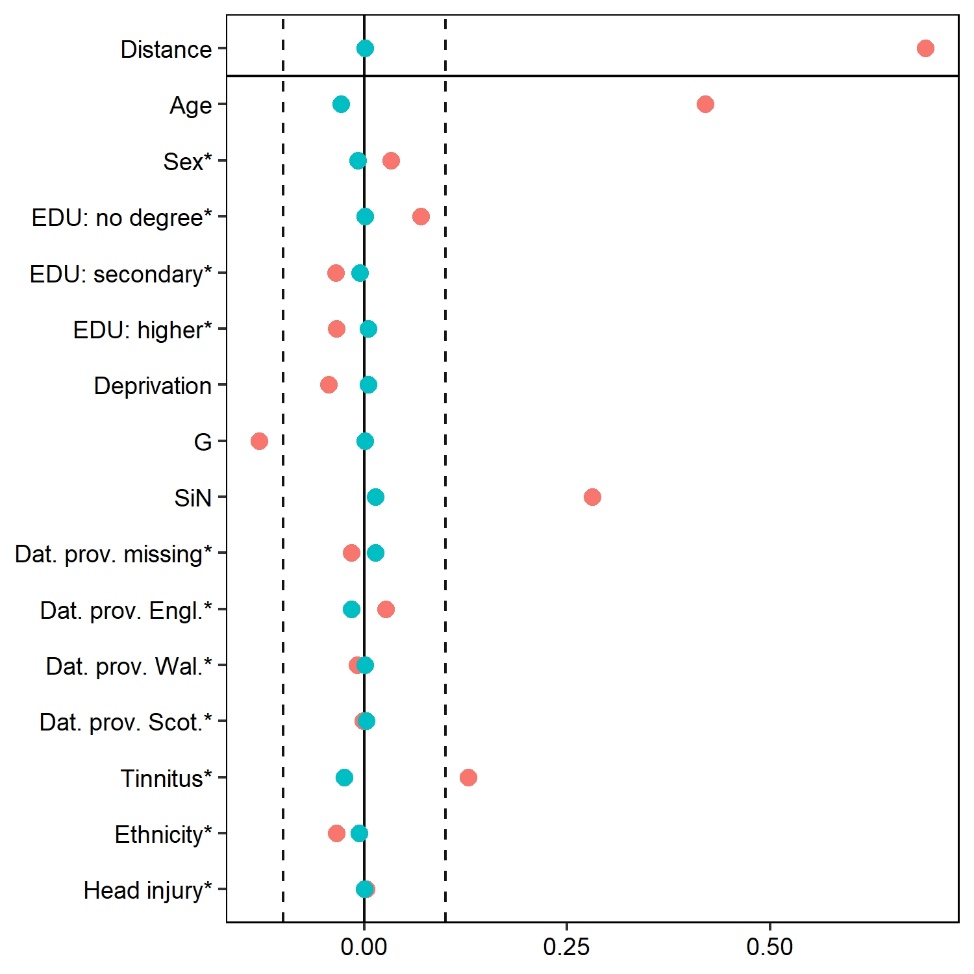

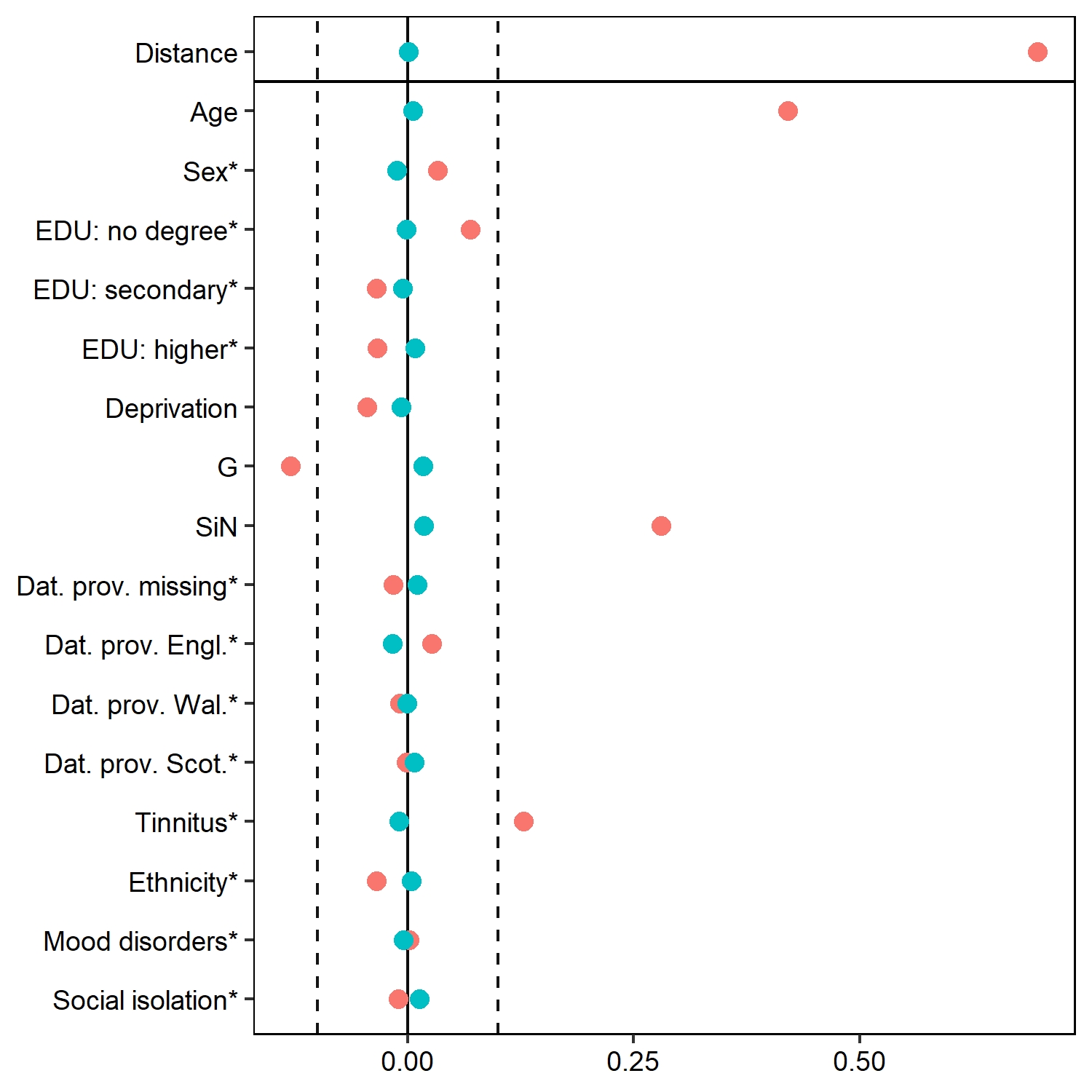


**60**

**7**


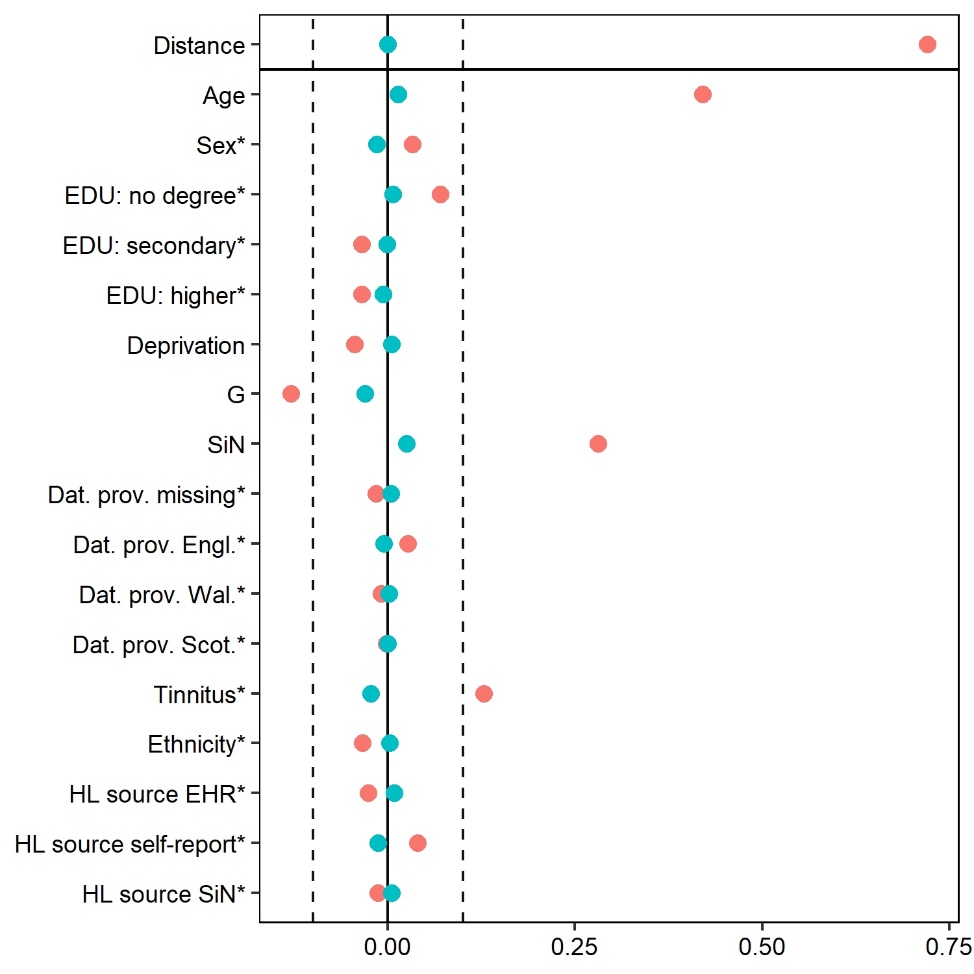

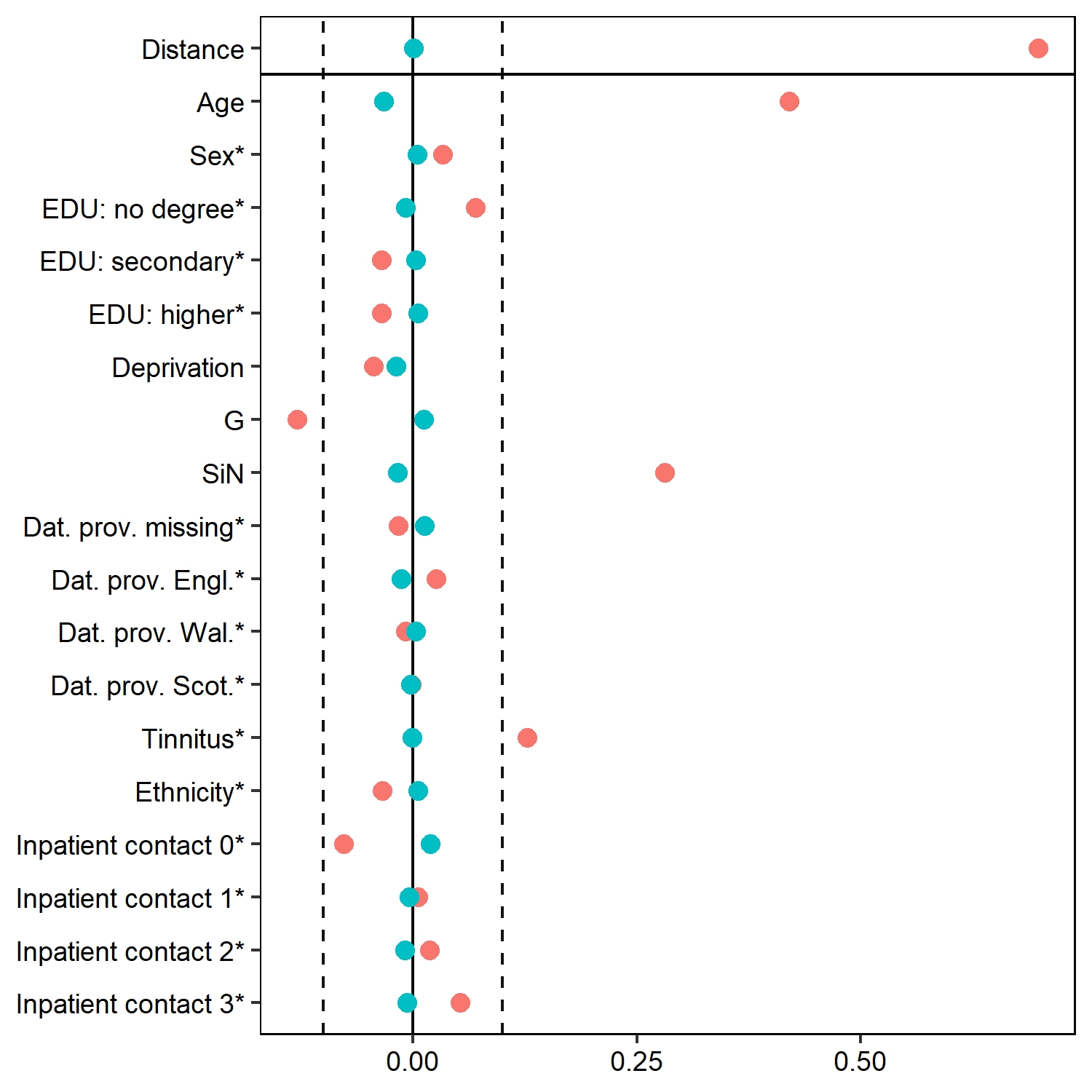


**8**

**9**


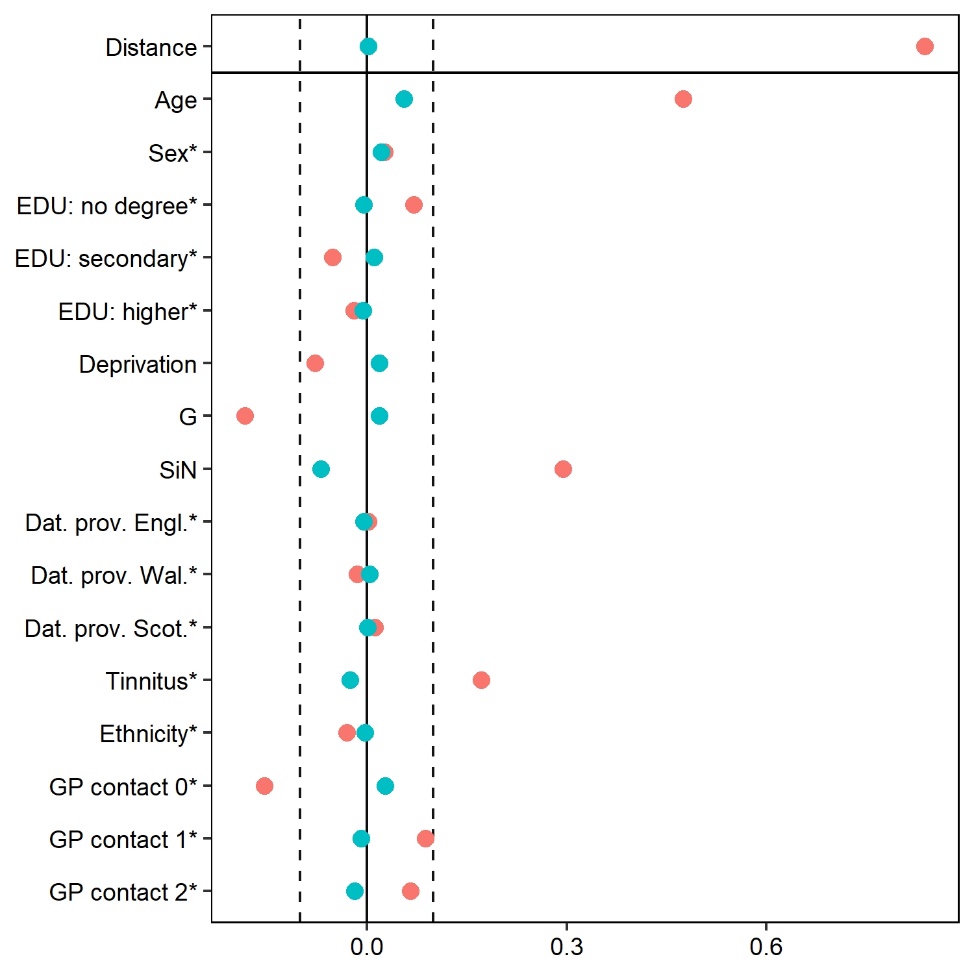

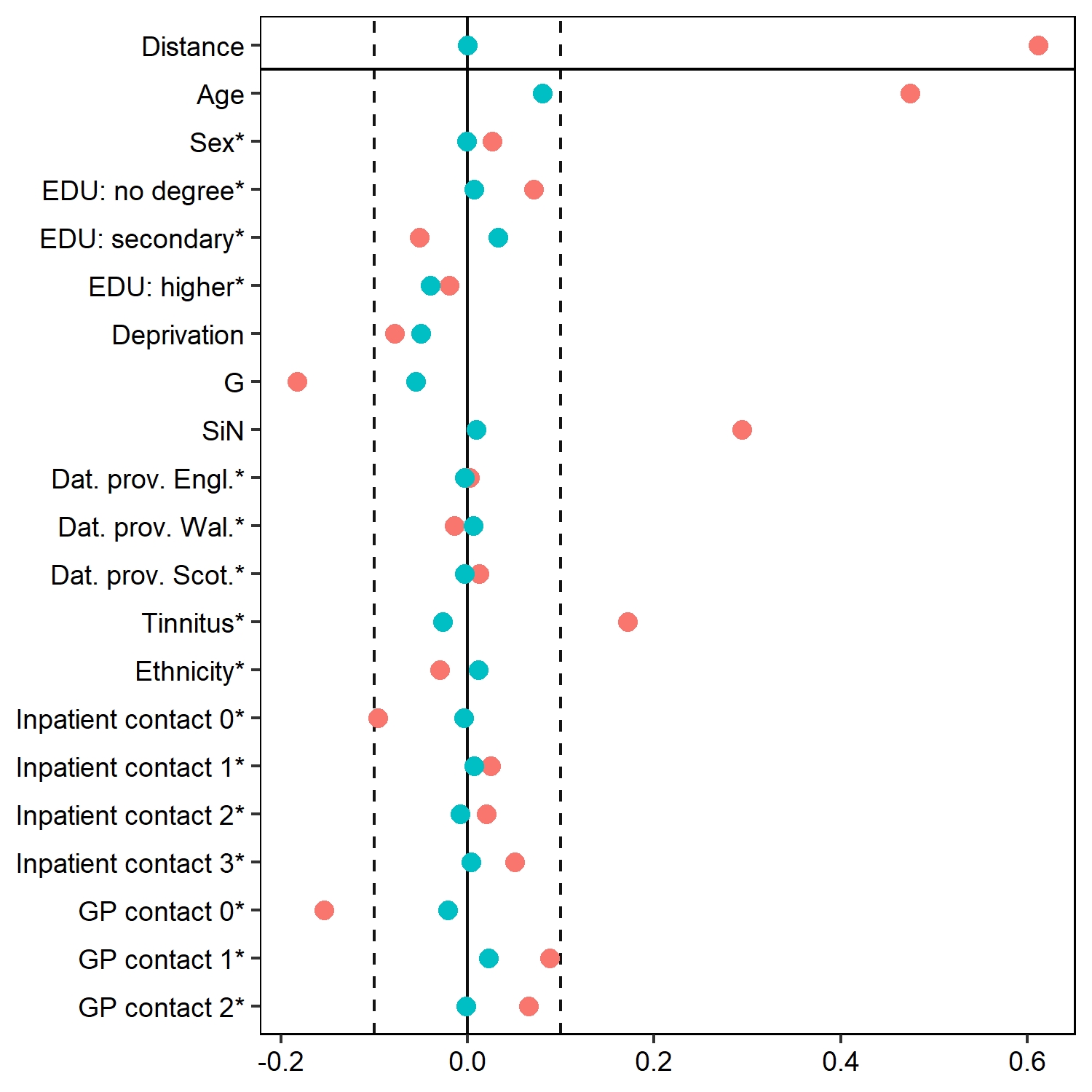


**10**

**11**


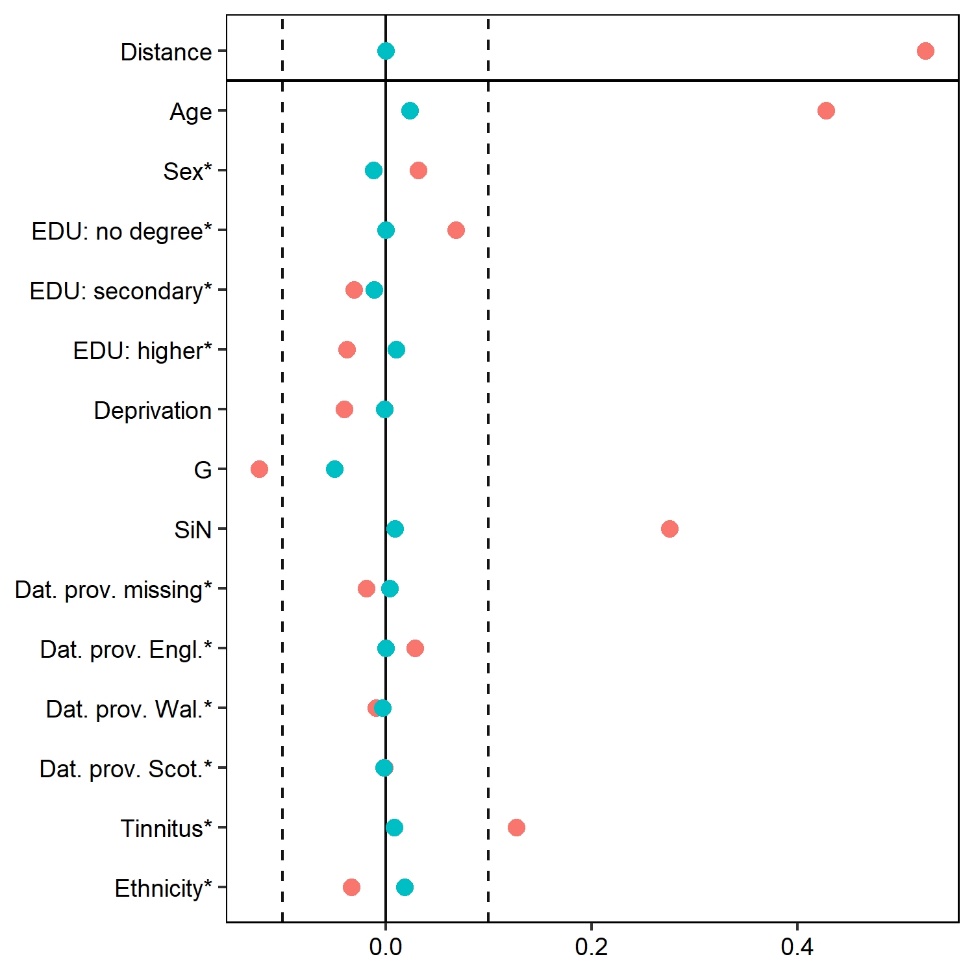

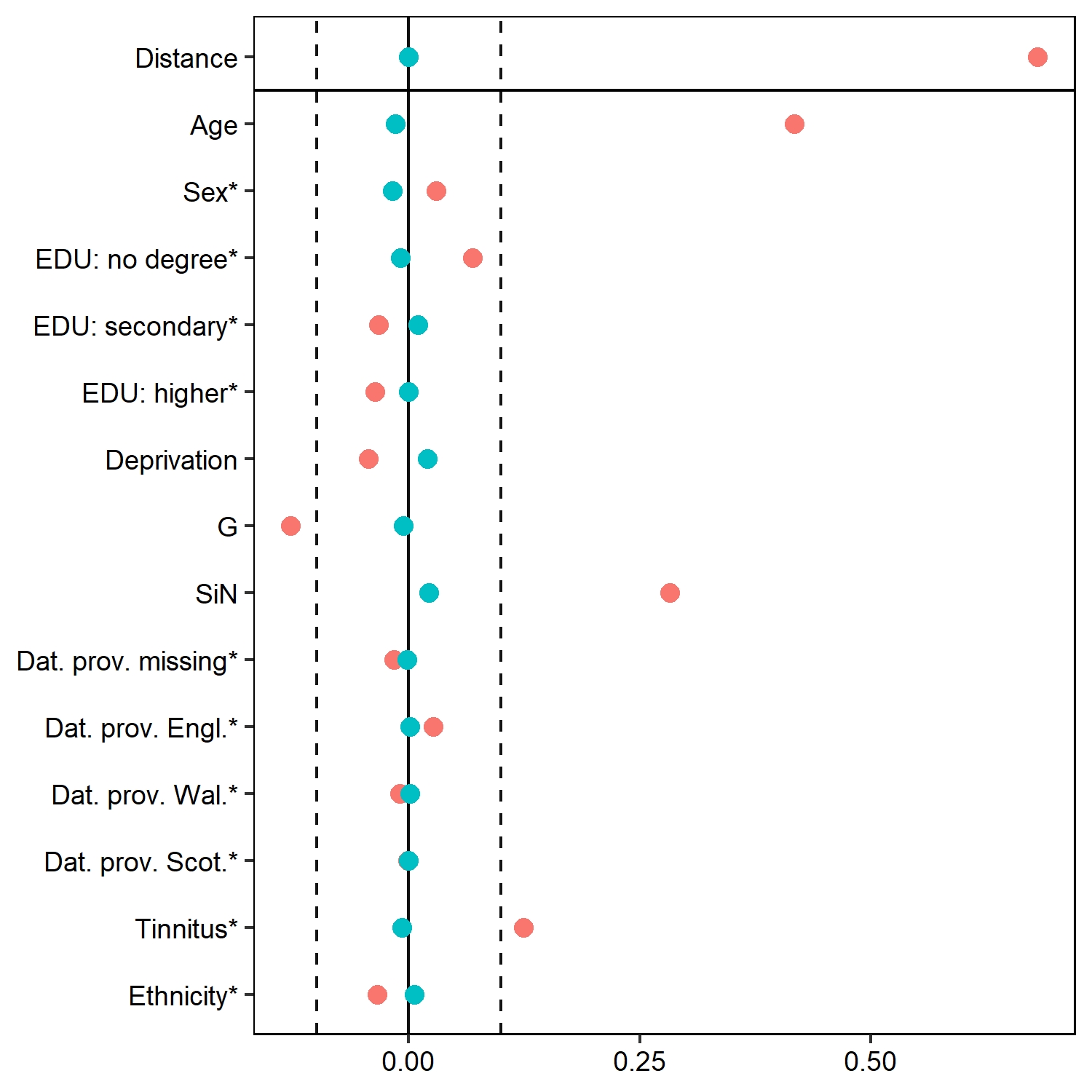


**12**

**13**


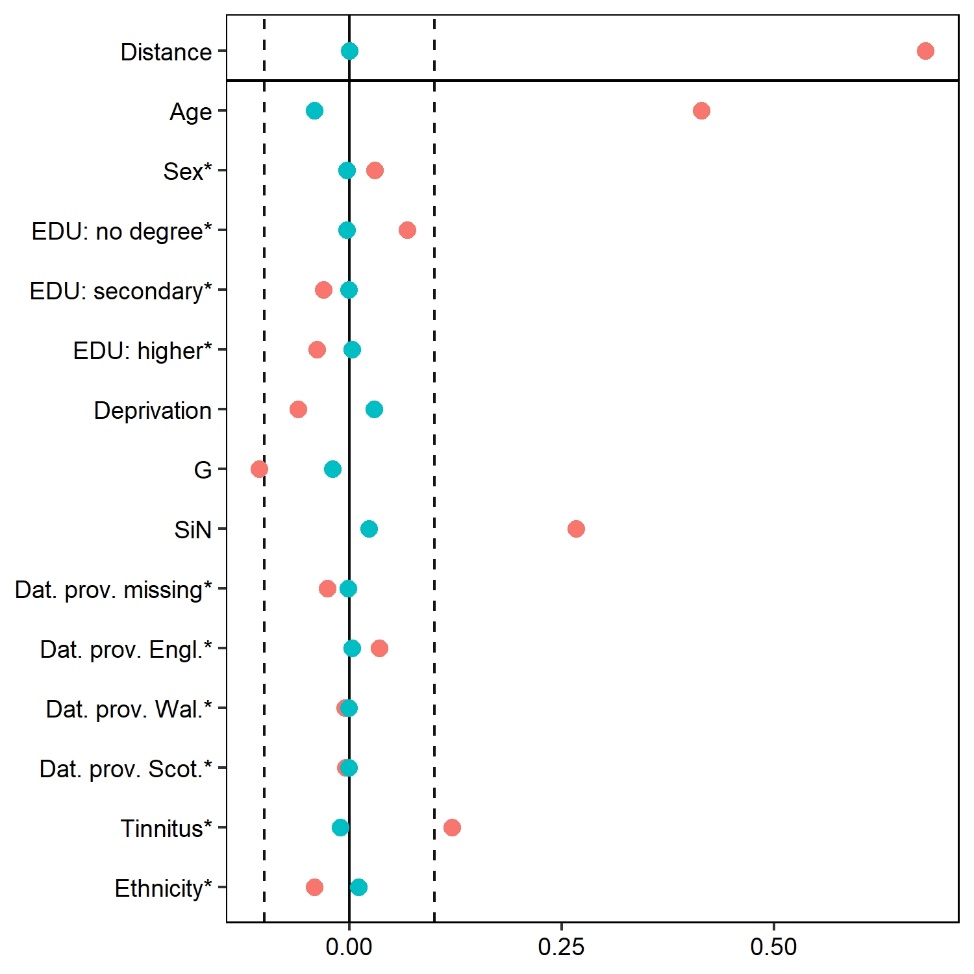

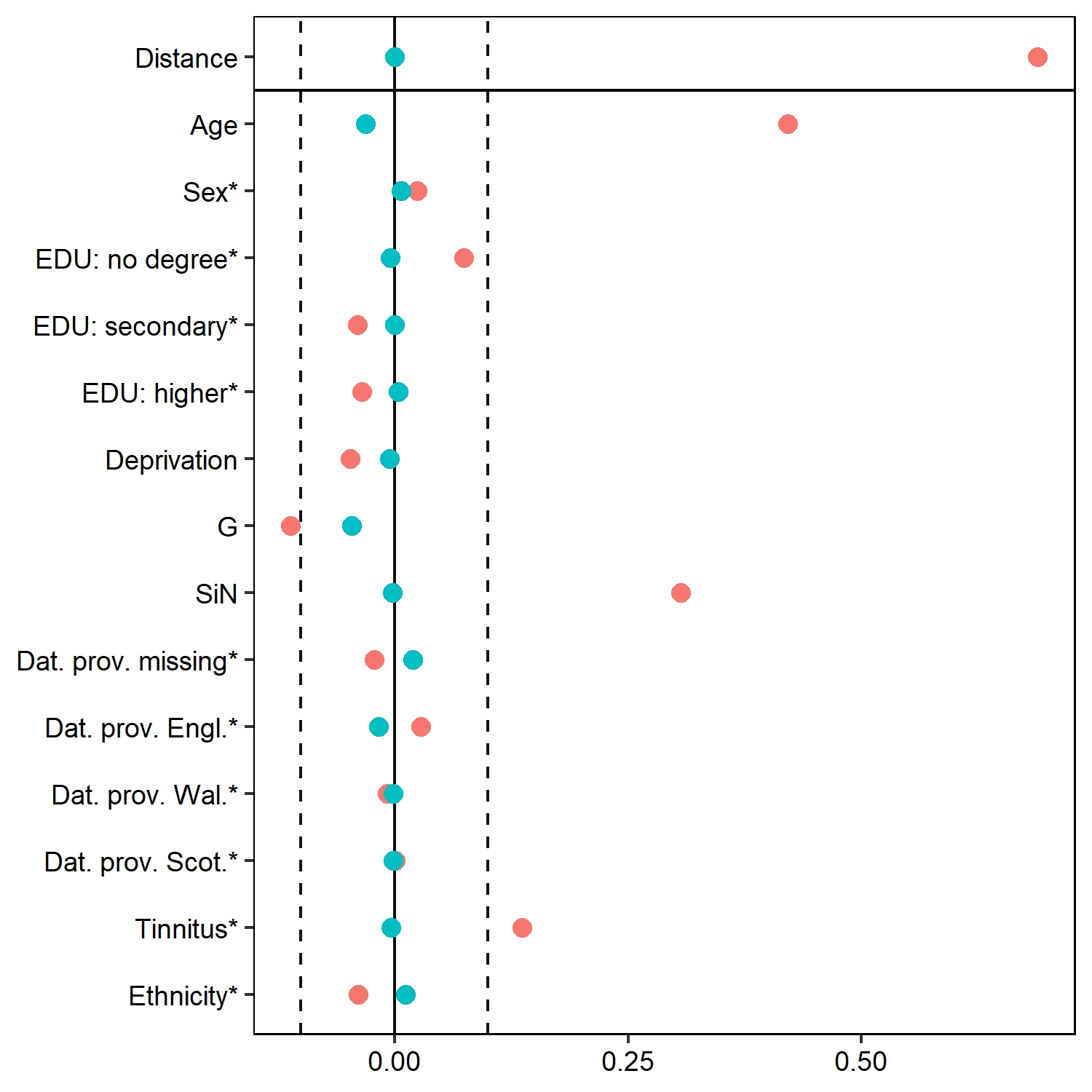


**16**

**17**


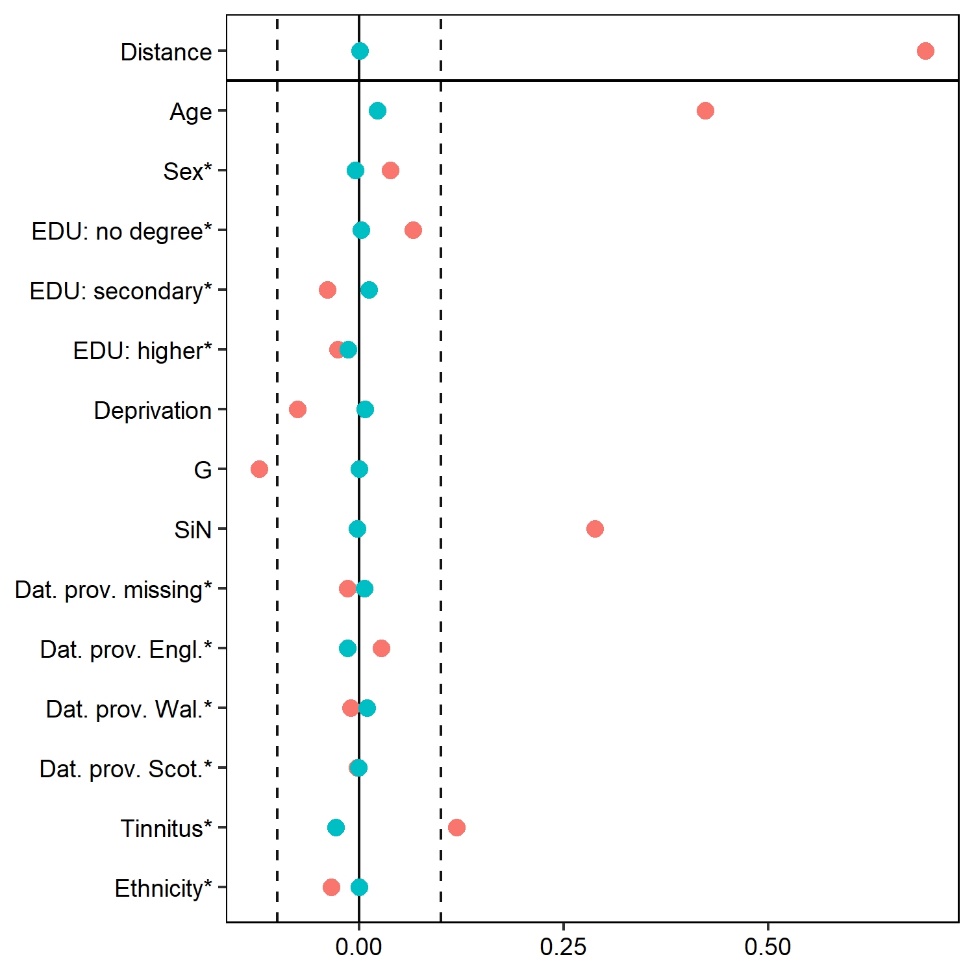

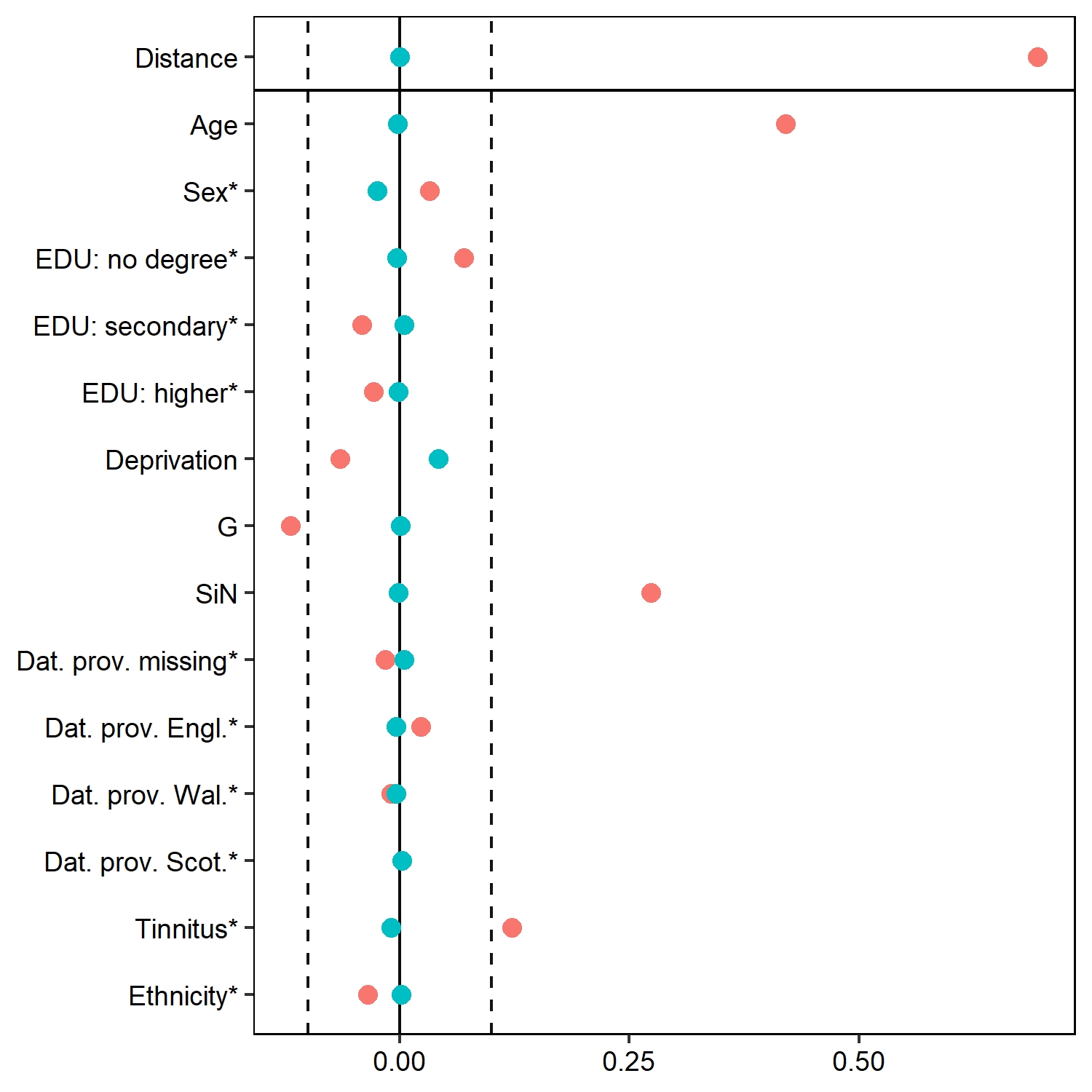


**14**

**15**


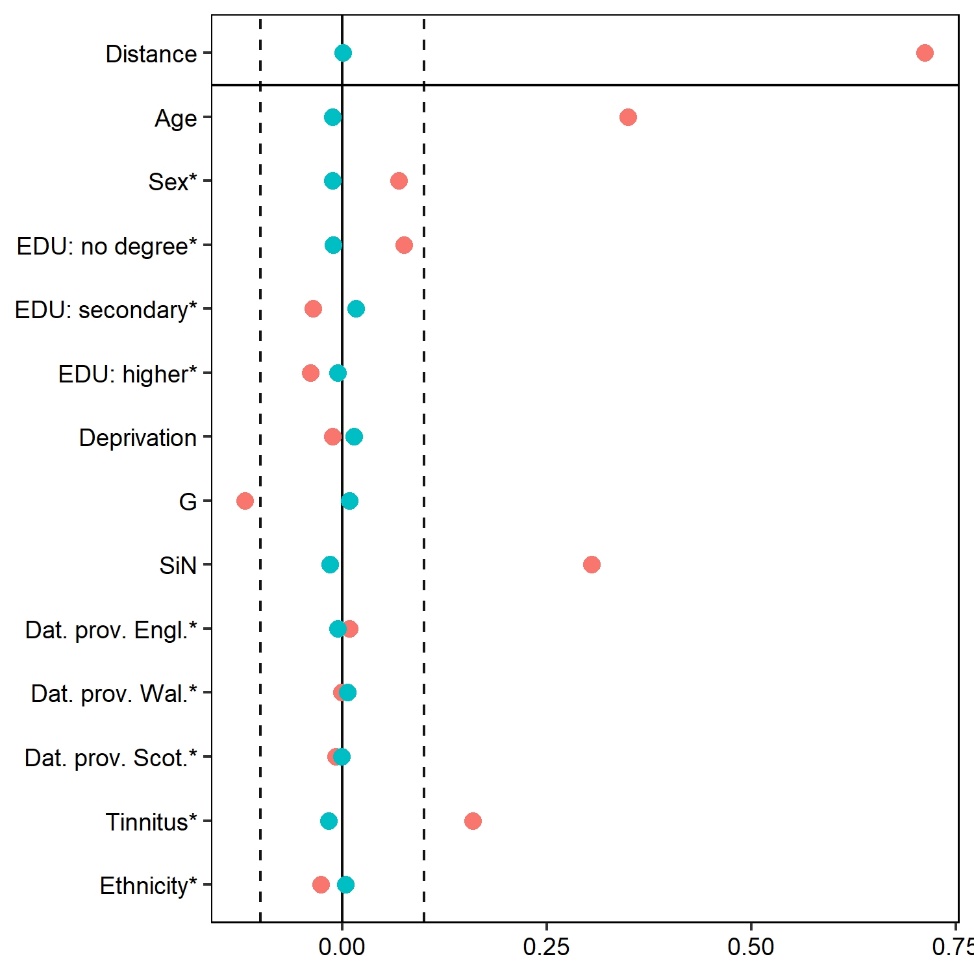

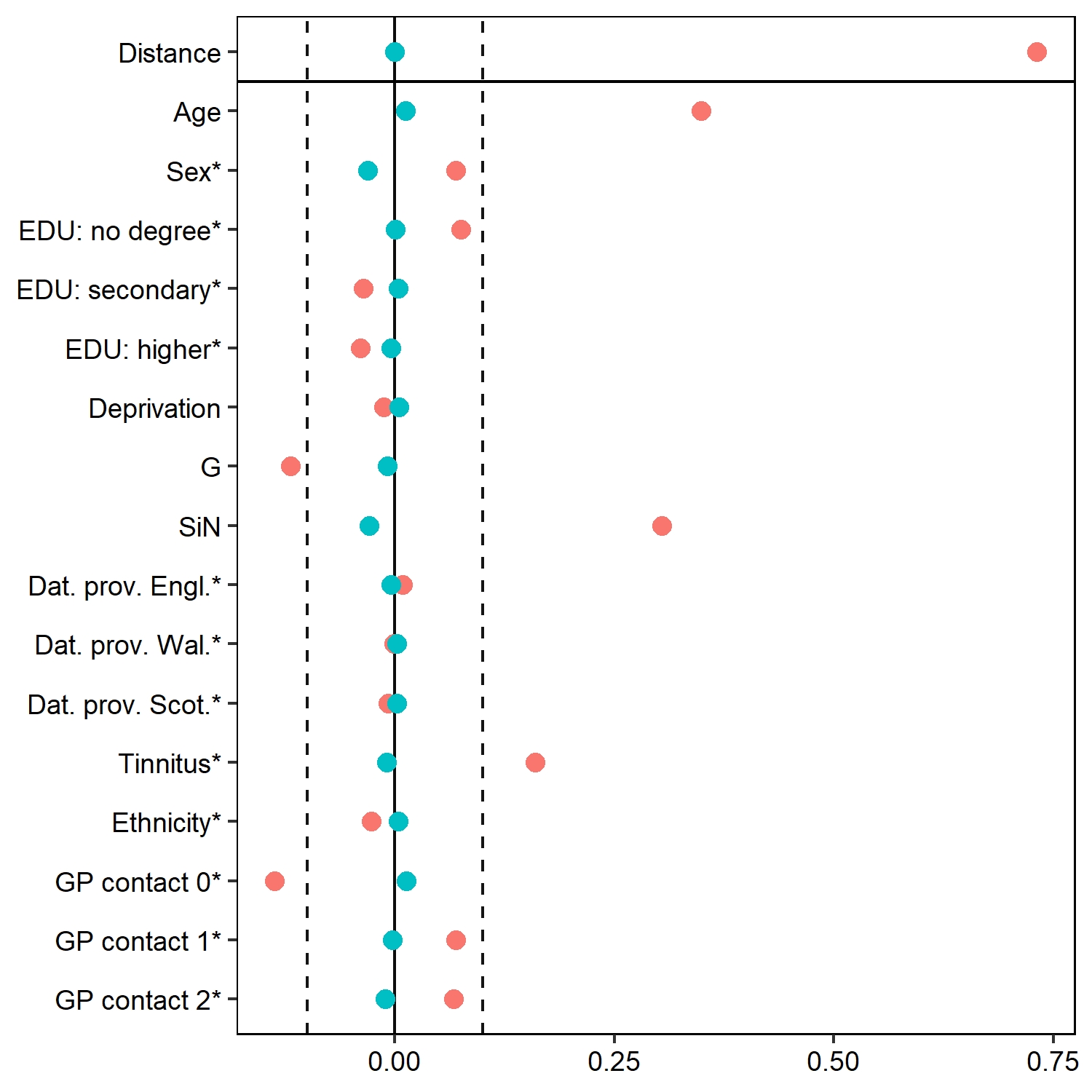


**21.2**

**21.3**


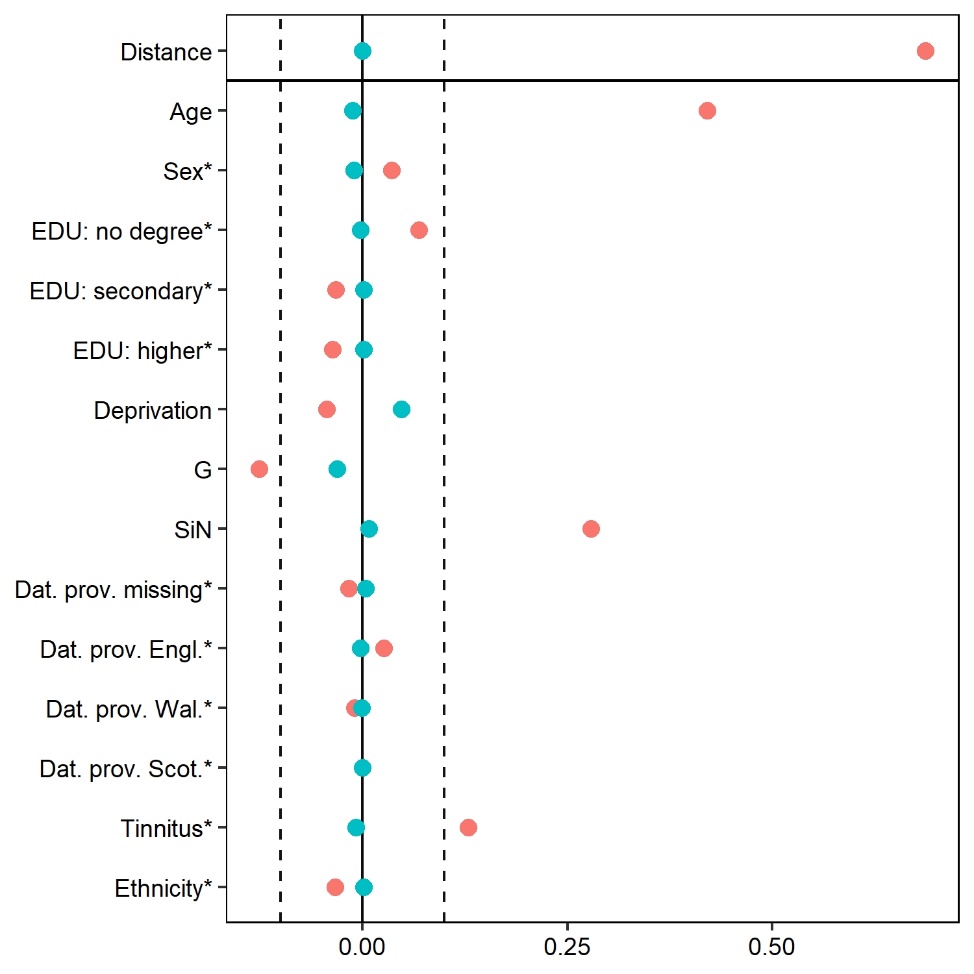

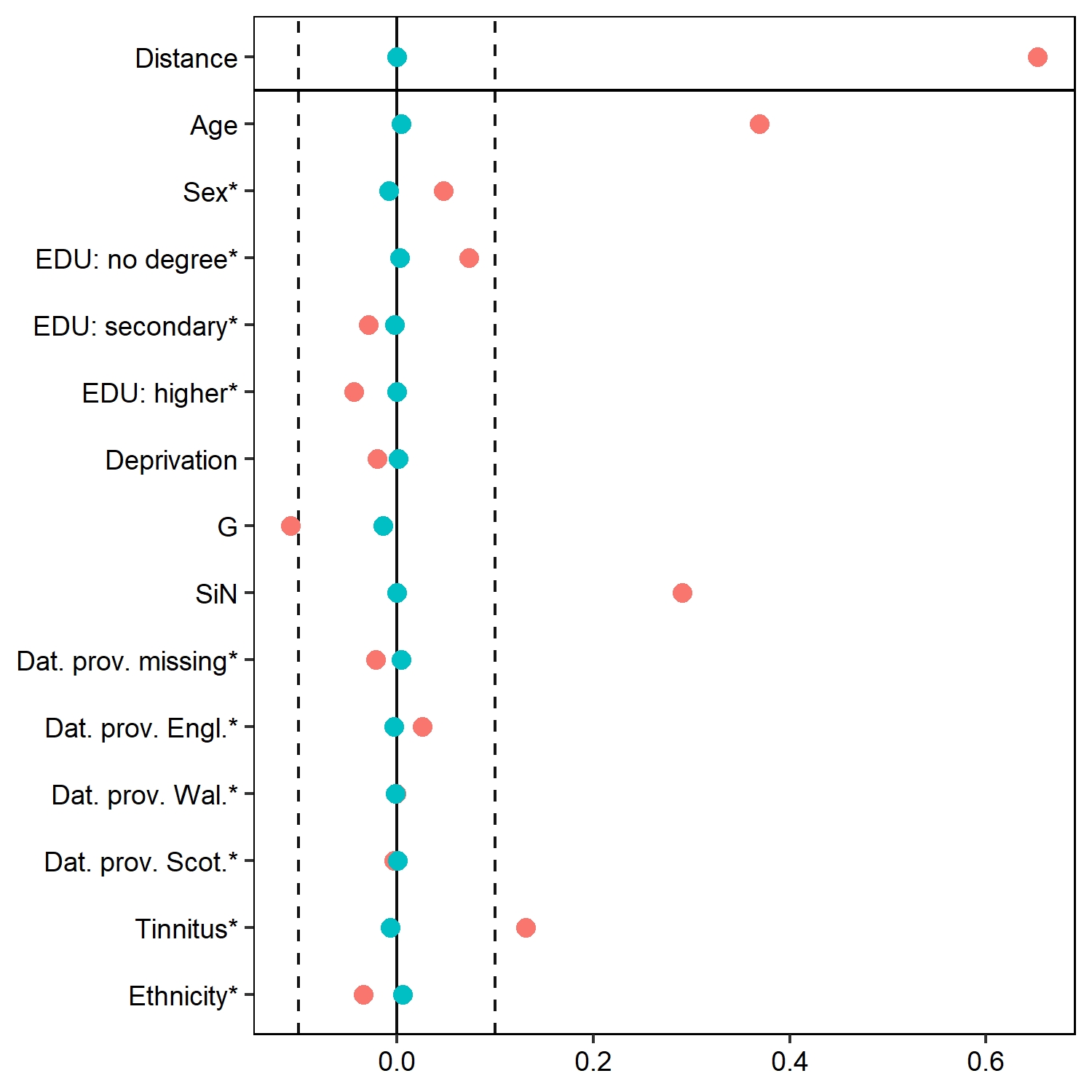


**20**

**21.1**


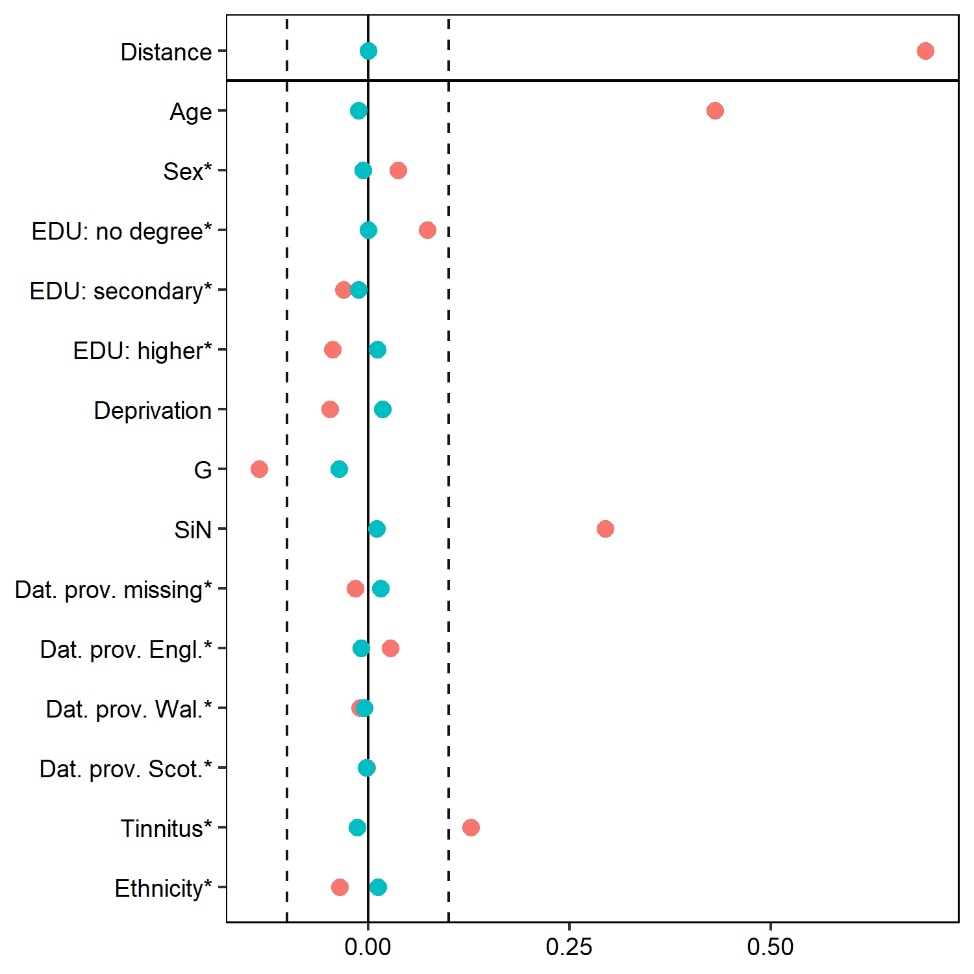

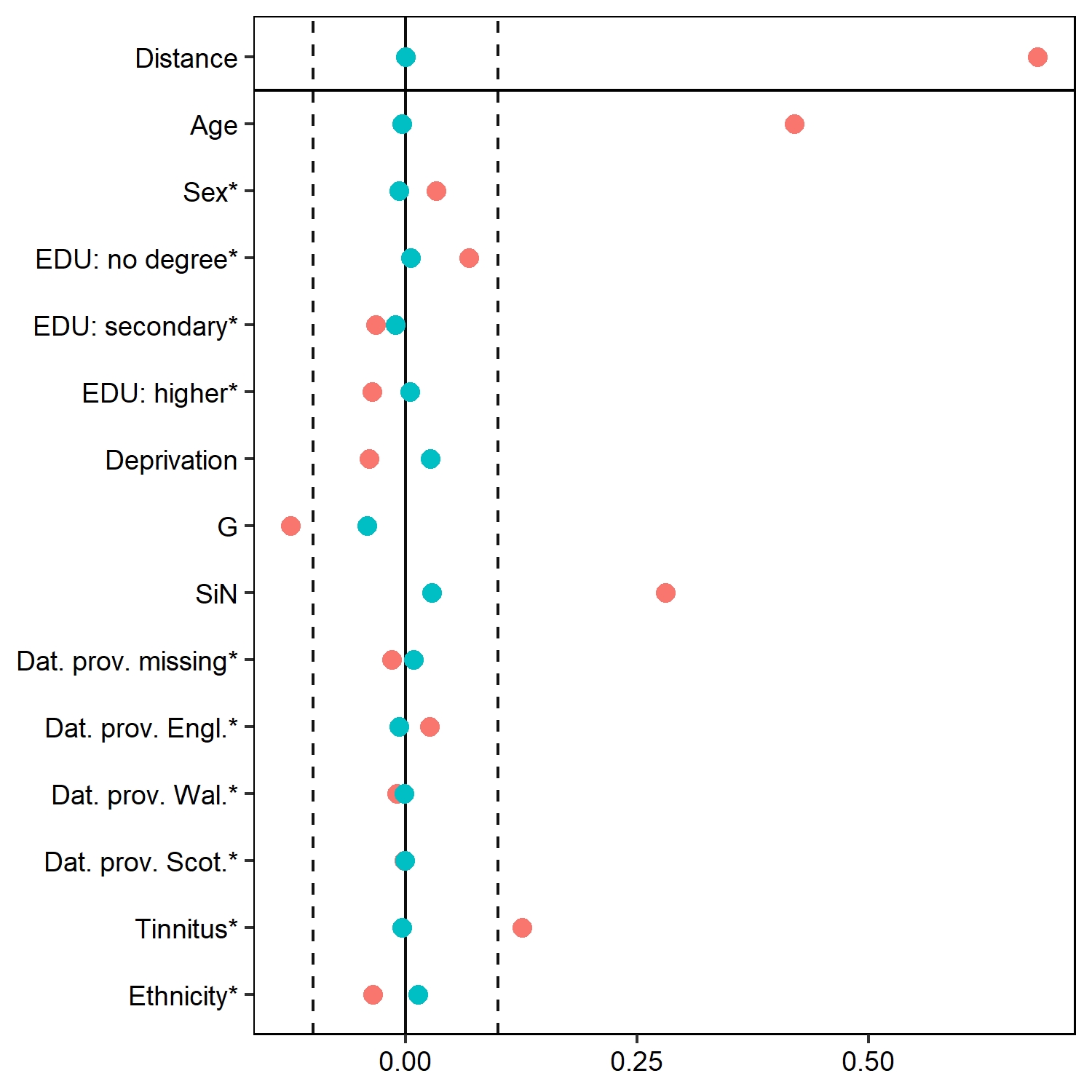


**18**

**19**


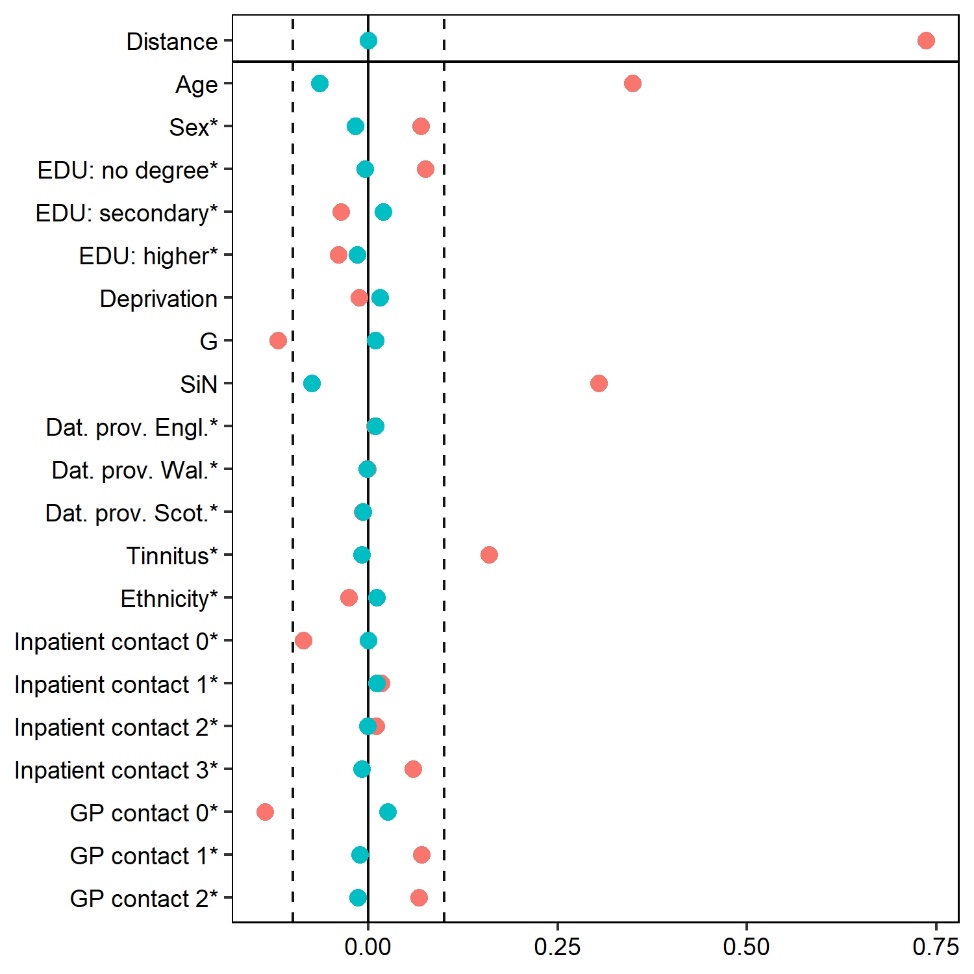


**21.4**

**Table S6:** the size of the analytical samples, numbers of HA users, numbers of participants diagnosed with the outcome, and the results of each intention-to-treat model. The numbers refer to (1) the basic model: intention-to-treat model for dementia, (2) per-protocol model for dementia, (3) basic model where participants with censoring during the grace period were removed prior to analysis, (4) basic model where only confounders measured before time zero were included in the analysis, (5) basic model where missing observation were imputed as opposed to removed (6) basic model with the addition of social isolation and the history of mood disorders as covariates, (7) basic model additionally adjusted for the history of head injury, (8) basic model additionally adjusted for the source of HL, (9) basic model additionally adjusted for the proxy for secondary hospital care utilisation, (10) basic model additionally adjusted for the proxy for primary care utilisation, (11) basic model additionally adjusted for proxies for secondary and primary healthcare utilisation, (12) model for influenza, (13) model for liver disease, (14) model for diseases of the lower respiratory tract, (15) model for asthma, (16) model for skin and subcutaneous diseases, (17) model for infectious and parasitic diseases, (18) model for appendicitis, (19) model for hip fractures, (20) model for transport accidents, (21) models without removal of pre-baseline HL cases. We used generalised full matching with covariate balancing propensity scores (CBPS) in models (5), (11), and (21.2), generalised additive models (GAM) ^23^ in model (12) and Bayesian additive regression trees (BART) in all others. N – sample size; HA – hearing aid; RR – risk ratio; HR – hazards ratio; ITT – intention-to-treat; PP – per protocol.

| **#** | **Model** | **N** | **N HA** | **N outcome** | **RR (95% CI)** | **HR (95% CI)** |
| --- | --- | --- | --- | --- | --- | --- |
| 1.1 | Basic ITT entire sample | 59,768 | 4,049 | 971 | 1.43 (1.08-1.88) | 1.31 (0.99-1.74) |
| 1.2 | Basic ITT GP subsample | 21,529 | 896 | 333 | 1.22 (0.67-2.17) | 1.14 (0.63-2.07) |
| 2 | Basic PP | 59,768 | 4,049 | 935 | 1.48 (1.12-1.95) | 1.35 (1.02-1.80) |
| 3 | Remove IDs in grace | 56,254 | 3,810 | 965 | 1.38 (1.07-1.79) | 1.20 (0.93-1.57) |
| 4 | Only prior confounders | 51,090 | 3,589 | 921 | 1.54 (1.21-1.96) | 1.36 (1.07-1.73) |
| 5 | Impute missing | 64,693 | 4,731 | 1,102 | 1.50 (1.23-1.82) | 1.30 (0.96-1.74) |
| 6 | Mood dis. & soc. isol. | 59,736 | 4,045 | 969 | 1.59 (1.12-2.28) | 1.44 (0.98-2.1) |
| 7 | Head injury | 59,768 | 4,049 | 971 | 1.34 (1.03-1.75) | 1.19 (0.9-1.58) |
| 8 | Source of HL | 59,768 | 4,049 | 971 | 1.41 (1.10-1.81) | 1.35 (1.04-1.74) |
| 9 | Inpatient contact | 59,768 | 4,049 | 971 | 1.26 (0.97-1.63) | 1.11 (0.85-1.44) |
| 10 | Primary care contact | 21,529 | 896 | 333 | 0.77 (0.44-1.33) | 0.57 (0.32-1.04) |
| 11 | All healthcare contact | 21,529 | 896 | 333 | 0.68 (0.39-1.18) | 0.63 (0.37-1.09) |
| 12 | Influenza | 56,810 | 3,894 | 3,571 | 1.39 (1.20-1.61) | 1.25 (1.07-1.47) |
| 13 | Liver disease | 58,995 | 4,001 | 1,754 | 1.26 (0.99-1.61) | 1.2 (0.93-1.54) |
| 14 | Respiratory disease | 50,105 | 3,336 | 2,942 | 1.14 (0.95-1.37) | 1.06 (0.88-1.29) |
| 15 | Asthma | 52,260 | 3,498 | 1,384 | 1.12 (0.8-1.57) | 1.10 (0.72-1.69) |
| 16 | Skin disorders | 40,919 | 3,019 | 6,347 | 1.16 (1.01-1.32) | 1.12 (0.94-1.34) |
| 17 | Infections | 41,591 | 3,051 | 6,668 | 1.12 (1.00-1.32) | 1.05 (0.91-1.20) |
| 18 | Appendicitis | 57,570 | 3,892 | 274 | 0.60 (0.29-1.25) | 0.55 (0.27-1.13) |
| 19 | Hip fractures | 59,682 | 4,048 | 623 | 1.28 (0.91-1.80) | 1.20 (0.85-1.69) |
| 20 | Transport accidents | 59,208 | 4,010 | 600 | 0.99 (0.66-1.47) | 1.24 (0.93-1.39) |
| 21.1 | Basic ITT entire sample | 66,051 | 5,624 | 1,145 | 1.41 (1.15-1.72) | 1.28 (1.04-1.56) |
| 21.2 | Basic ITT GP subsample | 27,313 | 2,241 | 491 | 1.49 (1.06-2.09) | 1.24 (0.88-1.74) |
| 21.3 | Primary care contact | 27,313 | 2,241 | 491 | 1.30 (0.95-1.78) | 1.05 (0.75-1.47) |
| 21.4 | All healthcare contact | 27,313 | 2,241 | 491 | 1.30 (0.94-1.78) | 1.05 (0.75-1.46) |

**Figure S5** (see also next two pages): Kaplan-Meier survival curves for each Cox proportional hazards model in the study. The model numbers are the same as in **Tables S4 and S6**. The blue dotted line and red line are survival curves for HA users and non-users, respectively. The x-axis displays years of follow-up; the y-axis depicts the proportion of participants that have not experienced the outcome since time zero. Note that the range for the y-axis differs between the models. For the analysis using imputed data (5), the survival curve was generated by pooling the 10 Kaplan Meier survivals using Rubin’s rules after complementary log-log transformation^24,25^.


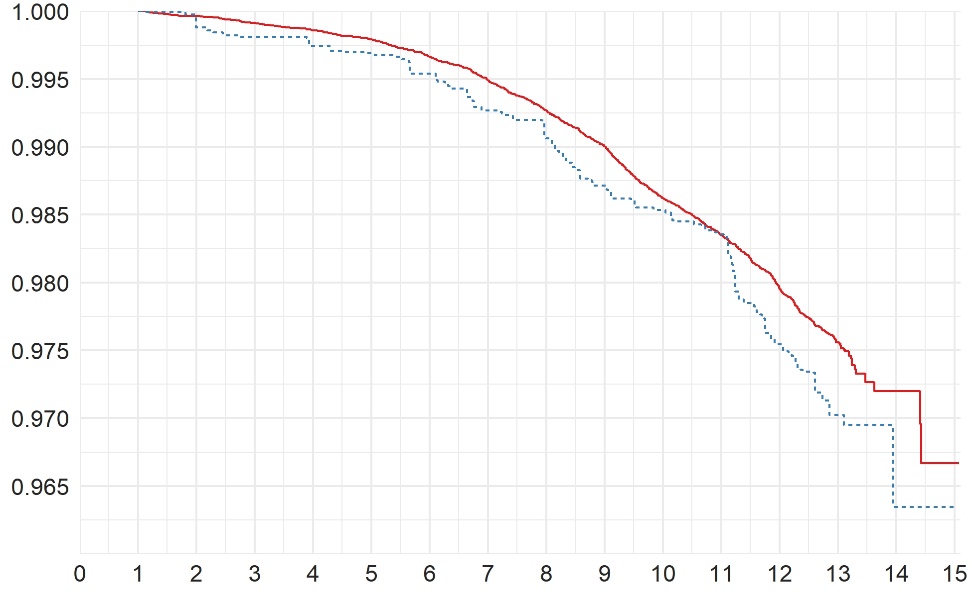

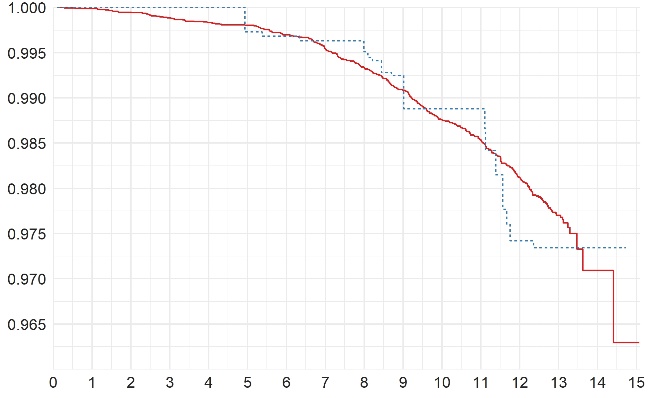


**1**

**1.1**


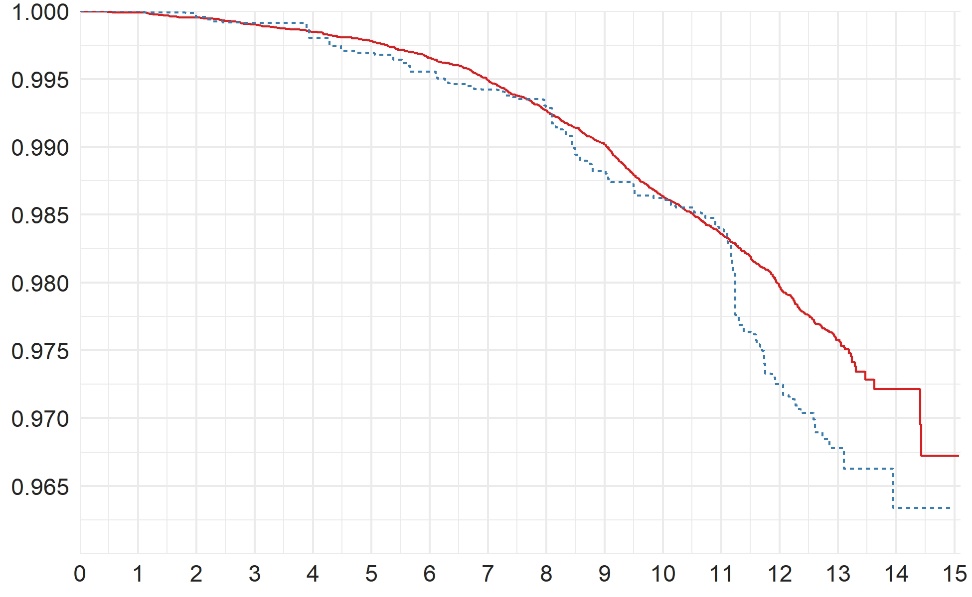

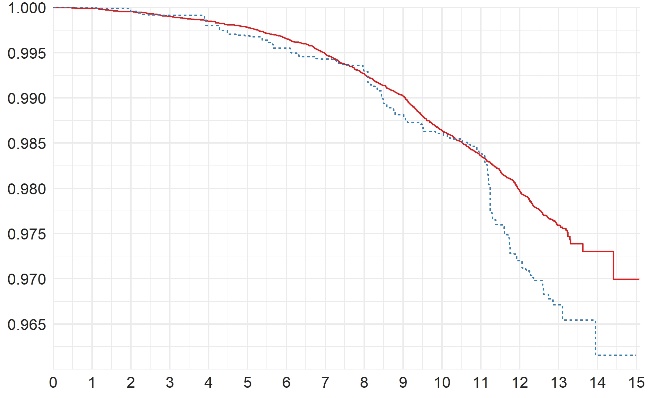


**2**

**3**


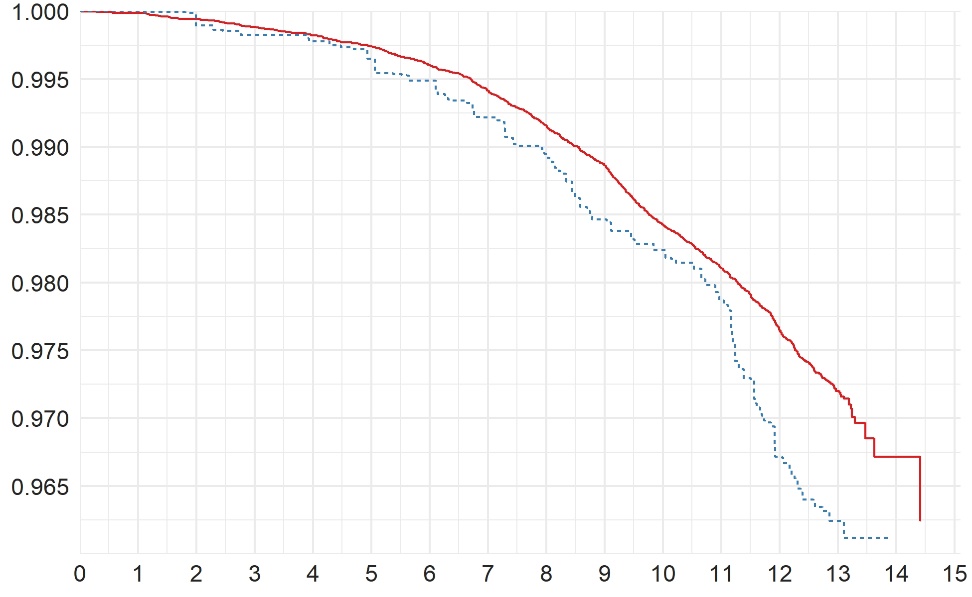

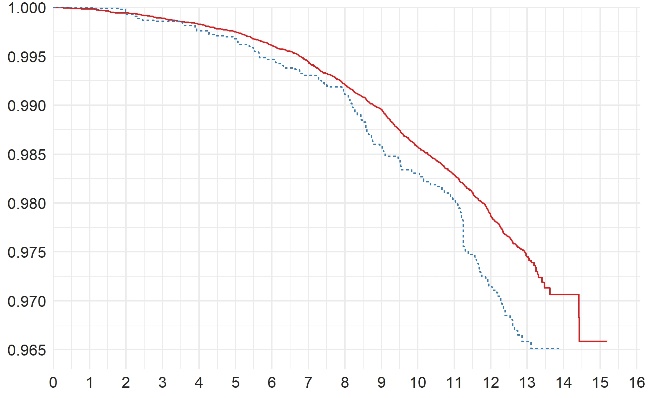


**4**

**5**


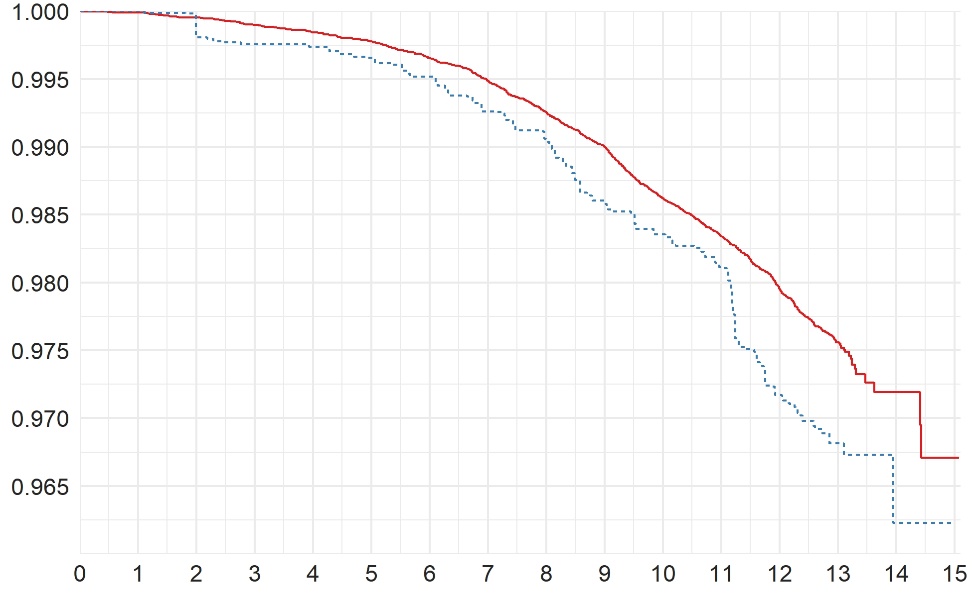

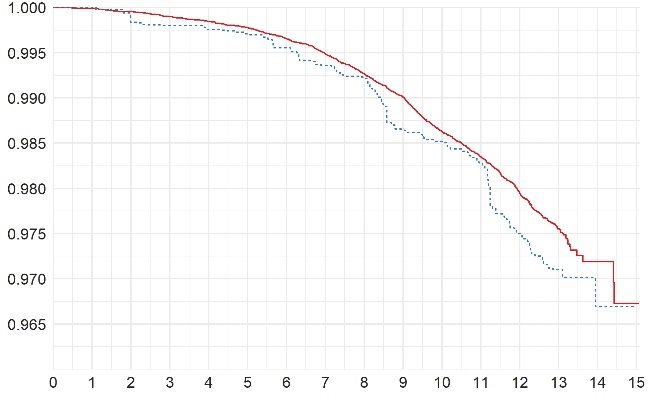


**6**

**7**


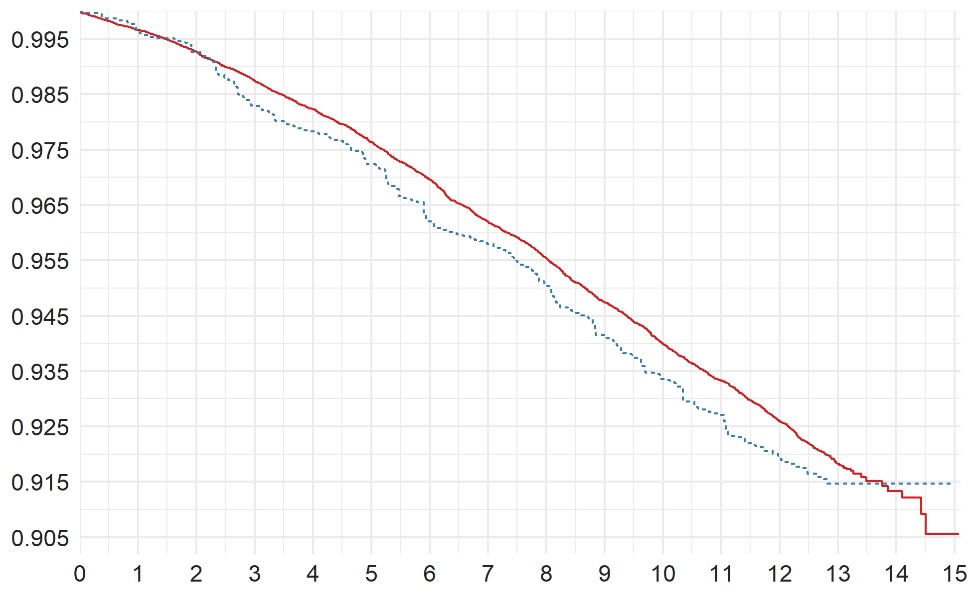

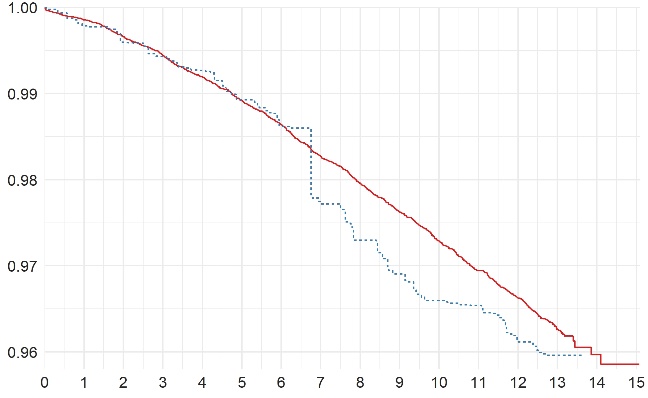


**14**

**15**

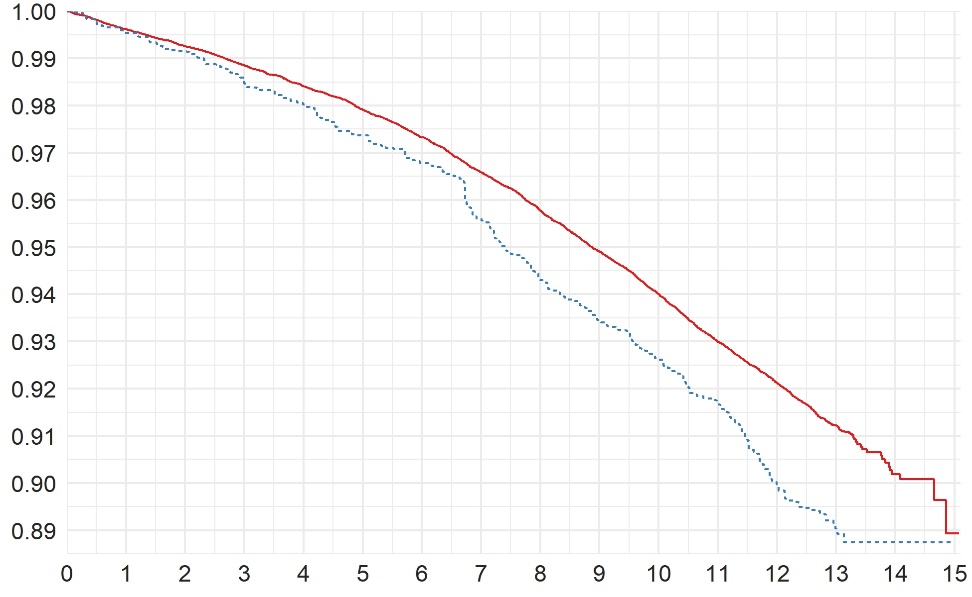

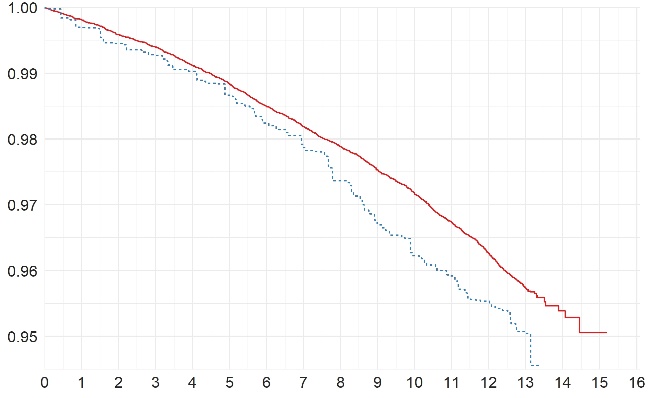


**12**

**13**

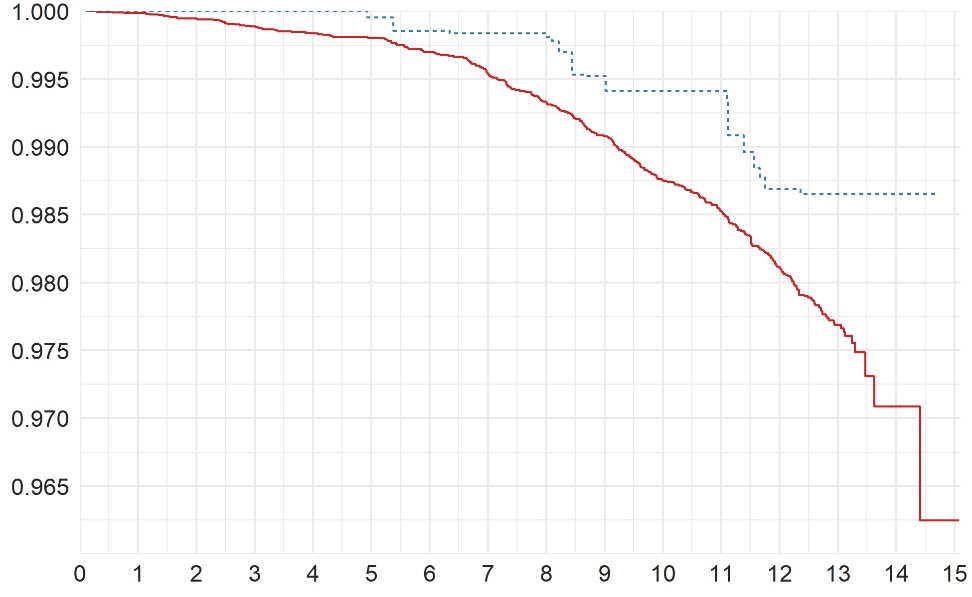

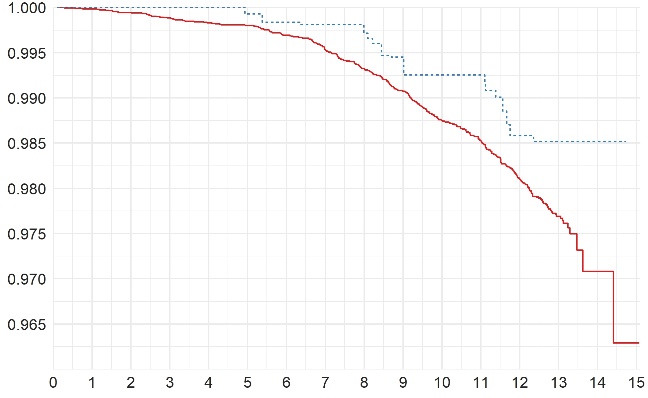


**10**

**11**


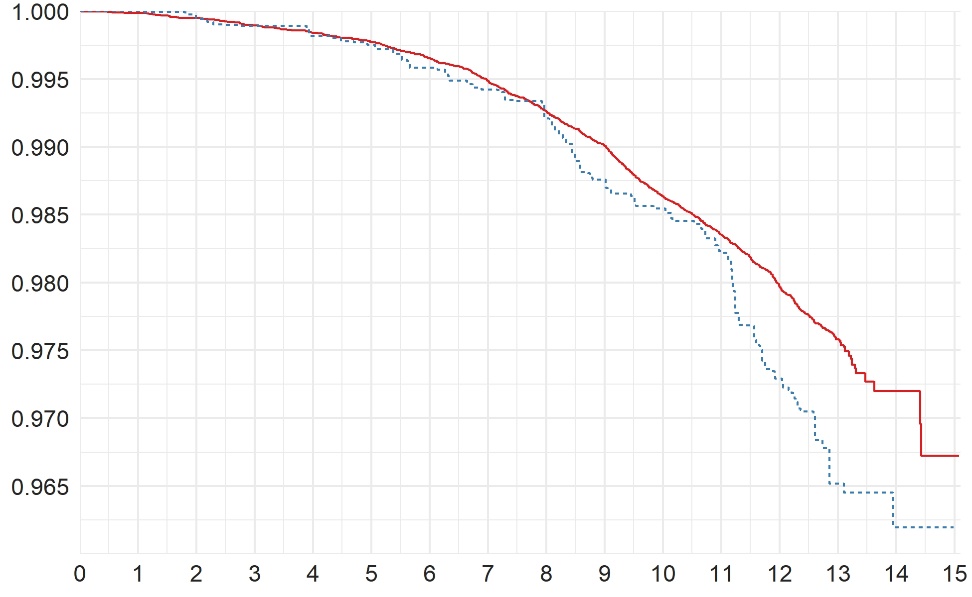

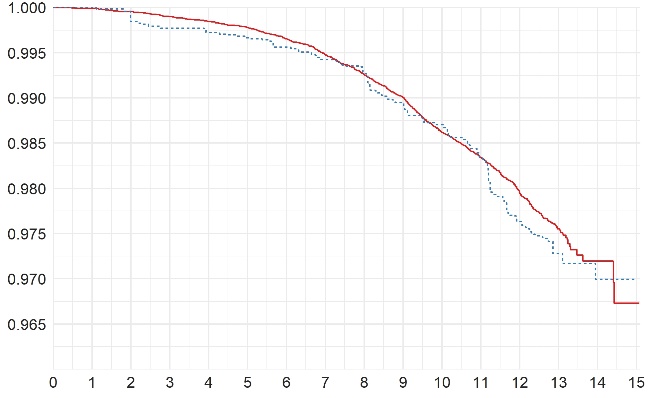


**8**

**9**


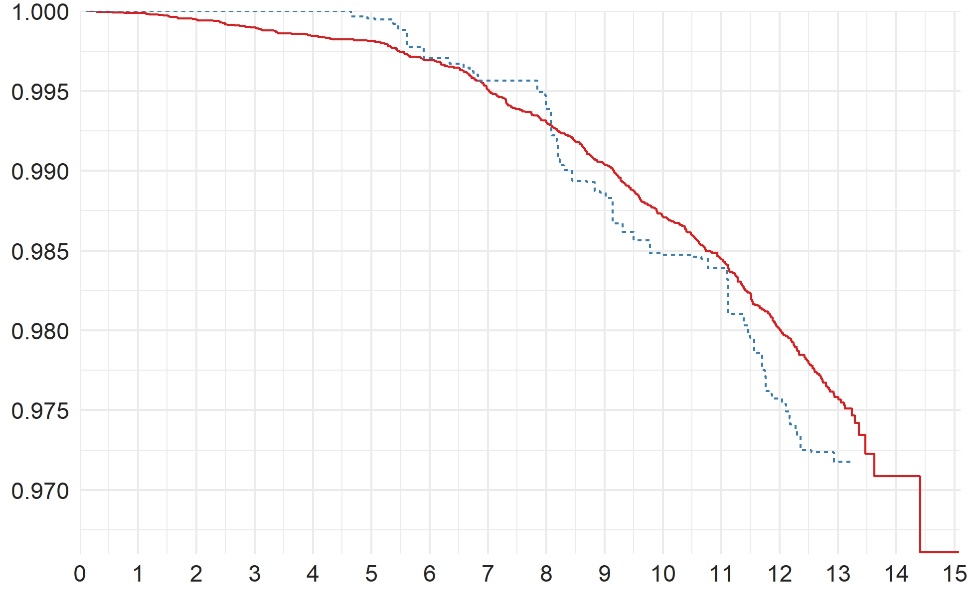

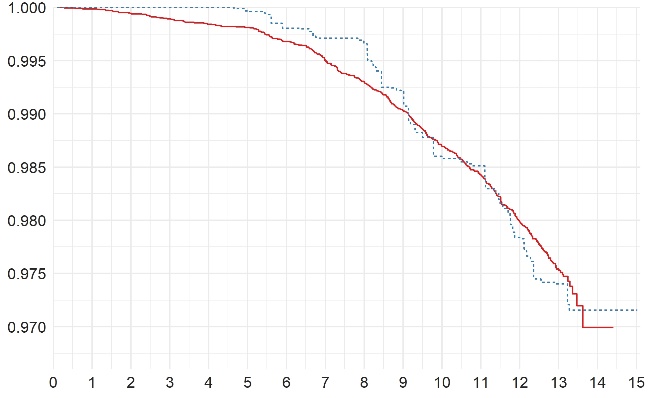


**21.2**

**21.3**

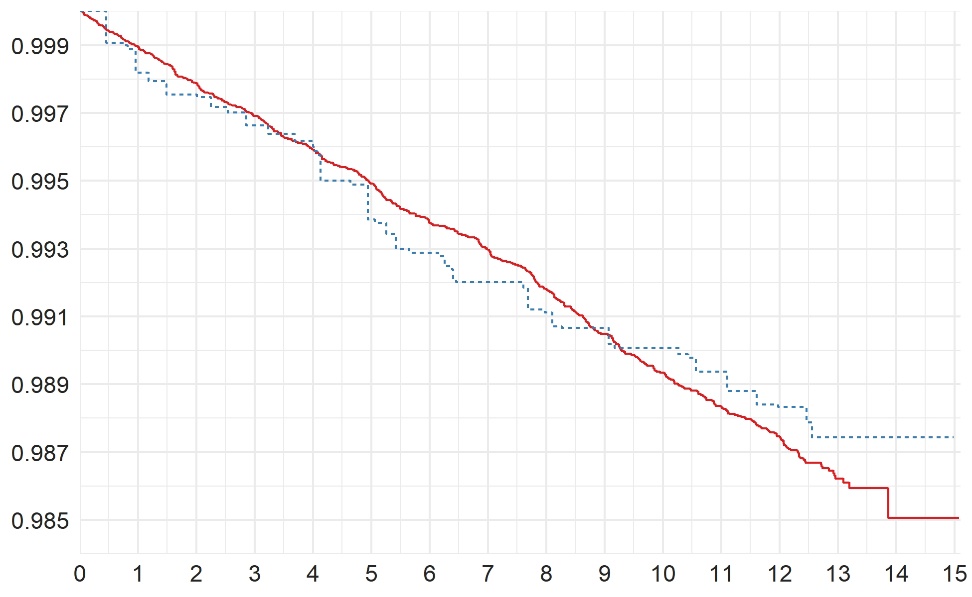

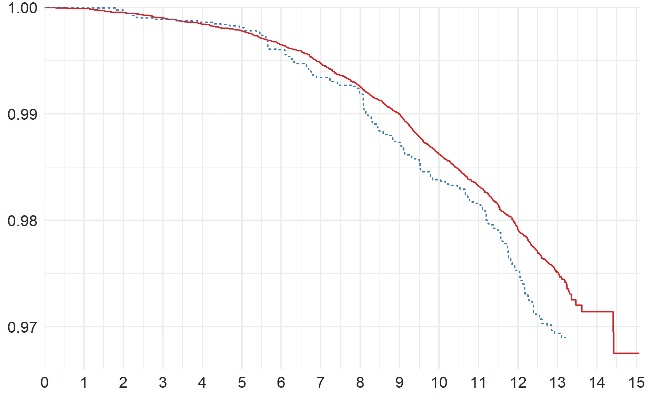


**20**

**21.1**

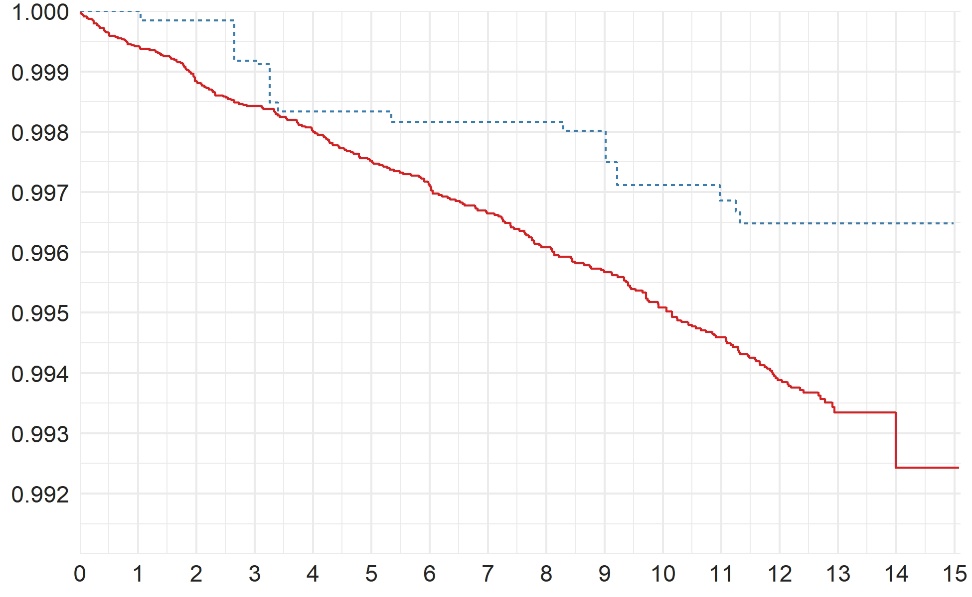

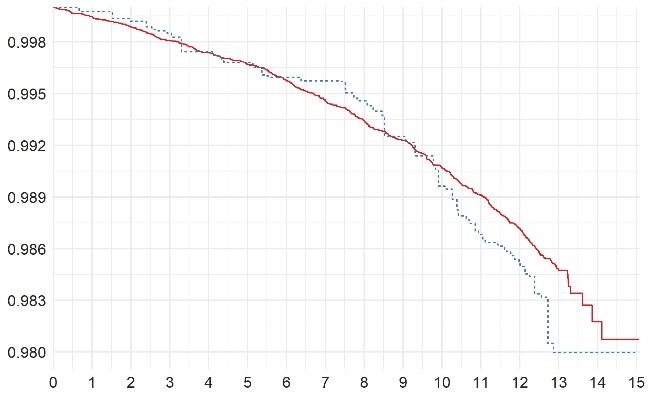


**18**

**19**

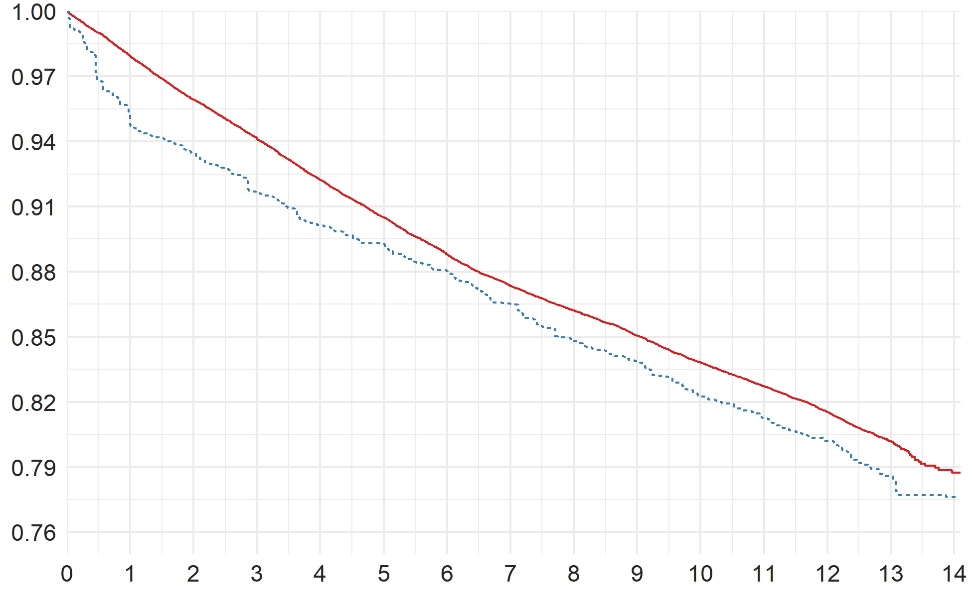

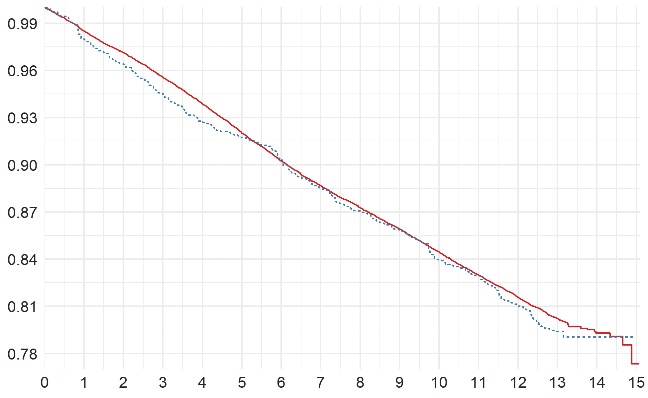


**16**

**17**

**21.4**

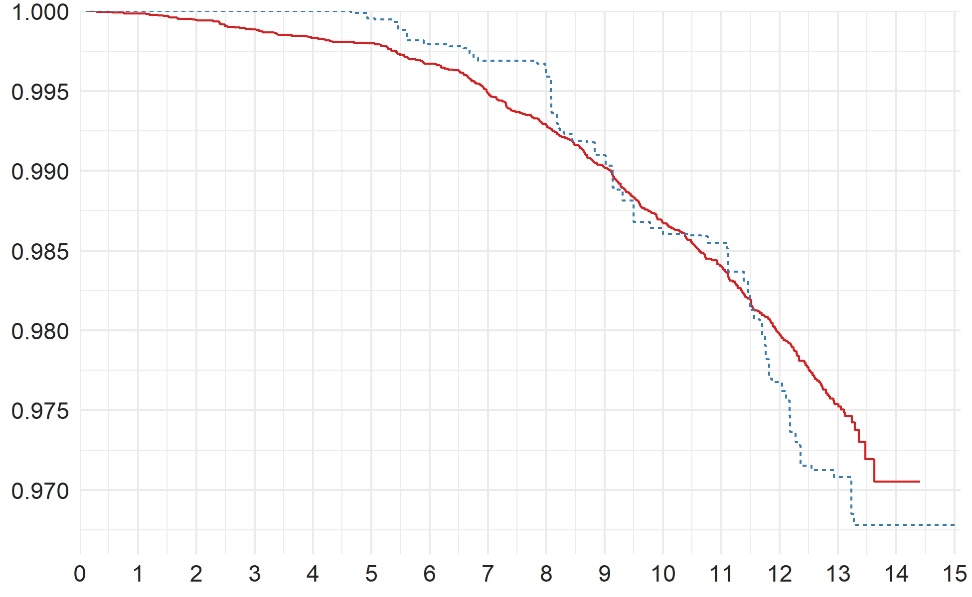


**Figure S6**: additional analyses on healthcare contact. **A**: the effect of HA on the extent of post-randomisation healthcare contact. **B**: the effect of pre-randomisation healthcare contact on the risk of post-randomisation diagnosis of dementia. For primary care contact, categories 1 and 2 refer to >12 & ≤ 24 and >24 visits per annum, respectively (reference: ≥0 & ≤12); for inpatient healthcare contact, categories 1, 2, and 3 refer to >0 & ≤ 0.2, >0.2 & ≤0.5, and >0.5 spells per annum, respectively (reference: 0). Greater pre-randomisation healthcare utilisation was associated with a higher risk dementia diagnosis for both primary care (RR=1.53 (CI=1.17-2.0) for > 12 & ≤ 24, RR=2.78 (CI=2.07-3.73) for > 24) and inpatient hospital care (RR=1.08 (CI=0.92-1.27) for > 0 & ≤ 0.2, RR=1.29 (CI=1.06-1.56) for > 0.2 & ≤ 0.5, RR=1.58 (CI=1.33-1.43) for > 0.5); HA use was associated with greater post-randomisation healthcare utilisation for both primary care (RR=1.19 (CI=1.06-1.35) for > 12 & ≤ 24, RR=1.22 (CI=1.08-1.37) for > 24) and inpatient hospital care (RR=1.19 (CI=1.11-1.28) for > 0 & ≤ 0.2, RR=1.19 (CI=1.13-1.26) for > 0.2 & ≤ 0.5, RR=1.29 (CI=1.22-1.36) for > 0.5).


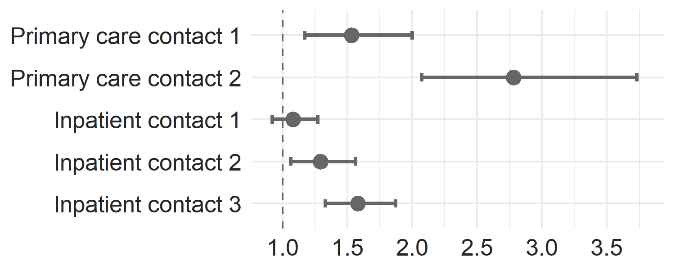

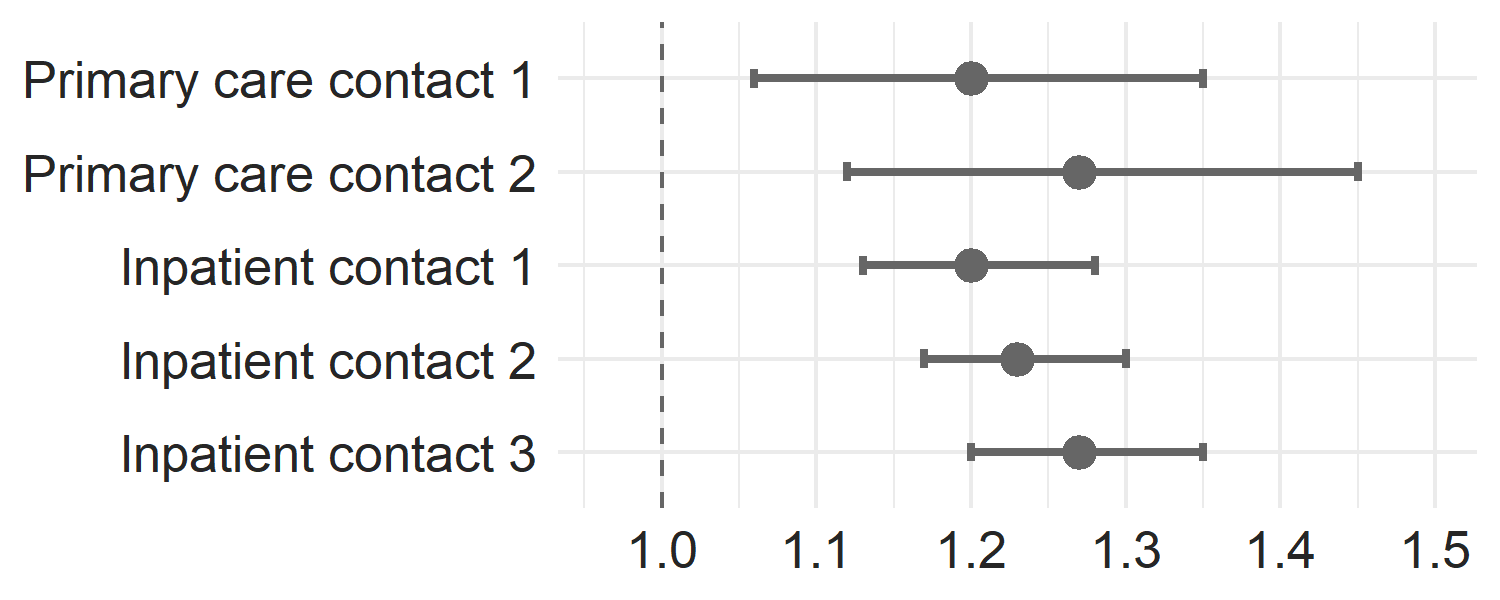


**Table S7:** differences between the analytical subsamples of participants with and without primary-care data. The three columns of the table depict the same information as **Table 2** in the main text, but separately for the subsample with primary-care data, the subsample without primary-care data, and the entire sample.

|  | **Primary-care subsample**  N (%) | **Subsample without primary care**  N (%) | **Entire sample** N (%) |
| --- | --- | --- | --- |
|  |  |  |  |
| Age, median (IQR) | 62.5 (10.8) | 63.1 (10.4) | 62.9 (10.5) |
| Female sex | 10,643 (49.4) | 18,831 (49.2) | 29,474 (49.3) |
| Education |  |  |  |
| *No degree* | 3,496 (16.2) | 5,781(15.1) | 9,274 (15.5) |
| *Secondary/vocational degree* | 7,112 (33.0) | 12,501(32.7) | 19,613 (32.8) |
| *Higher degree* | 10,924 (50.7) | 19,957 (52.2) | 30,881 (48.4) |
| Deprivation, median (IQR) | -2.14 (4.0) | -2.11 (4.0) | -2.13 (4.0) |
| White ethnicity | 20,437 (94.9) | 35,643 (93.2) | 56,080 (93.8) |
| G, median (IQR) | 0.003 (1.45) | -0.048 (1.49) | -0.030 (1.48) |
| SiN* median (IQR)  mean (SD) | -6 (2.5) -6.1 (2.3) | -6 (3) -5.8 (2.3) | -6 (3) -5.9 (2.3) |
| Data source |  |  |  |
| *England* | 19,827 (92.1) | 34,198 (89.4) | 54,025 (90.4) |
| *Scotland* | 684 (3.2) | 618 (1.6) | 1,302 (2.2) |
| *Wales* | 1,018 (4.7) | 245 (0.6) | 1,263 (2.1) |
| *Unknown* | 0 | 3,178 (8.3) | 3,178 (5.3) |
| Follow-up, median (IQR) | 12.4 (6.5) | 12.4 (8.4) | 12.4 (7.9) |
| History of tinnitus | 8,353 (38.8) | 15,622 (40.9) | 23,945 (40.1) |
| Social isolation | 10,015 (46.5) | 17,964 (47.0) | 27,979 (46.8) |
| History of mood disorders | 2,867 (13.3) | 3,580 (9.4) | 6,447 (10.8) |
| History of head injury | 178 (0.83) | 293 (0.77) | 471 (0.79) |
| Annual hospitalisations |  |  |  |
| *0* | 11,564 (53.7) | 20,147 (52.7) | 31,711 (53.1) |
| *> 0 & ≤ 0.2* | 5,104 (23.7) | 9,013 (23.6) | 14,117 (23.6) |
| *> 0.2 & ≤ 0.5* | 2,349 (10.9) | 4,372 (11.4) | 6,721 (11.2) |
| *> 0.5* | 2,512 (11.7) | 4,707 (12.3) | 7,219 (12.1) |
| Annual GP appointments |  |  |  |
| *≥ 0 & ≤ 12* | 11,576 (53.8) | NA | 11,576 (53.8) |
| *> 12 & ≤ 24* | 7,219 (33.5) | NA | 7,219 (33.5) |
| *> 24* | 2,734 (12.7) | NA | 2,734 (12.7) |

**Both the mean and the median are given; for other numerical variables, only the median is shown.*

**Figure S7:** results from analyses on the effect of HA use on dementia when participants with HL diagnosis prior to baseline were retained in the sample. “Basic” refers to the main intention-to-treat and per-protocol models for dementia, “sample change” refers to models where the analytical sample was altered, “extra adjust.” refers to those where additional covariates were used for matching and adjustment.


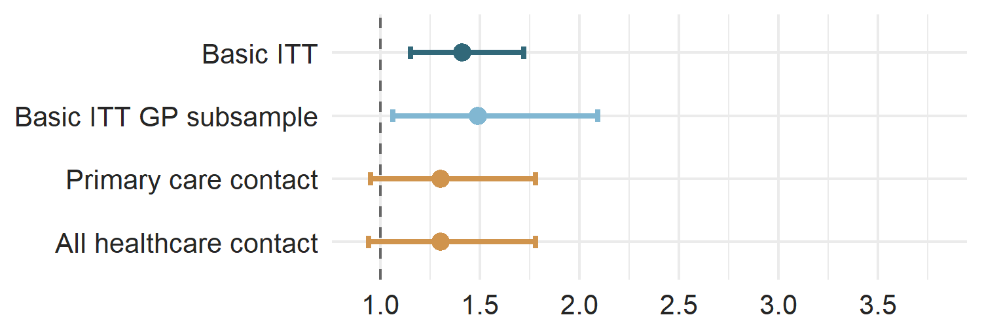

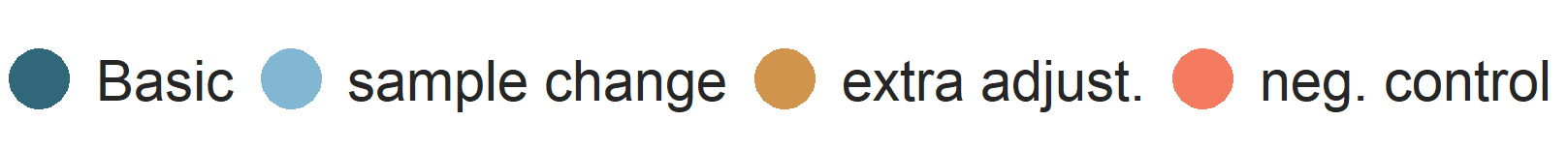


**Appendix S3**: summary of the present study and evidence for two causes of bias (confounding by healthcare utilisation and late detection of HL incidence) that would explain the detrimental effect of HA use on dementia.

Confounding by healthcare utilisation

- In the basic analysis, HA was positively associated with dementia risk (RR=1.43, CI=1.08-1.88).
- The adjustment for either primary- (RR=0.77, CI=0.44-1.33) or primary and secondary (RR=0.68, CI=0.44-1.18) healthcare utilisation reversed this trend. This was performed in a subsample of the data for whom primary-care data was available.
- Thus, healthcare utilisation is confounding the HA-dementia relationship.

Difference between subsample with primary care and the rest of the sample:

- When the basic analysis (where healthcare utilisation is not adjusted for) was repeated in the subsample with primary care data, the effect of HA on dementia also decreased (RR=1.22, CI=0.67-2.17).
- The only substantial difference between the subsample with primary care data and the rest of the sample was the prevalence of HA use (4.2% in primary-care subsample vs. 8.2% in the rest).

Cause for the above difference:

- As part of data cleaning, HL cases before UKB entry (pre-baseline cases) were removed before analysis.
- Because pre-baseline cases can only be those cases that are diagnosed via the EHR, and because most HL cases are caught in primary care, this data-cleaning step removed a higher proportion of participants from the subsample with primary care data than from the rest of the sample. Moreover, it removed participants that were diagnosed with HL relatively early. This could occur only in the subsample with primary care data, because the rest of the sample did not have data on primary care diagnoses. Thus, in the rest of the sample, such an equivalent early-diagnosed group of participants was detected as having HL only at study baseline (and was kept in the study).

Confounding by inaccurate dating of HL incidence:

- In a final set of sensitivity analyses, we did not remove participants with HL prior to baseline. Instead, we set their dates of HL/HA - if they occurred before baseline - to the date of baseline. We chose this approach because it is effectively what was happening in the subsample without primary care data: HL in participants that may have been diagnosed with HL in primary care before baseline are only being detected as having HL via self-report at baseline.
- In this alternative analytical sample, the prevalence of HA use (subsample with primary care data: 8.2%; entire sample: 8.5%), and the effect of HA use on the risk of dementia diagnosis (subsample with primary care data: RR=1.49, CI=0.95-1.78; entire sample: RR=1.41, CI=1.15-1.72) were much closer between the subsample with primary care data and the entire sample, suggesting that they were now more similar to each other.
- The additional adjustment for primary healthcare (RR=1.30, CI=0.95-1.78) or primary and secondary healthcare (RR=1.30, CI=0.94-1.78) decreased the effect size, but not as dramatically as when healthcare was adjusted for in the regularly cleaned sample.

Conclusions:

- Healthcare utilisation may be confounding the observed association.
- Mismeasurement of HL, or – more specifically – late detection of HL may be biasing the results. Much of the observed trend is likely driven by participants that were diagnosed with HL relatively early (pre-baseline) but remained undetected until baseline and were thus not removed from the study.
- It's unclear to what extent each of these mechanisms is biasing the results; other biases may also be at play. It is also unclear why participants diagnosed with HL prior to baseline bias the results towards a positive association between HA use and dementia.

**Supplementary references**

1. Brenowitz WD, Filshtein TJ, Yaffe K, et al. Association of genetic risk for Alzheimer disease and hearing impairment. *Neurology* 2020; **95**(16): e2225-e34.

2. Dawes P, Emsley R, Cruickshanks KJ, et al. Hearing loss and cognition: the role of hearing AIDS, social isolation and depression. *PLoS One* 2015; **10**(3): e0119616.

3. White IR, Royston P, Wood AM. Multiple imputation using chained equations: Issues and guidance for practice. *Stat Med* 2011; **30**(4): 377-99.

4. Hanlon P, Quinn TJ, Gallacher KI, et al. Assessing Risks of Polypharmacy Involving Medications With Anticholinergic Properties. *Ann Fam Med* 2020; **18**(2): 148-55.

5. van Buuren S, Groothuis-Oudshoorn K. mice: Multivariate Imputation by Chained Equations inR. *Journal of Statistical Software* 2011; **45**(3).

6. Townsend P. Deprivation. *Journal of Social Policy* 1987; **16**(2): 125-46.

7. Cox SR, Ritchie SJ, Fawns-Ritchie C, Tucker-Drob EM, Deary IJ. Structural brain imaging correlates of general intelligence in UK Biobank. *Intelligence* 2019; **76**: 101376.

8. Mur J, Marioni RE, Russ TC, Muniz-Terrera G, Cox SR. Anticholinergic burden in middle and older age is associated with lower cognitive function, but not with brain atrophy. *Br J Clin Pharmacol* 2023; **89**(7): 2224-35.

9. Elovainio M, Hakulinen C, Pulkki-Råback L, et al. Contribution of risk factors to excess mortality in isolated and lonely individuals: an analysis of data from the UK Biobank cohort study. *Lancet Public Health* 2017; **2**(6): e260-e6.

10. UK Biobank. UK Biobank Primary Care Linked Data Version 1.0. 2019.

11. Textor J, van der Zander B, Gilthorpe MS, Liśkiewicz M, Ellison GT. Robust causal inference using directed acyclic graphs: the R package 'dagitty'. *Int J Epidemiol* 2016; **45**(6): 1887-94.

12. Sävje F, Higgins MJ, Sekhon JS. Generalized Full Matching. *Political Analysis* 2020; **29**(4): 423-47.

13. Sävje F, Sekhon JS, Higgins MJ. Quick Generalized Full Matching. CRAN; 2023.

14. Chipman HA, George EI, McCulloch RE. BART: Bayesian additive regression trees. *The Annals of Applied Statistics* 2010; **4**(1).

15. Chipman DV, McCulloch R. Package ‘dbarts’. 2023. https://cran.r-project.org/web/packages/dbarts/dbarts.pdf.

16. Hernán MA, Robins JM. Using Big Data to Emulate a Target Trial When a Randomized Trial Is Not Available. *Am J Epidemiol* 2016; **183**(8): 758-64.

17. Audit Commission. Improving data quality in the NHS: Annual report on the PbR assurance programme. London, 2010.

18. Burns EM, Rigby E, Mamidanna R, et al. Systematic review of discharge coding accuracy. *J Public Health (Oxf)* 2012; **34**(1): 138-48.

19. Darke P, Cassidy S, Catt M, Taylor R, Missier P, Bacardit J. Curating a longitudinal research resource using linked primary care EHR data-a UK Biobank case study. *J Am Med Inform Assoc* 2022; **29**(3): 546-52.

20. Lipsitch M, Tchetgen Tchetgen E, Cohen T. Negative controls: a tool for detecting confounding and bias in observational studies. *Epidemiology* 2010; **21**(3): 383-8.

21. Yeo BSY, Song HJJMD, Toh EMS, et al. Association of Hearing Aids and Cochlear Implants With Cognitive Decline and Dementia. *JAMA Neurology* 2023; **80**(2).

22. Greifer N. Covariate Balance Tables and Plots. 2024. https://cran.r-project.org/web/packages/cobalt/cobalt.pdf.

23. Wood SN. Fast stable restricted maximum likelihood and marginal likelihood estimation of semiparametric generalized linear models. *Journal of the Royal Statistical Society Series B: Statistical Methodology*; **73**(1): 3-36.

24. Morisot A, Bessaoud F, Landais P, Rebillard X, Tretarre B, Daures JP. Prostate cancer: net survival and cause-specific survival rates after multiple imputation. *BMC Med Res Methodol* 2015; **15**: 54.

25. Rubin DB. Multiple Imputation for Nonresponse in Surveys; 1987.

1. Centre for Clinical Brain Sciences, University of Edinburgh, UK. [↑](#footnote-ref-1)
2. Centre for Genomic and Experimental Medicine, University of Edinburgh, UK. [↑](#footnote-ref-2)
3. Alzheimer Scotland Dementia Research Centre, University of Edinburgh, UK. [↑](#footnote-ref-3)
4. Department of Social Sciences, Institute for Research on Socio-Economic Inequality (IRSEI), University of Luxembourg, Esch-sur-Alzette, Luxembourg. [↑](#footnote-ref-4)
5. Department of General Internal Medicine and Psychosomatics, University Medical Centre Heidelberg, Heidelberg, Germany. [↑](#footnote-ref-5)
6. Department of Psychology, University of Edinburgh, UK. [↑](#footnote-ref-6)
7. Population Health Sciences Institute, Medical Sciences Faculty, Newcastle University, Newcastle, UK. [↑](#footnote-ref-7)
8. Institute of Clinical Medicine/Neurology, University of Eastern Finland, Kuopio, Finland. [↑](#footnote-ref-8)
9. Division of Clinical Geriatrics, Center for Alzheimer Research, Karolinska Institute, Stockholm, Sweden. [↑](#footnote-ref-9)
10. Ageing Epidemiology Research Unit, School of Public Health, Imperial College London, London, UK. [↑](#footnote-ref-10)
11. Speech and Hearing Sciences, Queen Margaret University, Edinburgh, UK. [↑](#footnote-ref-11)
12. Nuffield Department of Population Health, University of Oxford, Oxford, UK. [↑](#footnote-ref-12)
13. Centre for Dementia Prevention, University of Edinburgh, UK. [↑](#footnote-ref-13)
14. Ohio University, Athens, OH, USA. [↑](#footnote-ref-14)
